# Parametric methods outperformed non-parametric methods in comparisons of discrete numerical variables

**DOI:** 10.1186/1471-2288-11-44

**Published:** 2011-04-13

**Authors:** Morten W Fagerland, Leiv Sandvik, Petter Mowinckel

**Affiliations:** 1 Unit of Biostatistics and Epidemiology, Oslo University Hospital, Norway; 2 Department of Paediatrics, Oslo University Hospital, Ullevål, Norway

## Abstract

**Background:**

The number of events per individual is a widely reported variable in medical research papers. Such variables are the most common representation of the general variable type called discrete numerical. There is currently no consensus on how to compare and present such variables, and recommendations are lacking. The objective of this paper is to present recommendations for analysis and presentation of results for discrete numerical variables.

**Methods:**

Two simulation studies were used to investigate the performance of hypothesis tests and confidence interval methods for variables with outcomes {0, 1, 2}, {0, 1, 2, 3}, {0, 1, 2, 3, 4}, and {0, 1, 2, 3, 4, 5}, using the difference between the means as an effect measure.

**Results:**

The Welch U test (the T test with adjustment for unequal variances) and its associated confidence interval performed well for almost all situations considered. The Brunner-Munzel test also performed well, except for small sample sizes (10 in each group). The ordinary T test, the Wilcoxon-Mann-Whitney test, the percentile bootstrap interval, and the bootstrap-*t *interval did not perform satisfactorily.

**Conclusions:**

The difference between the means is an appropriate effect measure for comparing two independent discrete numerical variables that has both lower and upper bounds. To analyze this problem, we encourage more frequent use of parametric hypothesis tests and confidence intervals.

## Background

Categorical, or discrete, data are characterized by having a finite number of categories or values, whereas continuous data can take on any real value within a given range. For a categorical variable with more than two categories, we distinguish between nominal and ordered variables. Ordered variables have a natural ordering to the categories, for example, degree of pain classified as none, mild, moderate, or severe.

Sometimes, we have data that are essentially categorical, but with numerical properties, or numerical data that can take on only a small number of values. We shall refer to such data as discrete numerical [[1], p.11]. In medical research, discrete numerical data arise mostly in situations where we count the number of events per individual, such as the number of clinical visits, the number of adverse events, or the number of units of blood transfused. As a preliminary assessment of the prevalence of variables reporting the number of events, we considered all randomized, controlled trials (RCTs) published in January and February 2010 in the New England Journal of Medicine, Lancet, Journal of the American Medical Association, and BMJ. Out of a total of 52 papers, 24 (46%) papers reported at least one variable describing the number of events; 16 (31%) papers reported baseline variables, and 15 (29%) papers reported outcome variables.

Discrete numerical data are a blend between categorical and continuous data, and it is not obvious how to analyze such data. Of particular interest is how to compare two independent discrete numerical variables, a common problem in comparisons of two treatment or exposure groups. Should we analyze discrete numerical variables using methods for continuous or for ordered categorical data?

The main problem with using methods for ordered categorical data is information loss. Statistical methods for ordered categorical data do not treat the distance between values or categories as constant. Thus, we may not be getting the most out of the data. Even worse in that regard is to combine the outcomes into two categories and use methods for binary data. By doing so, we may throw away a lot of information. Poor power is often the result, and estimates may be inaccurate [2,3].

If we intend to analyze discrete numerical data without discarding relevant information, we should consider treating the variables as if they were continuous. Continuous variables with an approximately normal distribution are best analyzed using parametric methods for confidence intervals and hypothesis tests [4,5]. The usual alternative is a non-parametric test and a non-parametric or bootstrap confidence interval, or a transformation, for example, the logarithmic, prior to parametric methods. To decide if parametric methods are appropriate, the shapes of the underlying distributions are estimated by inspecting histograms, QQ-plots, and sample moments, or by using prior knowledge about the variable of interest. For discrete numerical variables, however, such tools may not be relevant because of the discrete nature of the underlying distributions.

In the survey of 52 RCTs published in four leading medical journals, 12 (23%) papers used statistical methods to compare discrete numerical variables between groups. All these 12 papers reported p-values, but only two papers reported effect measures and confidence intervals. Seven papers used non-parametric methods, three papers used parametric methods, one paper used negative binomial regression, one paper stated that the Cochran-Mantel-Haenszel test was used, and one paper used either the two-sample T test or the Wilcoxon-Mann-Whitney test.

The literature on statistical methods for analyzing discrete numerical variables is sparse. Newcombe [6] compares eight confidence interval methods for the mean of a single variable on the scale {0, 1, 2}, but does not consider comparisons of two independent variables. For comparing two continuous variables, on the other hand, a large body of literature exists. A relevant study for the hypothesis tests under investigation in this paper is Fagerland and Sandvik [7]. Confidence intervals for the difference in means of two independent continuous variables are considered in Zhou and Dinh [5] and Wilcox [[8], chapter 5]. Ordered categorical data is the topic of many papers, see for example Ryu and Agresti [9].

Returning to our survey, we counted nine different methods of presenting discrete numerical variables. The most common methods were to tabulate the data using categories such as {0, 1-3, 4+}, present the group means and standard deviations, or present medians and interquartile ranges (IQRs). Other methods included various combinations of means, medians, ranges, IQRs, and confidence intervals. Only two (8%) of 24 papers reported complete non-categorized data.

There is thus lack of a consensus on how to compare and present discrete numerical variables. In this paper, we shall investigate the performance of standard methods for continuous data applied to discrete numerical variables with outcomes such as {0, 1, 2, 3}. We assume that we are faced with samples from two independent random variables of equal type but with possibly different distributions. We shall further assume that we do not have a composite upper (or lower) limit, such as {0, 1, 2, 3, 4+}, where 4+ indicates outcomes with four or more events. The aim of this paper is to establish strong empirical evidence for recommending a suitable effect measure, methods for hypothesis testing and confidence intervals, and overall manner of presentation.

## Methods

### Effect measure

When we are dealing with two independent continuous variables, we are usually interested in estimating (and making inference about) the difference between some measure of the central tendencies. For symmetric distributions, most measures of central tendency, such as the arithmetic mean and the median, are equal. However, when distributions are skewed, different measures can vary substantially. The mean can be unduly influenced by outliers and may be a poor representation of the typical value. Choosing an appropriate measure of central tendency can then be quite difficult, particularly because software to analyze the optimal effect measure may not be readily available.

Fortunately, we seldom have the same problem with discrete numerical variables. When the variables have both lower and upper bounds, and when the range of possible values is quite limited, there will be no outliers or extreme values, at least not in the mathematical sense. There is thus no obvious added value of using, for example, the median or a trimmed mean as the measure of central tendency. Moreover, the median of discrete numerical variables often has a small number of possible values--five for a three-valued scale--which makes it an imprecise measure of central tendency and thus unsuitable for demonstrating less than large differences between the groups.

As long as the mean of the variable of interest makes sense for the subject matter, we consider it to be a suitable measure of central tendency and that the difference between the two means is an appropriate effect measure. Note that using the mean is only appropriate for outcome scales without composite limits. If scales such as {0, 1, 2, 3+} is used, where 3+ indicates outcomes with three or more events, the estimated group means may underestimate the true means. The resulting estimate of the difference between the means may then be difficult to interpret. When using methods for continuous data, we strongly recommend against using composite limits.

Another appropriate effect measure for comparing two independent groups is the relative effect, *p *= Pr(*X *<*Y *), where *X *and *Y *are random samples from the two groups. The relative effect is the probability that a random sample from one group is less than a random sample from the other group. If the groups are identically distributed, *p *= 1/2. Several rank-based methods, such as the Wilcoxon-Mann-Whitney test, is based on p or its generalization to tied values, *p *= Pr(*X *<*Y *) + 0.5. Pr(*X *= *Y *).

As an effect measure, the relative effect has the disadvantage that it is less specific than the difference between the means, and thereby more difficult to interpret. The relative effect can be a good alternative in situations where the mean is a poor estimate of central tendency. For discrete numerical variables with few possible values, the difference between the means is our preferred effect measure.

### Simulation study of hypothesis tests

For the main comparison of hypothesis tests, we consider four hypothesis tests: the two-sample T test, the modified T test for unequal variances (the Welch U test), the Wilcoxon-Mann-Whitney (WMW) test with adjustment for ties, and the Brunner-Munzel generalized WMW test. Details of the test statistics and their distributions can be found in Additional file 1.

We selected four outcome scales: {0, 1, 2}, {0, 1, 2, 3}, {0, 1, 2, 3, 4}, and {0, 1, 2, 3, 4, 5}. For each scale, we defined six underlying distributions, which we named uniform, normal, u-shaped, linear trend, step, and skewed. For the {0, 1, 2} scale, no skewed distribution was defined, but two different step distributions were used. Table 1 presents the expected values of the distributions. Further details are provided in Additional file 1: Web Figures 1-4. With six distributions, a total of 6^2 ^= 36 different combinations are possible. For 12 of these combinations, the expected values are equal, and for the remaining 24, the difference between expected values ranges from small (linear trend versus step) to large (uniform/normal/u-shaped versus skewed/step). When the two sample sizes are equal, the order of the distributions is irrelevant and the number of distribution combinations is reduced to 21, nine with equal expected values and 12 with unequal expected values.

**Table 1:** Expected values for the six distributions in the simulation study.

	Outcome scale			
Distribution	{0, 1, 2}	{0, 1, 2, 3}	{0, 1, 2, 3, 4}	{0, 1, 2, 3, 4, 5}
Uniform	1.0	1.5	2.0	2.5
Normal	1.0	1.5	2.0	2.5
U-shaped	1.0	1.5	2.0	2.5
Linear trend	0.63	1.0	1.25	1.625
Step	0.8 & 0.6*	0.9	1.1	1.6
Skewed	*	0.6	0.75	0.88

*An additional step distribution was used instead of the skewed distribution.

Nine different sample size combinations were used, ranging from (10, 10) to (100, 100) and including both equal and unequal sizes. The nominal significance level was 5% and 100 000 replications were used. Table 2 shows a summary of the simulation setup.

**Table 2:** Summary of the simulation setup (hypothesis tests).

Hypothesis tests	T: two-sample T test
	U: Welch U test (T test for unequal variances)
	WMW: Wilcoxon-Mann-Whitney test
	BM: Brunner-Munzel test
Outcome scales	{0, 1, 2}, {0, 1, 2, 3}, {0, 1, 2, 3, 4}, {0, 1, 2, 3, 4, 5}
Distributions	Uniform, normal, u-shaped, linear trend, step, skewed
Sample sizes (*m*, *n*)	(10,10), (25,25), (50,50), (100,100), (25,10), (50,10), (100,50), (100,25), (100,10)
Nominal sig.level	5%
Replications	100 000
Programming language	Matlab [10] with the Statistics Toolbox

It has been suggested that a permutation test based on the Brunner-Munzel test statistic is appropriate for comparing small-sample discrete data [11]. We assessed this test--using 10 000 random permutations for each calculated test--in a small separate simulation study. Only the sample sizes *m *= *n *= 10, the nine combinations of distributions with equal expected values, and 10 000 replications were used.

### Simulation study of confidence intervals

The variance estimates used in the T and the Welch U tests are frequently used for the computation of confidence intervals. We refer to those intervals as the T confidence interval and the Welch U confidence interval. These are reported in most general purpose statistical software packages. It is clear from the results of the simulation study of hypothesis tests (see Results section) that the variance estimate for the T test is inaccurate for most situations where the sample sizes are unequal. As such, we include the Welch U confidence interval, but not the T confidence interval, in our investigation. In like manner, we do not consider non-parametric confidence intervals based on the WMW statistic because the WMW test performed poorly in the simulation study of hypothesis tests.

As alternatives to the Welch U confidence interval, we consider two simple bootstrap intervals: the percentile bootstrap and the bootstrap-*t *[12], both with 2000 samples.

Bootstrap confidence intervals are computationally demanding. For the computation of a single interval, there are, of course, no obstacles with modern computer power. In a simulation study, however, we need to compute several thousand intervals, which, accumulated over various settings, can be quite time consuming. The full simulation setup from the previous section is thereby reduced for the investigation of the confidence intervals. We consider only four sample size combinations and use 10 000 replications. The outcome scales and the distributions are unchanged. We summarize the new simulation setup in Table 3.

**Table 3:** Summary of the simulation setup (confidence intervals).

Confidence intervals	U: the Welch U (T adjusted for unequal variances)
	PB: percentile bootstrap (2000 samples)
	Bt: bootstrap-*t *(2000 samples)
Outcome scales	{0, 1, 2}, {0, 1, 2, 3,}, {0, 1, 2, 3, 4}, {0, 1, 2, 3, 4, 5}
Distributions	Uniform, normal, u-shaped, linear trend, step, skewed
Sample sizes (*m*, *n*)	(10, 10), (50, 50), (25, 10), (100, 25)
Nominal confidence level	95%
Replications	10 000
Programming language	Matlab [10] with the Statistics Toolbox

## Results

### Hypothesis tests

For each combination of outcome scale, sample sizes, and distributions, the rejection rates of the tests were recorded. When the expected values of the two distributions were equal, the rejection rates estimated the true significance level of the tests for the hypothesis of equal means. For distributions with unequal expected values, the rejection rates estimated the power of the tests to detect departures from equality of means.

For the assessment of true significance levels, we defined robustness criteria. If, for a given setting, the estimated true significance level of one of the tests deviated less than 10% from the nominal level, the test was defined as 10% robust. Similarly, if the estimated true significance level deviated less than 20% from the nominal level, the test was defined as 20% robust. A test with true significance levels that deviated more than 20% from the nominal level was defined as nonrobust. For a nominal significance level of 5%, the three robustness categories were

• 10% robust: 4.5 ≤ *p *≤ 5.5

• 20% robust: 4.0 ≤ *p *≤ 5.0

• Nonrobust: *p *< 4.0 or *p *> 6.0

where *p *denotes the estimated true significance level. These robustness criteria have been used previously [7,13]. We refer to Bradley [14] for a general discussion of robustness criteria.

We present the full results of the main simulation study in Additional file 2: Web Tables 3-38. Table cells are colored green, yellow, and red to indicate 10% robustness, 20% robustness, and nonrobustness, respectively.

To facilitate interpretation of the results, we present a summary of the results in Tables 4-5 and Additional file 2: Web Tables 1-2. The summery tables for the outcome scales {0, 1, 2, 3, 4} (Additional file 2: Web Table 1) and {0, 1, 2, 3, 4, 5} (Additional file 2: Web Table 2) were placed in Additional file 2 because the results were similar to the results from the outcome scale {0, 1, 2, 3} (Table 5). Columns 2-5 show the mean deviation of the true significance level from the nominal significance level for the four main tests. The mean value is calculated over all combinations of distributions with equal expected values. For each sample size combination, the test with the smallest mean deviation (the best test in that situation) is marked with bold type. The test with the largest mean deviation (the worst performing test) is marked with italic type.

**Table 4:** Simulation results (hypothesis tests) for the outcome scale {0, 1, 2}.

	Mean deviation from 5%				Relative power (%)			
Sample size	T	U	WMW	BM	T	U	WMW	BM
10, 10	**0.19**	0.30	0.60	*0.75*	90.2	88.4	*86.7*	**100.0**
25, 25	0.10	**0.08**	*0.33*	0.23	96.2	*96.1*	99.1	**100.0**
50, 50	**0.04**	0.05	*0.23*	0.11	97.5	*97.4*	**100.0**	99.7
100, 100	**0.07**	0.07	*0.27*	0.09	98.3	*98.3*	**100.0**	99.7
25, 10	*1.27*	**0.29**	1.24	0.42	97.9	*94.1*	96.7	**100.0**
50, 10	1.85	**0.35**	*1.90*	0.50	98.2	*91.2*	**100.0**	98.3
100, 50	0.87	**0.06**	*0.89*	0.08	98.0	*97.4*	**100.0**	99.2
100, 25	1.63	**0.11**	*1.64*	0.17	97.1	*93.7*	**100.0**	97.3
100, 10	2.25	**0.36**	*2.27*	0.51	97.1	*86.9*	**100.0**	94.3

**Table 5:** Simulation results (hypothesis tests) for the outcome scale {0, 1, 2, 3}.

	Mean deviation from 5%				Relative power (%)			
Sample size	T	U	WMW	BM	T	U	WMW	BM
10, 10	**0.24**	0.28	0.55	*0.59*	**100.0**	96.8	*89.9*	98.4
25, 25	0.06	**0.06**	*0.35*	0.19	**100.0**	99.8	*94.8*	95.6
50, 50	**0.06**	0.06	*0.33*	0.11	**100.0**	99.9	96.1	*95.8*
100, 100	0.07	**0.07**	*0.33*	0.10	**100.0**	100.0	97.7	*97.3*
25, 10	*1.35*	**0.27**	1.34	0.44	**100.0**	95.4	*92.7*	*94.6*
50, 10	*2.05*	**0.39**	2.00	0.51	**100.0**	91.5	93.4	*91.3*
100, 50	1.00	**0.04**	*1.03*	0.06	**100.0**	99.7	96.7	*96.1*
100, 25	*1.78*	**0.09**	1.77	0.12	**100.0**	97.6	95.6	*94.1*
100, 10	*2.55*	**0.41**	2.42	0.50	**100.0**	*88.4*	93.6	89.0

For the permutation test, the rejection rates for the outcome scale {0, 1, 2} ranged from 6.5% to 9.8%. The mean deviation from 5% was 2.89. When the other outcome scales were used, the rejection rates decreased but were still quite high and greater than those of the other tests.

Columns 6-9 display the relative power of the four main tests. The relative power is calculated by adding the per cent rejection rates for all combinations of distributions with unequal expected values and using the largest sum as the reference value. The greatest power is marked with bold type and the lowest power is marked with italic type.

The proportions of true significance levels that were nonrobust--averaged over all outcome scales, sample sizes, and distribution combinations--were 31% (T), 6.8% (U), 34% (WMW), and 1.8% (BM).

### Confidence intervals

For each calculated confidence interval, we note three items: (i) does the interval contain the true difference between the means? (ii) the length of the interval; (iii) does the confidence limits extend beyond the maximum possible difference for the scale? For example, when using the outcome scale {0, 1, 2}, the maximum possible difference between the means is ±2.

The first item is used to estimate the coverage probability of the confidence intervals. The coverage probability should be close the nominal confidence level of 95%. If two or more confidence intervals have similar coverage probabilities, we can compare the intervals' lengths. Note that an interval with a low coverage probability can be expected to be shorter than an interval with a coverage probability close to the nominal level. From the third item, we compute the overshoot rate--the rate at which the intervals give nonsensical results.

The full results of the simulation study are given in Additional file 2: Web Tables 39-54. As before, green table cells indicate 10% robustness (94.5 ≤ *c *≤ 95.5), yellow table cells indicate 20% robustness (94.0 ≤ *c *≤ 96.0), and red cells indicate nonrobustness (*c *< 94.0 or *c *> 96.0), where *c *denotes the estimated coverage probability.

We present a summary of the results in Table 6. Each table cell is the per cent mean coverage probability or the mean interval length over all combinations of distributions.

**Table 6:** Simulation results (confidence intervals).

	**Mean coverage prob**.			Mean interval length		
Sample size	U	PB	Bt	U	PB	Bt
Outcome scale {0, 1, 2}						
10, 10	95.2	93.2	95.8	1.45	1.27	1.49
50, 50	94.8	94.7	94.8	0.61	0.60	0.61
25, 10	94.3	92.5	94.1	1.21	1.07	1.19
100, 25	95.0	94.3	94.6	0.70	0.66	0.69
Outcome scale {0, 1, 2, 3}						
10, 10	95.0	93.0	95.8	1.95	1.71	2.01
50, 50	94.8	94.6	94.8	0.82	0.80	0.82
25, 10	94.5	92.5	94.3	1.63	1.44	1.61
100, 25	94.8	94.0	94.4	0.94	0.89	0.92
Outcome scale {0, 1, 2, 3, 4}						
10, 10	94.9	92.7	95.8	2.36	2.05	2.43
50, 50	94.8	94.5	94.8	0.99	0.97	0.99
25, 10	94.5	92.5	94.5	1.96	1.73	1.95
100, 25	94.7	94.1	94.4	1.13	1.07	1.11
Outcome scale {0, 1, 2, 3, 4, 5}						
10, 10	94.9	92.5	95.8	2.84	2.47	2.94
50, 50	94.8	94.5	94.8	1.20	1.17	1.20
25, 10	94.5	92.5	94.5	2.37	2.09	2.35
100, 25	94.7	94.0	94.4	1.35	1.29	1.34

Mean coverage probabilities in per cent.

The proportions of confidence intervals that were nonrobust--averaged over all outcome scales, sample sizes, and distribution combinations--were 6.6% (U), 60% (PB), and 18% (Bt).

The overshoot rate was zero for all intervals for all settings.

### Recommendations: hypothesis test

For the outcome scale {0, 1, 2}, both the Welch U test and the Brunner-Munzel test had true significance levels that were close to the nominal level, although the Brunner-Munzel test did not perform well for the smallest sample size combination (*m *= *n *= 10). Among the two tests, the Brunner-Munzel test had superior power. The WMW test had true significance levels close to the nominal level when both samples were drawn from identical distributions. However, it was severely nonrobust for unequal distributions, particularly when the sample sizes were unequal. In these cases, the true significance level of the WMW test was sometimes above the nominal level and sometimes below the nominal level. The WMW test is thus not a reliable test of equality of distributions as it would have poor power in many situations. The ordinary T test performed similarly to the WMW test and neither test can be recommended. Nor can we recommend the Neubert-Brunner permutation test, which performed poorly for *m *= *n *= 10. Instead, we recommend the Brunner-Munzel test, expect for small sample sizes where the Welch U test is our first choice.

The results from using the outcome scales {0, 1, 2, 3}, {0, 1, 2, 3, 4}, and {0, 1, 2, 3, 4, 5} were similar and are considered together. The T and WMW tests were usually robust when distributions were equal, but for unequal distributions, the nonrobustness of both tests increased with increasing number of outcome values. Of all tests considered, the T test had the greatest power. The Welch U test had superior robustness properties and performed well both for equal and unequal sample sizes, except for some cases where the sample size difference was large. Its power was often quite close to that of the T test. The Brunner-Munzel test performed generally well, but were usually slightly inferior to the Welch U test. We recommend the Welch U test and note that the Brunner-Munzel test can be a useful alternative. The T test and the WMW test are not recommended.

### Recommendations: confidence intervals

The results for all three confidence interval methods were consistent over all the outcome scales. The coverage probability for the percentile bootstrap interval was considerably below the nominal level for most situations, and as such, we cannot recommend its use. The bootstrap-*t *interval performed well when both sample sizes were 50. However, for the other sample size combinations, the coverage probability often deviated markedly, and in both directions, from the nominal level. In general, the Welch U interval had coverage probabilities close to the nominal level, although some distribution combinations produced coverage probabilities in the range 93-94% when the sample sizes were unequal. The interval lengths of the Welch U and the bootstrap-*t *intervals were similar.

Overall, the Welch U confidence interval performed better than the two bootstrap intervals and we recommend its use.

### Recommendations: presentation of results

Reporting guidelines recommend--and many journals now require--that the principal analyses of a study are presented with the three key statistical items: point estimate, confidence interval, and *p*-value [15,16]. No exception should be made for discrete numerical data. In addition, given the discrete nature of the data, a 2 × *g *table (where *g *is the number of outcome values) representing the entire body of data can easily be presented, at least when the number of outcome values is small. This will show the distribution of data across the possible outcome values and allow readers to perform alternative analyses. Unfortunately, such reporting is rarely done in practice. The usual method of presentation is to report the group means or medians with either the standard deviations or the interquartile ranges. In the next section, we consider data from two clinical trials and illustrate how discrete numerical data can be analyzed and presented.

### Clinical example: postcard intervention to reduce repetition of deliberate self poisoning

In a randomized controlled trial of patients hospitalized for deliberate self poisoning, Carter et al. [17] randomized 378 patients to an intervention group and 394 patients to a control group. All patients received standard treatment. In addition, the patients in the intervention group received eight postcards over 12 months. The main outcome measures were the proportion of patients with one or more repeat episodes of deliberate self poisoning and the number of episodes of deliberate self poisoning per patient during 12 months. The latter outcome measure is discrete numerical. As the maximum number of observed episodes per patient was four, the outcome scale was {0, 1, 2, 3, 4}.

The authors detected a difference between the sexes and undertook subgroup analyses for men and women separately. Complete data is available for men (Table 7), but not for women. The distributions are highly skewed, but appear to be quite similar in the two groups. The sample size is large and--given the results from the simulations studies--it appears appropriate to use the Welch U confidence interval and test. The difference between the means is 0.0059 with 95% confidence interval -0.14 to 0.15. The *p*-value is 0.94. There is thus no evidence of a treatment effect in men.

**Table 7:** Number of repeat episodes of deliberate self poisoning in men.

	Number of repetitions					
	0	1	2	3	4	mean (std)
Control group (*n *= 102)	86	13	2	0	1	0.21 (0.57)
Postcard group (*n *= 145)	125	13	10	2	0	0.20 (0.56)

Source: Carter et al. [17], Table 3.

The authors of this trial used negative binomial regression to compare the risk of repeat episodes in the two groups. They found that the incidence risk ratio for the postcard group was 0.97 (95% confidence interval 0.48 to 1.98, *p *= 0.94)--a similar result to the one above. The negative binomial distribution is based on a sequence of Bernoulli trials where the probability of an event (an episode of self poisoning) is constant. We are not convinced that it is appropriate to treat the number of repeat episodes of deliberate self poisoning as a sequence of event/non-event trials. Furthermore, it does not seem likely that the probability of an episode of self poisoning is independent of the number of previous episodes.

### Clinical example: intensive versus standard asthma education program

Does an intensive asthma education program reduce the number of visits to the emergency department and the number of hospitalizations for asthmatic children? Ng et al. [18] examined this issue by randomizing 100 children with an acute attack of asthma to either an intensive asthma education program (*n *= 55) or a standard asthma education program (*n *= 45).

The number of visits to the emergency department during the first three months after discharge from the pediatric department was observed on a {0, 1, 2, 3, 4} scale. The results are given in Table 8. The sample size in this trial is similar to the (50, 50) used in the simulation studies, where the Welch U interval and test are recommended. The difference between the means is 0.83 with 95% confidence interval 0.36 to 1.30. The *p*-value is 0.0007. It appears that the intensive program reduces the number of visits to the emergency department by almost one visit per patient as compared with the standard program. The authors further found that the intensive program reduced the number of hospitalizations, but not the number of unscheduled visits to the general practitioners.

**Table 8:** Number of visits to the emergency department.

	Number of visits					
	0	1	2	3	4	mean (std)
Standard program (*n *= 45)	19	10	7	3	6	1.27 (1.42)
Intensive program (*n *= 55)	39	8	8	0	0	0.44 (0.74)

Source: Ng et al. [18], Table 3.

Finally, we note a small discrepancy between our calculations and the results reported in Ng et al. [18]. No effect measure and confidence interval were presented in that paper, but the *p*-value was given as 0.004 with either the T test or the Wilcoxon-Mann-Whitney test. We get *p *= 0.0003 with the ordinary T test, *p *= 0.0007 with the Welch U test, and *p *= 0.001 with the Wilcoxon-Mann-Whitney test.

## Discusssion

We have considered how to compare two independent discrete numerical variables, a problem for which the difference between the two means is a suitable effect measure. Through two simulation studies, we find that the Welch U test and confidence interval can be recommended for statistical inference. The Brunner-Munzel test can also be recommended--except for small sample sizes--however, if it is used in conjunction with the Welch U confidence interval, consistency between the test and confidence interval is not guaranteed. We prefer a unified approach, where test and confidence interval is based on similar principles. We further recommend that a table summarizing all the data is presented, at least for primary outcome variables.

We are not aware of any other paper that explicitly deal with the problem of comparing two independent discrete numerical variables by using statistical methods for continuous data. Our small survey illustrates that comparisons and presentations of such variables are performed in various fashions in the medical research literature. Few reported effect measures and confidence intervals, and few presented complete data. Non-parametric methods--which were outperformed by their parametric counterparts in our study--were the most commonly used statistical methods. As such, this paper provides a necessary justification for using standard parametric methods for continuous data when comparing discrete numerical variables.

If we compare the recommendations in this paper with the results from studies of two continuous variables, there is some, but not complete, agreement. For approximately normal distributed variables, the two-sample T test and confidence interval are well known to be the optimal methods for comparing the means. It is for nonnormal data, and especially skewed data, that alternative methods might be preferable. In a study of hypothesis tests, Fagerland and Sandvik [7] found that no test can be recommended for all situations, although the Welch U test performed best overall. They recommend that the selection of test is based on a thorough investigation of distribution properties. Zhou and Dinh [5] compared the ordinary T interval, the bootstrap-*t *interval, the bias-corrected and accelerated interval, and three intervals based on transformation of the *t*-statistic. They found that the bootstrap-t interval gave consistent and best coverage, and that two of the transformation intervals were better than the ordinary T interval. The most noticeable difference between these two studies and the present one is the performance of the bootstrap-*t *interval. In our study, the bootstrap-*t *interval performed poorly for small sample sizes and for unequal sample sizes compared with the Welch U interval. This difference in performance may be due to the fact that Zhou and Dinh used continuous distributions, whereas we used discrete distributions. Unfortunately, Zhou and Dinh [5] did not include the Welch U interval in their simulation study, thus a comparison of the Welch U and the bootstrap-*t *intervals for continuous distributions is not available. Regarding the poor performance of the WMW test, Lehmann [[19], Section 1.4 and p.60] notes some concerns both for the exact and the asymptotic WMW test in the presence of many ties.

Sometimes, it might be of interest to compare other aspects of the variables besides the means. In the example of intensive versus standard asthma education program, for example, not only the means but also the standard deviations are quite different (Table 8). The Kolmogorov-Smirnov test is commonly used to test the hypothesis that two variables have identical distributions. In the presence of many ties--as is the case with discrete numerical data--Neuhäuser [20] suggests a permutation test based on the Baumgartner-Weiß-Schindler statistic, and shows that this test is superior to the Kolmogorov-Smirnov test and five other tests for the hypothesis of equal distributions.

Our two simulation studies were limited to the outcome scales {0, 1, 2}, {0, 1, 2, 3}, {0, 1, 2, 3, 4}, and {0, 1, 2, 3, 4, 5}. Nevertheless, we extend the recommendations to wider discrete numerical outcome scales--provided they have both upper and lower limits--with some confidence. This is due to two reasons: (i) the results from the simulation studies were quite similar for the outcome scales {0, 1, 2, 3}, {0, 1, 2, 3, 4}, and {0, 1, 2, 3, 4, 5}; (ii) as long as the variables have lower and upper bounds that are not too far apart, the mean will be an appropriate measure of central tendency. We thereby expect that the methods under investigation in this study will perform similarly on variables with outcomes such as {0, 1,...., 10}.

One benefit of being able to use simple parametric tests and confidence intervals for the comparison of two samples is that there is a natural way of generalizing the approach to situations with more than two samples and to the regression setting. It would be useful to perform a study to assess the performance of linear regression models with discrete numerical dependent variables. Based on the results from this study, we are optimistic about the prospects from such an investigation.

## Conclusions

In the medical research literature, discrete numerical variables--usually reporting the number of events per individual--are common. Until now, no studies has assessed the performance of parametric methods for comparing such variables. In our study, the Welch U test and confidence interval outperformed the Wilcoxon-Mann-Whitney test and two simple bootstrap intervals. We encourage more frequent use of parametric methods for comparing discrete numerical variables.

## Competing interests

The authors declare that they have no competing interests.

## Authors' contributions

MWF conceived of the study, designed and carried out the literature survey, designed and carried out the simulation studies, wrote an initial draft, and worked on the production of final draft. LS conceived of the study, participated in the design of the simulation studies, and worked on the production of final draft. PM conceived of the study, participated in the design of the simulation studies, and worked on the production of final draft. All authors read and approved the final manuscript.

## Pre-publication history

The pre-publication history for this paper can be accessed here:

http://www.biomedcentral.com/1471-2288/11/44/prepub

## Supplementary Material

Additional file 1 **Test statistics**. Details of the test statistics used in the simulation studies. Click here for file

Additional file 2 **Figures and tables**. Figures showing the distributions used in the simulation studies and detailed results from the two simulation studies. Click here for file
