# Supplementary material for: Parametric methods outperformed non-parametric methods in comparisons of discrete numerical variables
Source: BMC Med Res Methodol. 2011 Apr 13;11:44. doi: 10.1186/1471-2288-11-44 (PMC3097007; doi:10.1186/1471-2288-11-44)
Supplement: Additional file 1 — Test statistics. Details of the test statistics used in the simulation studies. [file 1471-2288-11-44-S1.PDF]

Additional file 1: Test statistics

Parametric methods outperformed non-parametric methods in comparisons of discrete numerical variables

Morten W. Fagerland[1] * Leiv Sandvik[1] Petter Mowinckel[2]

[1]Unit of Biostatistics and Epidemiology, Oslo University Hospital, Norway

[2]Department of Paediatrics, Oslo University Hospital, Ullevål, Norway

*Corresponding author. E-mail: morten.fagerland@medisin.uio.no

Notation

Suppose that we have two independent samples: X with m observations and Y with n observations. The estimated means and sample variances are:

$$\bar{X} = \frac{1}{m}\sum_{i=1}^{m} X_i, \qquad \bar{Y} = \frac{1}{n}\sum_{i=1}^{n} Y_i,$$

and

$$S_X^2 = \frac{1}{m-1}\sum_{i=1}^{m}(X_i - \bar{X})^2, \qquad S_Y^2 = \frac{1}{n-1}\sum_{i=1}^{n}(Y_i - \bar{Y})^2.$$

The two-sample T test

- Pooled sample standard deviation: $S_p^2 = \dfrac{(m-1)S_X^2 + (n-1)S_Y^2}{m+n-2}$
- Test statistic: $T = \dfrac{\bar{X} - \bar{Y}}{S_p\sqrt{1/m + 1/n}}$
- Reference distribution: t-distribution with $m + n - 2$ degrees of freedom

The Welch U test

- Test statistic: $U = (\bar{X} - \bar{Y}) \Big/ \sqrt{\dfrac{S_X^2}{m} + \dfrac{S_Y^2}{n}}$
- Reference distribution: t-distribution with f_U degrees of freedom
- $f_U = \left(\dfrac{S_X^2}{m} + \dfrac{S_Y^2}{n}\right)^2 \Big/ \left(\dfrac{S_X^4}{m^3 - m^2} + \dfrac{S_Y^4}{n^3 - n^2}\right)$

The (approximate) Wilcoxon-Mann-Whitney test

- Calculate R_X, the sum of the ranks in sample X
- Calculate $W_X = mn + m(m+1)/2 - R_X$
- Test statistic: $W = \big(W_X - mn/2\big) \Big/ \sqrt{mn(m+n+1)/12}$
- Reference distribution: standard normal

The Brunner-Munzel test

- Pool the data from X and Y
- Calculate $M_X = M_X^1, M_X^2, \ldots, M_X^m$ and $M_Y = M_Y^1, M_Y^2$, the midranks[1] associated with the samples X and Y
- Calculate $\bar{M}_X$ and $\bar{M}_Y$, the means of the midranks
- Calculate $V_X = V_X^1, V_X^2, \ldots, V_X^m$ and $V_Y = V_Y^1, V_Y^2, \ldots, V_Y^n$, the midranks within each sample
- Calculate $SB_X^2 = \dfrac{1}{m-1} \sum_{i=1}^{m} \left(M_X^i - V_X^i - \bar{M}_X + \dfrac{m+1}{2} \right)^2$
- Calculate $SB_Y^2 = \dfrac{1}{n-1} \sum_{i=1}^{n} \left(M_Y^i - V_Y^i - \bar{M}_Y + \dfrac{n+1}{2} \right)^2$
- Test statistic: $B = \left(\bar{M}_Y - \bar{M}_X \right) \Big/ (m+n) \sqrt{SB_X^2/mn^2 + SB_Y^2/m^2 n}$
- Reference distribution: t-distribution with f_B degrees of freedom
- $f_B = \left(\dfrac{SB_X^2}{n} + \dfrac{SB_Y^2}{m} \right)^2 \Big/ \left(\dfrac{SB_X^4}{n^2(m-1)} + \dfrac{SB_Y^4}{m^2(n-1)} \right)$

[1]Midranks are equal to ranks when there are no tied values. For tied values, the midranks are the average of their ranks.
