# Supplementary material for: Parametric methods outperformed non-parametric methods in comparisons of discrete numerical variables
Source: BMC Med Res Methodol. 2011 Apr 13;11:44. doi: 10.1186/1471-2288-11-44 (PMC3097007; doi:10.1186/1471-2288-11-44)
Supplement: Additional file 2 — Figures and tables. Figures showing the distributions used in the simulation studies and detailed results from the two simulation studies. [file 1471-2288-11-44-S2.PDF]

# Additional file 2: Figures and tables

# Parametric methods outperformed non-parametric methods in comparisons of discrete numerical variables

Morten W. Fagerland[1]* Leiv Sandvik[1] Petter Mowinckel[2]

[1]Unit of Biostatistics and Epidemiology, Oslo University Hospital, Norway

[2]Department of Paediatrics, Oslo University Hospital, Ullevål, Norway

*Corresponding author. E-mail: morten.fagerland@medisin.uio.no

**Contents**

| Page | Figure/Table | Description |
|---|---|---|
| 2 | Web Figure 1 | The distributions for the outcome $\{0, 1, 2\}$ |
| 3 | Web Figure 2 | The distributions for the outcome $\{0, 1, 2, 3\}$ |
| 4 | Web Figure 3 | The distributions for the outcome $\{0, 1, 2, 3, 4\}$ |
| 5 | Web Figure 4 | The distributions for the outcome $\{0, 1, 2, 3, 4, 5\}$ |
| 6 | Web Tables 1–2 | Summary of the simulation results for the outcomes $\{0, 1, 2, 3, 4\}$ and $\{0, 1, 2, 3, 4, 5\}$ |
| 7–15 | Web Tables 3–11 | Simulation results (hypothesis tests) for the outcome $\{0, 1, 2\}$ |
| 16–24 | Web Tables 12–20 | Simulation results (hypothesis tests) for the outcome $\{0, 1, 2, 3\}$ |
| 25–33 | Web Tables 21–29 | Simulation results (hypothesis tests) for the outcome $\{0, 1, 2, 3, 4\}$ |
| 34–42 | Web Tables 30–38 | Simulation results (hypothesis tests) for the outcome $\{0, 1, 2, 3, 4, 5\}$ |
| 43–46 | Web Tables 39–42 | Simulation results (confidence intervals) for the outcome $\{0, 1, 2\}$ |
| 47–50 | Web Tables 43–46 | Simulation results (confidence intervals) for the outcome $\{0, 1, 2, 3\}$ |
| 51–54 | Web Tables 47–50 | Simulation results (confidence intervals) for the outcome $\{0, 1, 2, 3, 4\}$ |
| 55–58 | Web Tables 51–54 | Simulation results (confidence intervals) for the outcome $\{0, 1, 2, 3, 4, 5\}$ |

**Notation**

$X$ and $Y$ are two independent random variables from which we have samples of size $m$ and $n$, respectively. Let $E(X)$ and $E(Y)$ denote the expected values of the distributions of $X$ and $Y$. The nominal significance level is 5% and the nominal confidence level is 95%. Let $p$ denote the estimated true significance level (per cent). Let $c$ denote the estimated coverage probability (per cent).

**Web Tables 3–38** Green table cells (10% robust): $4.5 \leq p \leq 5.5$, yellow cells (20% robust): $4.0 \leq p \leq 6.0$, red cells (nonrobust): $p < 4.0$ or $p > 6.0$. For each outcome scale and sample size combination, the test with the smallest mean deviation of true significance levels from the nominal level is marked with bold type. The test with the largest mean deviation is marked with italic type. Similarly, for the power simulations, the test with the greatest power is marked with bold type and the test with lowest power is marked with italic type.

**Web Tables 39–54** Green table cells (10% robust): $94.5 \leq c \leq 95.5$, yellow cells (20% robust): $94.0 \leq c \leq 96.0$, red cells (nonrobust): $c < 94.0$ or $c > 96.0$.

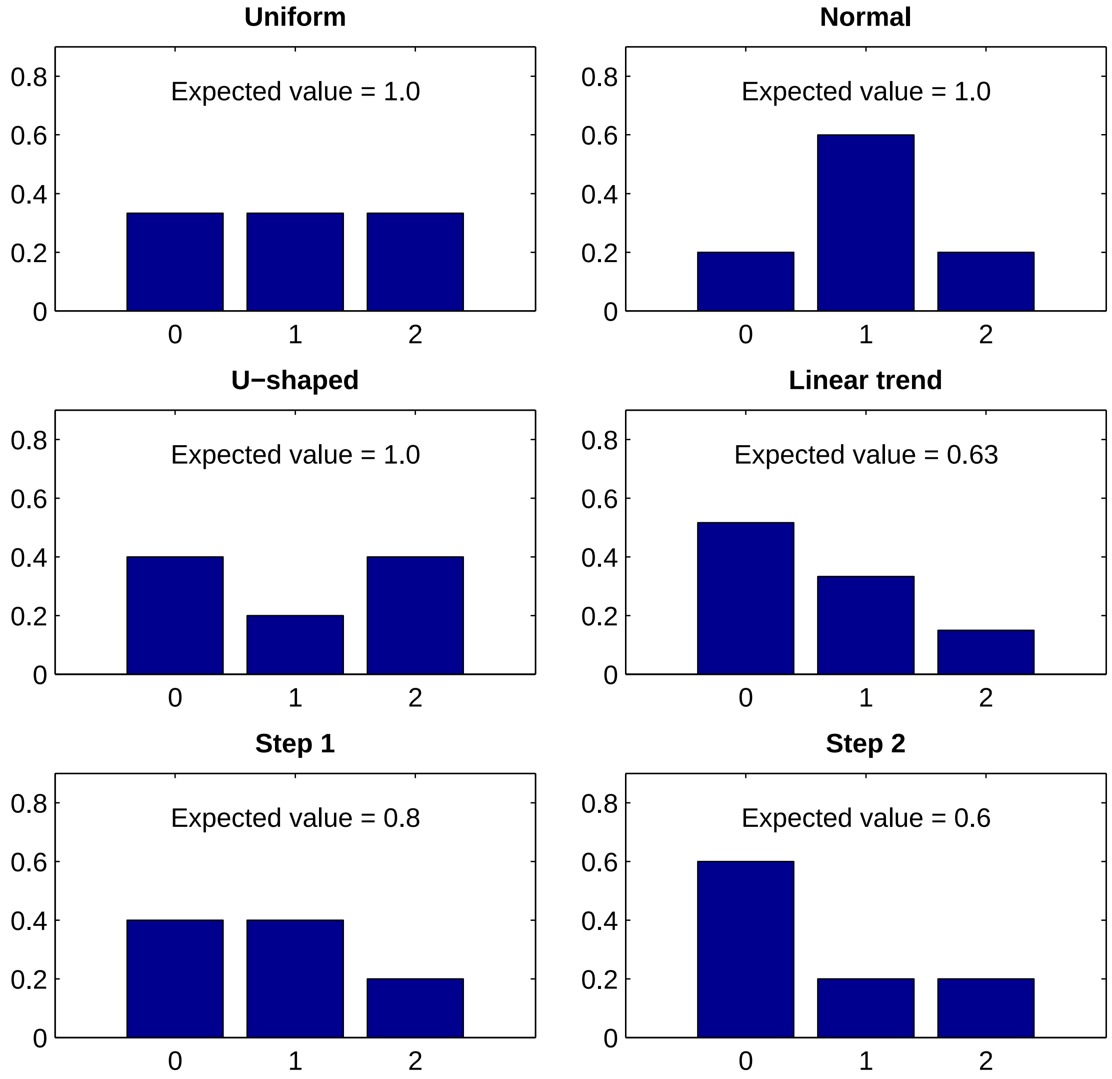


Web Figure 1: The six distributions used for the outcome scale $\{0, 1, 2\}$. Plots of probabilities ($y$-axis) against outcomes ($x$-axis).

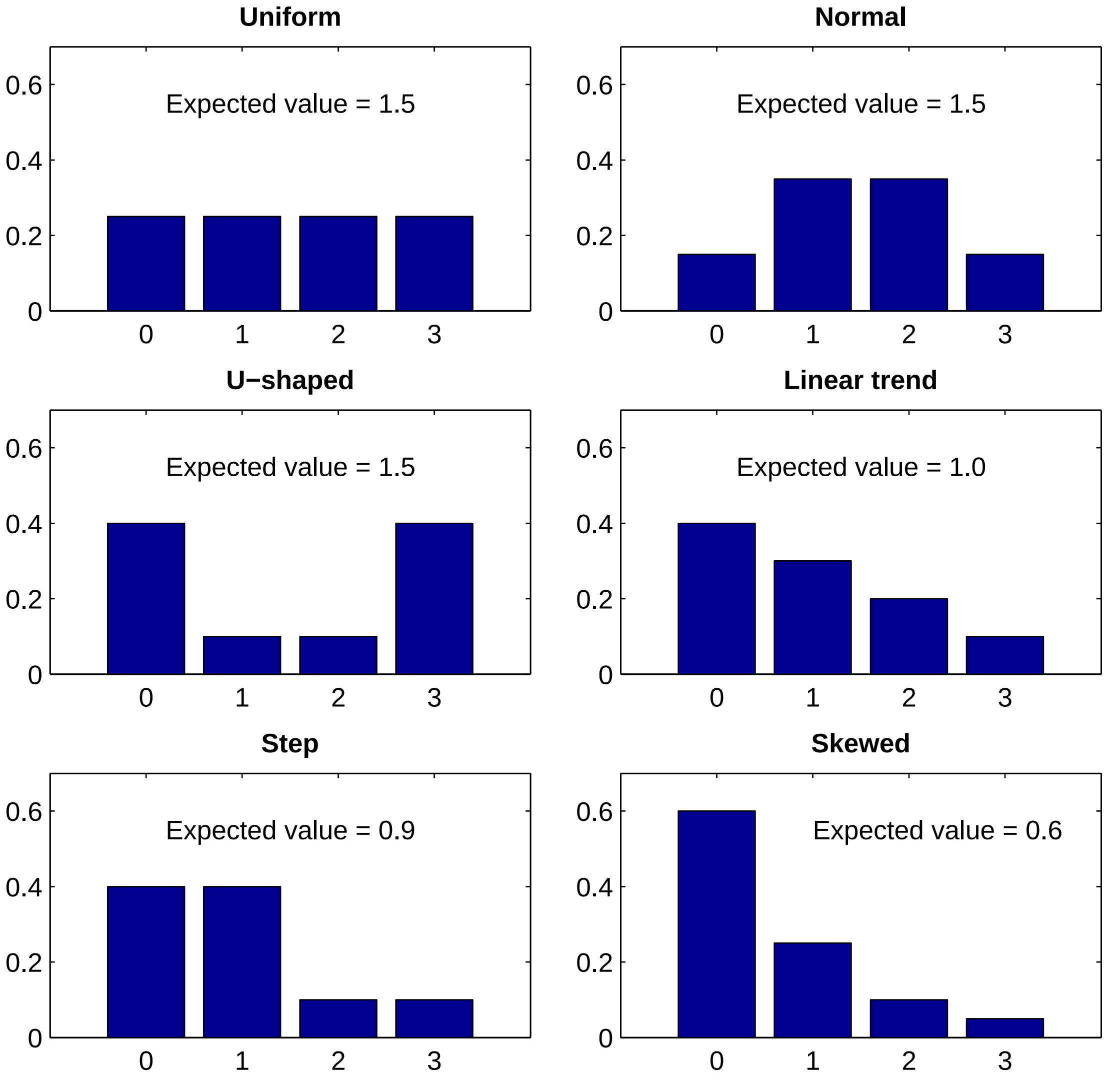


Web Figure 2: The six distributions used for the outcome scale $\{0, 1, 2, 3\}$. Plots of probabilities ($y$-axis) against outcomes ($x$-axis).

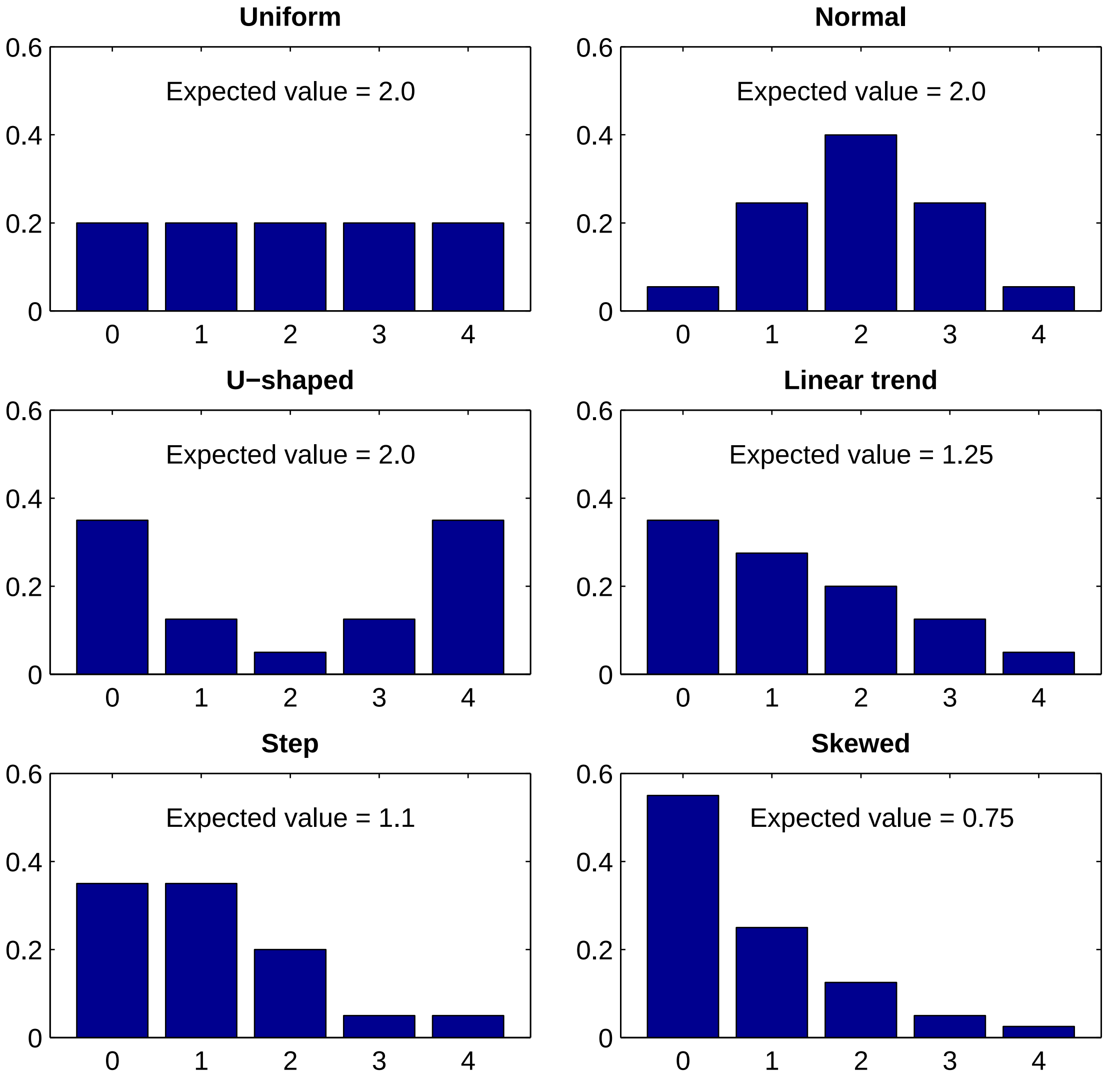


Web Figure 3: The six distributions used for the outcome scale $\{0, 1, 2, 3, 4\}$. Plots of probabilities ($y$-axis) against outcomes ($x$-axis).

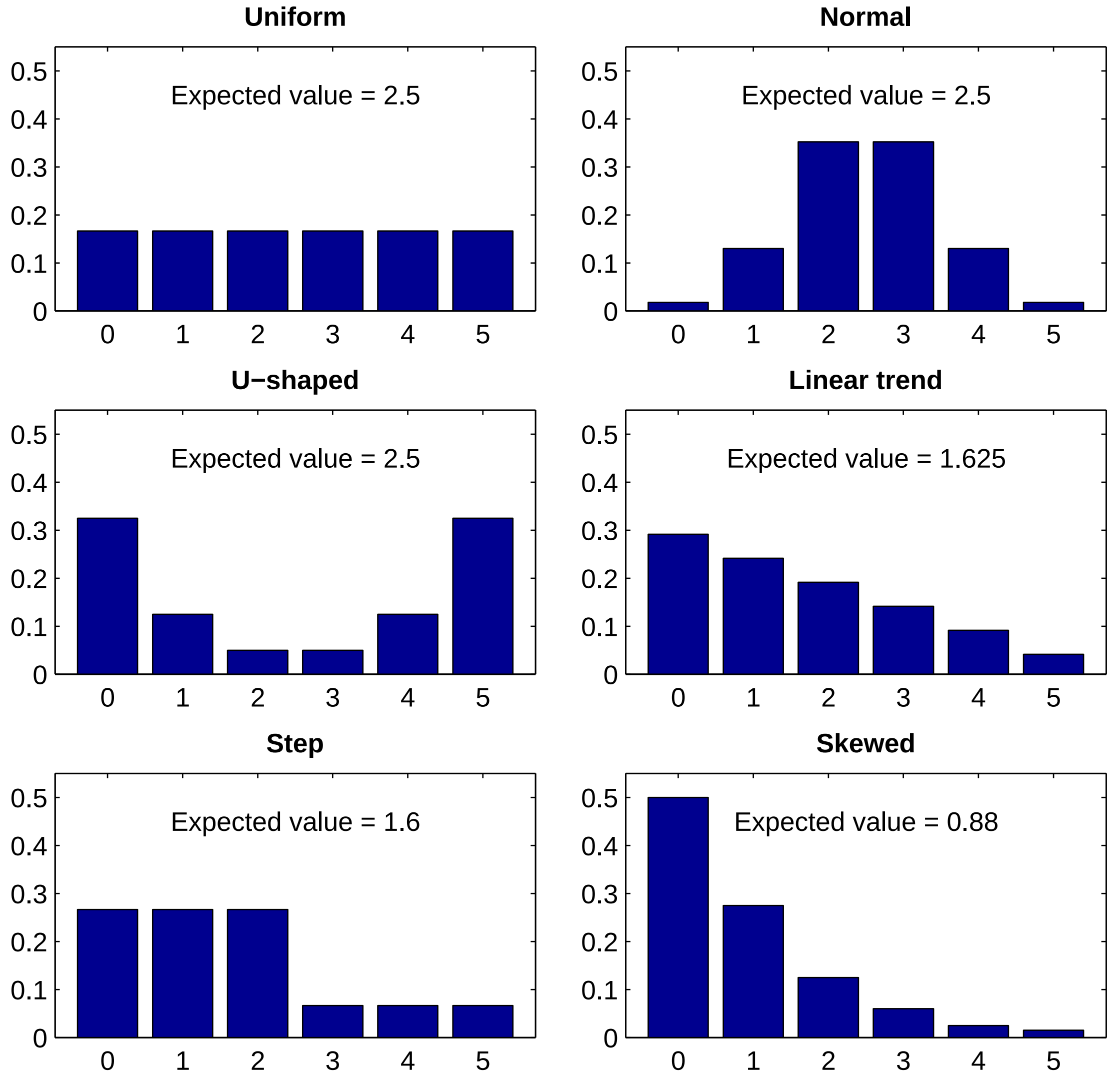


Web Figure 4: The six distributions used for the outcome scale $\{0, 1, 2, 3, 4, 5\}$. Plots of probabilities ($y$-axis) against outcomes ($x$-axis).

Web Table 1. Simulation results (hypothesis tests) for the outcome scale $\{0, 1, 2, 3, 4\}$. See description in main document.

| | Mean deviation from 5% | | | | Relative power (%) | | | |
|---|---|---|---|---|---|---|---|---|
| Sample size | T | U | WMW | BM | T | U | WMW | BM |
| $10, 10$ | **0.27** | 0.32 | *0.71* | 0.57 | **100.0** | 97.2 | *89.9* | 96.6 |
| $25, 25$ | 0.09 | **0.09** | *0.51* | 0.20 | **100.0** | 99.8 | *93.3* | 93.6 |
| $50, 50$ | 0.05 | **0.04** | *0.48* | 0.12 | **100.0** | 100.0 | 95.3 | *94.8* |
| $100, 100$ | **0.04** | 0.05 | *0.47* | 0.06 | **100.0** | 100.0 | 98.4 | *98.1* |
| $25, 10$ | *1.95* | **0.24** | 1.80 | 0.38 | **100.0** | 95.9 | *91.7* | 93.2 |
| $50, 10$ | *3.03* | **0.30** | 2.70 | 0.42 | **100.0** | 92.7 | 91.9 | *90.6* |
| $100, 50$ | *1.43* | **0.03** | 1.38 | 0.07 | **100.0** | 99.8 | 96.6 | *96.1* |
| $100, 25$ | *2.68* | **0.08** | 2.48 | 0.13 | **100.0** | 98.2 | 94.5 | *93.4* |
| $100, 10$ | *3.77* | **0.33** | 3.23 | 0.42 | **100.0** | 90.3 | 91.8 | *89.1* |

Web Table 2. Simulation results (hypothesis tests) for the outcome scale $\{0, 1, 2, 3, 4, 5\}$. See description in main document.

| | Mean deviation from 5% | | | | Relative power (%) | | | |
|---|---|---|---|---|---|---|---|---|
| Sample size | T | U | WMW | BM | T | U | WMW | BM |
| $10, 10$ | 0.33 | **0.33** | *0.81* | 0.57 | **100.0** | 97.1 | *90.4* | 95.3 |
| $25, 25$ | 0.11 | **0.10** | *0.66* | 0.21 | **100.0** | 99.7 | 93.9 | *93.8* |
| $50, 50$ | 0.07 | **0.06** | *0.61* | 0.15 | **100.0** | 100.0 | 94.3 | *93.8* |
| $100, 100$ | 0.06 | **0.05** | *0.67* | 0.07 | **100.0** | 100.0 | 95.6 | *95.1* |
| $25, 10$ | *2.44* | **0.22** | 2.08 | 0.34 | **100.0** | 96.3 | *92.1* | 93.4 |
| $50, 10$ | *3.84* | **0.29** | 3.17 | 0.38 | **100.0** | 93.8 | 92.5 | *92.0* |
| $100, 50$ | *1.81* | **0.04** | 1.68 | 0.05 | **100.0** | 99.7 | 94.5 | *93.9* |
| $100, 25$ | *3.35* | **0.09** | 2.88 | 0.12 | **100.0** | 98.1 | 94.2 | *92.8* |
| $100, 10$ | *4.89* | **0.33** | 3.84 | 0.39 | **100.0** | 91.6 | 92.7 | *90.8* |

## Outcome scale: {0, 1, 2}

$m = n = 10$

| Dist. $X$ ($m$) | Dist. $Y$ ($n$) | T | U | WMW | BM |
|---|---|---|---|---|---|
| Uniform | Uniform | 4.8 | 4.7 | 4.3 | 5.7 |
| Normal | Normal | 4.7 | 4.7 | 4.4 | 6.0 |
| U-shaped | U-shaped | 5.4 | 5.2 | 4.3 | 5.3 |
| Step1 | Step1 | 4.8 | 4.7 | 4.4 | 5.9 |
| Linear trend | Linear trend | 5.0 | 4.5 | 4.2 | 5.8 |
| Step2 | Step2 | 5.0 | 4.3 | 4.4 | 5.4 |
| Uniform | Normal | 4.8 | 4.8 | 5.2 | 6.1 |
| Uniform | U-shaped | 5.2 | 5.1 | 4.4 | 5.5 |
| Normal | U-shaped | 4.9 | 4.9 | 5.6 | 5.9 |
| Mean deviation from 5% | | **0.19** | 0.30 | 0.60 | *0.75* |

| Dist. $X$ ($m$) | Dist. $Y$ ($n$) | $E(X) - E(Y)$ | T | U | WMW | BM |
|---|---|---|---|---|---|---|
| Linear trend | Step2 | 0.03 | 5.3 | *4.7* | 4.8 | **6.1** |
| Step1 | Linear trend | 0.17 | 7.9 | 7.6 | *7.2* | **9.2** |
| Step1 | Step2 | 0.20 | 9.5 | *9.0* | 9.5 | **11.3** |
| Uniform | Step1 | 0.20 | 8.1 | 8.0 | *7.6* | **9.5** |
| Normal | Step1 | 0.20 | 9.1 | *9.0* | 9.6 | **11.3** |
| U-shaped | Step1 | 0.20 | 8.1 | 8.0 | *7.2* | **8.4** |
| Uniform | Linear trend | 0.37 | 17.0 | 16.8 | *15.5* | **18.4** |
| Normal | Linear trend | 0.37 | 21.3 | *21.2* | 22.3 | **25.0** |
| U-shaped | Linear trend | 0.37 | **15.8** | 15.5 | *13.4* | 15.1 |
| Uniform | Step2 | 0.40 | 19.0 | 18.6 | *17.9* | **20.8** |
| Normal | Step2 | 0.40 | 23.2 | *23.1* | 25.3 | **26.9** |
| U-shaped | Step2 | 0.40 | **17.9** | 17.3 | *15.4* | 17.7 |
| Sum over all distribution combinations | | | 162.1 | 158.9 | *155.7* | **179.7** |
| Relative power (per cent) | | | 90.2 | 88.4 | *86.7* | **100.0** |

Web Table 3: Estimated true significance levels (top) and power (bottom) for the two-sample T test (T), the Welch U test (U), the Wilcoxon-Mann-Whitney test (WMW), and the Brunner-Munzel test (BM). For notation and definitions, see page 1.

## Outcome scale: {0, 1, 2}

$m = n = 25$

| Dist. $X$ ($m$) | Dist. $Y$ ($n$) | T | U | WMW | BM |
|---|---|---|---|---|---|
| Uniform | Uniform | 5.1 | 5.1 | 4.8 | 5.2 |
| Normal | Normal | 5.1 | 5.0 | 4.7 | 5.4 |
| U-shaped | U-shaped | 5.0 | 5.0 | 4.8 | 5.1 |
| Step1 | Step1 | 5.0 | 5.0 | 4.8 | 5.3 |
| Linear trend | Linear trend | 5.0 | 5.0 | 4.9 | 5.2 |
| Step2 | Step2 | 5.1 | 5.1 | 5.0 | 5.1 |
| Uniform | Normal | 5.1 | 5.0 | 5.4 | 5.2 |
| Uniform | U-shaped | 5.2 | 5.2 | 5.2 | 5.3 |
| Normal | U-shaped | 5.3 | 5.2 | 6.3 | 5.3 |
| Mean deviation from 5% | | 0.10 | **0.08** | *0.33* | 0.23 |

| Dist. $X$ ($m$) | Dist. $Y$ ($n$) | $E(X) - E(Y)$ | T | U | WMW | BM |
|---|---|---|---|---|---|---|
| Linear trend | Step2 | 0.03 | *5.5* | *5.5* | 6.2 | **6.3** |
| Step1 | Linear trend | 0.17 | 12.4 | *12.3* | 12.4 | **13.1** |
| Step1 | Step2 | 0.20 | *15.3* | *15.3* | **18.8** | **18.8** |
| Uniform | Step1 | 0.20 | **14.5** | **14.5** | *13.4* | 14.3 |
| Normal | Step1 | 0.20 | 17.4 | *17.2* | 18.5 | **18.8** |
| U-shaped | Step1 | 0.20 | **13.5** | **13.5** | *12.3* | 12.4 |
| Uniform | Linear trend | 0.37 | **37.3** | **37.3** | *35.5* | 37.0 |
| Normal | Linear trend | 0.37 | 46.3 | *46.2* | **50.3** | 50.1 |
| U-shaped | Linear trend | 0.37 | **33.8** | **33.8** | *29.9* | 30.6 |
| Uniform | Step2 | 0.40 | *40.2* | *40.2* | 42.1 | **42.8** |
| Normal | Step2 | 0.40 | 48.6 | *48.5* | **56.5** | 53.6 |
| U-shaped | Step2 | 0.40 | **37.1** | **37.1** | *35.7* | 36.8 |
| Sum over all distribution combinations | | | 321.8 | *321.3* | 331.5 | **334.5** |
| Relative power (per cent) | | | 96.2 | *96.1* | 99.1 | **100.0** |

Web Table 4: Estimated true significance levels (top) and power (bottom) for the two-sample T test (T), the Welch U test (U), the Wilcoxon-Mann-Whitney test (WMW), and the Brunner-Munzel test (BM). For notation and definitions, see page 1.

## Outcome scale: {0, 1, 2}

$m = n = 50$

| Dist. $X$ ($m$) | Dist. $Y$ ($n$) | T | U | WMW | BM |
|---|---|---|---|---|---|
| Uniform | Uniform | 5.1 | 5.1 | 5.0 | 5.2 |
| Normal | Normal | 5.1 | 5.1 | 5.0 | 5.3 |
| U-shaped | U-shaped | 5.0 | 5.0 | 4.9 | 5.0 |
| Step1 | Step1 | 5.0 | 5.0 | 4.9 | 5.1 |
| Linear trend | Linear trend | 5.0 | 5.0 | 5.0 | 5.2 |
| Step2 | Step2 | 4.9 | 4.9 | 4.8 | 5.0 |
| Uniform | Normal | 5.0 | 5.0 | 5.5 | 5.1 |
| Uniform | U-shaped | 5.0 | 5.0 | 5.0 | 5.0 |
| Normal | U-shaped | 5.0 | 5.0 | 6.1 | 5.1 |
| Mean deviation from 5% | | **0.04** | 0.05 | *0.23* | 0.11 |

| Dist. $X$ ($m$) | Dist. $Y$ ($n$) | $E(X) - E(Y)$ | T | U | WMW | BM |
|---|---|---|---|---|---|---|
| Linear trend | Step2 | 0.03 | *5.5* | *5.5* | **7.3** | **7.3** |
| Step1 | Linear trend | 0.17 | *20.3* | *20.3* | 21.1 | **21.6** |
| Step1 | Step2 | 0.20 | *25.4* | *25.4* | **32.7** | 32.5 |
| Uniform | Step1 | 0.20 | **24.7** | **24.7** | *23.4* | 23.8 |
| Normal | Step1 | 0.20 | *30.1* | *30.1* | **33.0** | 32.6 |
| U-shaped | Step1 | 0.20 | **22.1** | 22.0 | 19.6 | *19.3* |
| Uniform | Linear trend | 0.37 | **64.7** | **64.7** | *62.8* | 63.5 |
| Normal | Linear trend | 0.37 | *75.0* | *75.0* | **79.7** | 79.0 |
| U-shaped | Linear trend | 0.37 | **60.3** | 60.1 | *53.9* | *53.9* |
| Uniform | Step2 | 0.40 | *68.5* | *68.5* | 71.1 | **71.2** |
| Normal | Step2 | 0.40 | *77.1* | *77.1* | **84.4** | 82.4 |
| U-shaped | Step2 | 0.40 | **63.9** | **63.9** | *62.5* | 62.9 |
| Sum over all distribution combinations | | | 537.7 | *537.3* | **551.5** | 550.0 |
| Relative power (per cent) | | | 97.5 | *97.4* | **100.0** | 99.7 |

Web Table 5: Estimated true significance levels (top) and power (bottom) for the two-sample T test (T), the Welch U test (U), the Wilcoxon-Mann-Whitney test (WMW), and the Brunner-Munzel test (BM). For notation and definitions, see page 1.

## Outcome scale: {0, 1, 2}

$m = n = 100$

| Dist. $X$ $(m)$ | Dist. $Y$ $(n)$ | T | U | WMW | BM |
|---|---|---|---|---|---|
| Uniform | Uniform | 4.8 | 4.8 | 4.7 | 4.8 |
| Normal | Normal | 5.1 | 5.1 | 4.9 | 5.1 |
| U-shaped | U-shaped | 4.9 | 4.9 | 4.9 | 4.9 |
| Step1 | Step1 | 5.0 | 5.0 | 5.1 | 5.2 |
| Linear trend | Linear trend | 5.0 | 5.0 | 5.0 | 5.1 |
| Step2 | Step2 | 5.1 | 5.1 | 5.1 | 5.2 |
| Uniform | Normal | 4.9 | 4.9 | 5.5 | 5.0 |
| Uniform | U-shaped | 5.0 | 5.0 | 5.0 | 5.0 |
| Normal | U-shaped | 5.0 | 5.0 | 6.3 | 5.0 |
| Mean deviation from 5% | | **0.07** | 0.07 | *0.27* | 0.09 |

| Dist. $X$ $(m)$ | Dist. $Y$ $(n)$ | $E(X) - E(Y)$ | T | U | WMW | BM |
|---|---|---|---|---|---|---|
| Linear trend | Step2 | 0.03 | *6.1* | *6.1* | **9.9** | 9.7 |
| Step1 | Linear trend | 0.17 | *35.5* | *35.5* | 37.4 | **37.6** |
| Step1 | Step2 | 0.20 | *44.6* | *44.6* | **57.2** | 56.5 |
| Uniform | Step1 | 0.20 | **43.6** | **43.6** | *41.4* | 41.5 |
| Normal | Step1 | 0.20 | *52.6* | *52.6* | **57.0** | 56.2 |
| U-shaped | Step1 | 0.20 | **40.2** | **40.2** | 34.9 | *34.2* |
| Uniform | Linear trend | 0.37 | **90.9** | **90.9** | *89.8* | 90.0 |
| Normal | Linear trend | 0.37 | *96.2* | *96.2* | **97.7** | 97.5 |
| U-shaped | Linear trend | 0.37 | **88.1** | **88.1** | 83.0 | *82.8* |
| Uniform | Step2 | 0.40 | *93.0* | *93.0* | **94.5** | **94.5** |
| Normal | Step2 | 0.40 | *96.9* | *96.9* | **98.8** | 98.5 |
| U-shaped | Step2 | 0.40 | **90.8** | **90.8** | *90.0* | 90.1 |
| Sum over all distribution combinations | | | 778.4 | *778.4* | **791.6** | 789.1 |
| Relative power (per cent) | | | 98.3 | *98.3* | **100.0** | 99.7 |

Web Table 6: Estimated true significance levels (top) and power (bottom) for the two-sample T test (T), the Welch U test (U), the Wilcoxon-Mann-Whitney test (WMW), and the Brunner-Munzel test (BM). For notation and definitions, see page 1.

## Outcome scale: {0, 1, 2}

$m = 25, n = 10$

| Dist. $X$ ($m$) | Dist. $Y$ ($n$) | T | U | WMW | BM |
|---|---|---|---|---|---|
| Uniform | Uniform | 5.3 | 5.3 | 4.6 | 5.5 |
| Normal | Normal | 5.2 | 5.4 | 4.7 | 5.8 |
| U-shaped | U-shaped | 5.1 | 5.6 | 4.8 | 5.5 |
| Step1 | Step1 | 5.1 | 5.4 | 4.6 | 5.6 |
| Linear trend | Linear trend | 5.1 | 5.7 | 4.7 | 5.7 |
| Step2 | Step2 | 4.8 | 6.0 | 4.5 | 5.8 |
| Uniform | Normal | 3.0 | 5.0 | 2.9 | 5.6 |
| Uniform | U-shaped | 6.5 | 5.8 | 5.9 | 5.8 |
| Normal | U-shaped | 9.7 | 5.7 | 9.9 | 5.6 |
| Normal | Uniform | 8.2 | 5.3 | 7.9 | 5.4 |
| U-shaped | Uniform | 4.2 | 5.4 | 3.9 | 5.4 |
| U-shaped | Normal | 2.5 | 5.0 | 2.9 | 5.4 |
| Mean deviation from 5% | | *1.27* | **0.29** | 1.24 | 0.42 |

| Dist. $X$ ($m$) | Dist. $Y$ ($n$) | $E(X) - E(Y)$ | T | U | WMW | BM |
|---|---|---|---|---|---|---|
| Linear trend | Step2 | 0.03 | 6.3 | 6.9 | *6.0* | **7.1** |
| Step1 | Linear trend | 0.17 | 9.1 | **11.3** | *8.8* | 10.9 |
| Step1 | Step2 | 0.20 | *12.2* | 13.4 | 13.0 | **14.0** |
| Uniform | Step1 | 0.20 | 9.3 | **11.1** | *8.3* | **11.1** |
| Normal | Step1 | 0.20 | **14.9** | *12.1* | 14.5 | 12.3 |
| U-shaped | Step1 | 0.20 | 7.7 | **10.9** | *7.1* | 10.5 |
| Uniform | Linear trend | 0.37 | 22.2 | **25.5** | *20.5* | 25.0 |
| Normal | Linear trend | 0.37 | 32.6 | *27.7* | **32.7** | 28.2 |
| U-shaped | Linear trend | 0.37 | 17.9 | **24.4** | *16.3* | 22.5 |
| Uniform | Step2 | 0.40 | 25.9 | **26.6** | *25.3* | **26.6** |
| Normal | Step2 | 0.40 | 36.6 | *28.7* | **38.2** | 29.4 |
| U-shaped | Step2 | 0.40 | 21.6 | **25.6** | *20.7* | 24.8 |
| Step2 | Linear trend | -0.03 | *4.4* | 5.2 | 4.9 | **5.7** |
| Linear trend | Step1 | -0.17 | **9.6** | *7.7* | 9.2 | 9.4 |
| Step2 | Step1 | -0.20 | 10.1 | *9.1* | 11.9 | **12.3** |
| Step1 | Uniform | -0.20 | **11.9** | *9.2* | 10.3 | 9.9 |
| Step1 | Normal | -0.20 | *10.0* | 12.1 | 10.3 | **14.5** |
| Step1 | U-shaped | -0.20 | **12.8** | 8.7 | 10.7 | *8.5* |
| Linear trend | Uniform | -0.37 | **25.6** | *19.3* | 22.9 | 21.0 |
| Linear trend | Normal | -0.37 | *26.9* | 29.0 | 29.8 | **35.3** |
| Linear trend | U-shaped | -0.37 | **25.2** | 16.7 | 20.4 | *15.8* |
| Step2 | Uniform | -0.40 | 26.0 | *22.3* | **26.3** | 25.7 |
| Step2 | Normal | -0.40 | *27.3* | 32.5 | 34.7 | **40.7** |
| Step2 | U-shaped | -0.40 | **25.5** | *19.3* | 23.1 | 19.6 |
| Sum over all distribution combinations | | | 431.6 | *415.0* | 426.1 | **440.8** |
| Relative power (per cent) | | | 97.9 | *94.1* | 96.7 | **100.0** |

Web Table 7: Estimated true significance levels (top) and power (bottom) for the two-sample T test (T), the Welch U test (U), the Wilcoxon-Mann-Whitney test (WMW), and the Brunner-Munzel test (BM). For notation and definitions, see page 1.

## Outcome scale: {0, 1, 2}

$m = 50, n = 10$

| Dist. $X$ $(m)$ | Dist. $Y$ $(n)$ | T | U | WMW | BM |
|---|---|---|---|---|---|
| Uniform | Uniform | 5.0 | 5.6 | 4.7 | 5.7 |
| Normal | Normal | 4.9 | 5.2 | 4.8 | 5.7 |
| U-shaped | U-shaped | 5.0 | 5.6 | 4.6 | 5.5 |
| Step1 | Step1 | 5.0 | 5.6 | 4.8 | 5.7 |
| Linear trend | Linear trend | 5.0 | 6.4 | 4.8 | 6.0 |
| Step2 | Step2 | 4.7 | 6.9 | 4.6 | 6.2 |
| Uniform | Normal | 2.0 | 5.2 | 2.1 | 5.8 |
| Uniform | U-shaped | 6.7 | 5.7 | 6.4 | 5.7 |
| Normal | U-shaped | 12.4 | 5.6 | 12.7 | 5.6 |
| Normal | Uniform | 10.1 | 5.6 | 10.2 | 5.8 |
| U-shaped | Uniform | 3.7 | 5.5 | 3.5 | 5.6 |
| U-shaped | Normal | 1.4 | 5.2 | 1.8 | 5.7 |
| Mean deviation from 5% | | 1.85 | **0.35** | *1.90* | 0.50 |

| Dist. $X$ $(m)$ | Dist. $Y$ $(n)$ | $E(X) - E(Y)$ | T | U | WMW | BM |
|---|---|---|---|---|---|---|
| Linear trend | Step2 | 0.03 | 6.8 | **7.8** | *6.5* | **7.8** |
| Step1 | Linear trend | 0.17 | *9.3* | **12.6** | 9.5 | 11.6 |
| Step1 | Step2 | 0.20 | *13.5* | 14.5 | 14.9 | **15.0** |
| Uniform | Step1 | 0.20 | 9.3 | 12.2 | *9.0* | **12.3** |
| Normal | Step1 | 0.20 | **17.5** | *12.5* | **17.5** | 12.8 |
| U-shaped | Step1 | 0.20 | 7.2 | **12.0** | *6.8* | 11.8 |
| Uniform | Linear trend | 0.37 | 24.5 | **28.8** | *23.9* | 28.6 |
| Normal | Linear trend | 0.37 | 39.0 | *29.8* | **39.5** | 30.3 |
| U-shaped | Linear trend | 0.37 | 19.5 | **27.8** | *18.3* | 26.9 |
| Uniform | Step2 | 0.40 | **29.5** | 29.3 | **29.5** | *29.1* |
| Normal | Step2 | 0.40 | 43.1 | *29.6* | **44.5** | 30.1 |
| U-shaped | Step2 | 0.40 | 24.2 | **28.3** | *23.4* | 27.9 |
| Step2 | Linear trend | -0.03 | *3.9* | 5.8 | 4.9 | **5.9** |
| Linear trend | Step1 | -0.17 | **10.8** | *7.8* | 10.4 | 9.7 |
| Step2 | Step1 | -0.20 | 10.6 | *9.2* | **13.8** | 13.5 |
| Step1 | Uniform | -0.20 | **13.4** | *9.7* | 11.9 | 10.2 |
| Step1 | Normal | -0.20 | *9.9* | 13.6 | 10.6 | **16.8** |
| Step1 | U-shaped | -0.20 | **14.8** | 8.9 | 12.6 | *8.5* |
| Linear trend | Uniform | -0.37 | **30.4** | *20.6* | 27.3 | 22.2 |
| Linear trend | Normal | -0.37 | *29.3* | 32.5 | 34.3 | **40.8** |
| Linear trend | U-shaped | -0.37 | **30.8** | 17.8 | 24.7 | *16.4* |
| Step2 | Uniform | -0.40 | 29.9 | *23.4* | **31.6** | 28.2 |
| Step2 | Normal | -0.40 | *29.1* | 37.3 | 41.6 | **49.6** |
| Step2 | U-shaped | -0.40 | **30.3** | *20.0* | 28.2 | 20.8 |
| Sum over all distribution combinations | | | 486.7 | *451.6* | **495.4** | 486.9 |
| Relative power (per cent) | | | 98.2 | *91.2* | **100.0** | 98.3 |

Web Table 8: Estimated true significance levels (top) and power (bottom) for the two-sample T test (T), the Welch U test (U), the Wilcoxon-Mann-Whitney test (WMW), and the Brunner-Munzel test (BM). For notation and definitions, see page 1.

## Outcome scale: {0, 1, 2}

$m = 100, n = 50$

| Dist. $X$ ($m$) | Dist. $Y$ ($n$) | T | U | WMW | BM |
|---|---|---|---|---|---|
| Uniform | Uniform | 4.9 | 4.9 | 4.8 | 5.0 |
| Normal | Normal | 5.1 | 5.1 | 5.0 | 5.3 |
| U-shaped | U-shaped | 5.1 | 5.1 | 5.0 | 5.1 |
| Step1 | Step1 | 5.1 | 5.1 | 5.0 | 5.2 |
| Linear trend | Linear trend | 5.0 | 5.0 | 4.9 | 5.0 |
| Step2 | Step2 | 5.1 | 5.2 | 5.1 | 5.2 |
| Uniform | Normal | 3.3 | 4.9 | 3.7 | 5.1 |
| Uniform | U-shaped | 5.5 | 4.8 | 5.6 | 4.8 |
| Normal | U-shaped | 8.1 | 5.0 | 9.3 | 5.1 |
| Normal | Uniform | 7.1 | 4.9 | 7.6 | 5.0 |
| U-shaped | Uniform | 4.3 | 5.0 | 4.4 | 5.1 |
| U-shaped | Normal | 2.9 | 5.1 | 3.9 | 5.1 |
| Mean deviation from 5% | | 0.87 | **0.06** | *0.89* | 0.08 |

| Dist. $X$ ($m$) | Dist. $Y$ ($n$) | $E(X) - E(Y)$ | T | U | WMW | BM |
|---|---|---|---|---|---|---|
| Linear trend | Step2 | 0.03 | 6.5 | *6.1* | **8.5** | 8.3 |
| Step1 | Linear trend | 0.17 | *25.1* | 26.1 | 26.5 | **27.2** |
| Step1 | Step2 | 0.20 | 33.3 | *32.1* | **41.6** | 39.5 |
| Uniform | Step1 | 0.20 | 30.2 | **32.2** | *29.2* | 31.3 |
| Normal | Step1 | 0.20 | 40.8 | *36.3* | **43.0** | 37.9 |
| U-shaped | Step1 | 0.20 | 26.0 | **30.1** | *23.8* | 27.0 |
| Uniform | Linear trend | 0.37 | 76.4 | **77.7** | *75.3* | 76.9 |
| Normal | Linear trend | 0.37 | 86.6 | *83.7* | **88.9** | 85.7 |
| U-shaped | Linear trend | 0.37 | 71.4 | **75.1** | *67.0* | 70.5 |
| Uniform | Step2 | 0.40 | 80.3 | *79.5* | **82.0** | 81.2 |
| Normal | Step2 | 0.40 | 88.5 | *84.4* | **91.6** | 87.1 |
| U-shaped | Step2 | 0.40 | 75.9 | **77.2** | *75.0* | 76.5 |
| Step2 | Linear trend | -0.03 | *5.2* | 5.6 | **7.9** | **7.9** |
| Linear trend | Step1 | -0.17 | 25.9 | *24.5* | **26.9** | 26.6 |
| Step2 | Step1 | -0.20 | *31.1* | 31.9 | 42.5 | **43.1** |
| Step1 | Uniform | -0.20 | **32.7** | 30.0 | 30.2 | *28.5* |
| Step1 | Normal | -0.20 | *35.9* | 39.9 | 40.7 | **44.7** |
| Step1 | U-shaped | -0.20 | **30.8** | 26.4 | 26.0 | *21.8* |
| Linear trend | Uniform | -0.37 | **77.8** | 75.4 | 75.3 | *73.8* |
| Linear trend | Normal | -0.37 | *85.9* | 88.0 | 90.6 | **91.9** |
| Linear trend | U-shaped | -0.37 | **74.2** | 69.6 | 65.9 | *61.6* |
| Step2 | Uniform | -0.40 | 80.3 | *80.2* | **83.6** | **83.6** |
| Step2 | Normal | -0.40 | *87.3* | 90.5 | 94.5 | **95.3** |
| Step2 | U-shaped | -0.40 | **77.2** | 74.8 | 75.4 | *73.6* |
| Sum over all distribution combinations | | | 1285.0 | *1277.3* | **1311.9** | 1301.5 |
| Relative power (per cent) | | | 98.0 | *97.4* | **100.0** | 99.2 |

Web Table 9: Estimated true significance levels (top) and power (bottom) for the two-sample T test (T), the Welch U test (U), the Wilcoxon-Mann-Whitney test (WMW), and the Brunner-Munzel test (BM). For notation and definitions, see page 1.

## Outcome scale: {0, 1, 2}

$m = 100, n = 25$

| Dist. $X$ ($m$) | Dist. $Y$ ($n$) | T | U | WMW | BM |
|---|---|---|---|---|---|
| Uniform | Uniform | 5.1 | 5.3 | 5.0 | 5.3 |
| Normal | Normal | 4.9 | 4.9 | 4.8 | 5.2 |
| U-shaped | U-shaped | 5.0 | 5.2 | 4.9 | 5.2 |
| Step1 | Step1 | 5.0 | 5.1 | 4.9 | 5.2 |
| Linear trend | Linear trend | 4.9 | 5.2 | 4.8 | 5.1 |
| Step2 | Step2 | 5.0 | 5.5 | 5.0 | 5.4 |
| Uniform | Normal | 2.2 | 5.0 | 2.5 | 5.2 |
| Uniform | U-shaped | 6.5 | 5.2 | 6.4 | 5.2 |
| Normal | U-shaped | 11.0 | 5.2 | 11.7 | 5.2 |
| Normal | Uniform | 9.5 | 5.3 | 9.7 | 5.3 |
| U-shaped | Uniform | 3.8 | 5.2 | 3.9 | 5.2 |
| U-shaped | Normal | 1.6 | 5.0 | 2.2 | 5.3 |
| Mean deviation from 5% | | 1.63 | **0.11** | *1.64* | 0.17 |

| Dist. $X$ ($m$) | Dist. $Y$ ($n$) | $E(X) - E(Y)$ | T | U | WMW | BM |
|---|---|---|---|---|---|---|
| Linear trend | Step2 | 0.03 | 6.9 | *6.5* | 7.8 | **7.9** |
| Step1 | Linear trend | 0.17 | *16.9* | **18.9** | 17.8 | 18.7 |
| Step1 | Step2 | 0.20 | 23.5 | *22.2* | **28.1** | 25.7 |
| Uniform | Step1 | 0.20 | 18.6 | **21.5** | *18.3* | 21.2 |
| Normal | Step1 | 0.20 | 29.7 | *22.8* | **30.6** | 23.3 |
| U-shaped | Step1 | 0.20 | 14.8 | **20.5** | *14.1* | 19.3 |
| Uniform | Linear trend | 0.37 | 53.7 | **56.7** | *53.0* | 56.1 |
| Normal | Linear trend | 0.37 | 69.0 | *60.0* | **70.7** | 61.3 |
| U-shaped | Linear trend | 0.37 | 47.0 | **55.1** | *44.8* | 52.4 |
| Uniform | Step2 | 0.40 | 59.1 | *57.3* | **60.2** | 58.1 |
| Normal | Step2 | 0.40 | 72.4 | *60.1* | **75.0** | 61.7 |
| U-shaped | Step2 | 0.40 | 53.0 | **55.8** | *52.3* | 55.3 |
| Step2 | Linear trend | -0.03 | *4.3* | 5.3 | 6.3 | **6.5** |
| Linear trend | Step1 | -0.17 | 17.7 | *14.7* | **18.0** | 16.9 |
| Step2 | Step1 | -0.20 | 19.6 | *19.3* | 27.4 | **27.6** |
| Step1 | Uniform | -0.20 | **22.7** | 18.4 | 20.6 | *17.9* |
| Step1 | Normal | -0.20 | *20.9* | 26.3 | 24.4 | **31.2** |
| Step1 | U-shaped | -0.20 | **23.5** | 16.4 | 19.5 | *14.1* |
| Linear trend | Uniform | -0.37 | **58.1** | 50.3 | 54.5 | *49.6* |
| Linear trend | Normal | -0.37 | *63.4* | 68.4 | 71.9 | **76.3** |
| Linear trend | U-shaped | -0.37 | **55.6** | 43.4 | 46.3 | *37.2* |
| Step2 | Uniform | -0.40 | 59.3 | *56.3* | **63.4** | 61.6 |
| Step2 | Normal | -0.40 | *65.2* | 74.2 | 80.7 | **85.2** |
| Step2 | U-shaped | -0.40 | **57.3** | 49.5 | 54.7 | *48.9* |
| Sum over all distribution combinations | | | 932.0 | *899.7* | **960.3** | 934.1 |
| Relative power (per cent) | | | 97.1 | *93.7* | **100.0** | 97.3 |

Web Table 10: Estimated true significance levels (top) and power (bottom) for the two-sample T test (T), the Welch U test (U), the Wilcoxon-Mann-Whitney test (WMW), and the Brunner-Munzel test (BM). For notation and definitions, see page 1.

## Outcome scale: {0, 1, 2}

$m = 100, n = 10$

| Dist. $X$ $(m)$ | Dist. $Y$ $(n)$ | T | U | WMW | BM |
|---|---|---|---|---|---|
| Uniform | Uniform | 5.0 | 5.6 | 4.9 | 5.7 |
| Normal | Normal | 5.0 | 5.1 | 4.9 | 5.6 |
| U-shaped | U-shaped | 4.9 | 5.6 | 4.7 | 5.6 |
| Step1 | Step1 | 4.9 | 5.8 | 4.8 | 5.7 |
| Linear trend | Linear trend | 4.9 | 6.4 | 4.8 | 5.9 |
| Step2 | Step2 | 4.8 | 7.4 | 4.7 | 6.5 |
| Uniform | Normal | 1.5 | 5.2 | 1.5 | 5.8 |
| Uniform | U-shaped | 7.0 | 5.6 | 6.8 | 5.6 |
| Normal | U-shaped | 14.3 | 5.6 | 14.4 | 5.5 |
| Normal | Uniform | 11.5 | 5.6 | 11.5 | 5.7 |
| U-shaped | Uniform | 3.4 | 5.7 | 3.4 | 5.9 |
| U-shaped | Normal | 0.9 | 5.3 | 1.1 | 5.8 |
| Mean deviation from 5% | | 2.25 | **0.36** | *2.27* | 0.51 |

| Dist. $X$ $(m)$ | Dist. $Y$ $(n)$ | $E(X) - E(Y)$ | T | U | WMW | BM |
|---|---|---|---|---|---|---|
| Linear trend | Step2 | 0.03 | 7.1 | **8.1** | *6.9* | **8.1** |
| Step1 | Linear trend | 0.17 | *9.6* | **13.2** | 10.1 | 12.0 |
| Step1 | Step2 | 0.20 | *14.7* | 15.2 | **16.4** | 15.7 |
| Uniform | Step1 | 0.20 | 9.7 | 12.7 | *9.5* | **12.8** |
| Normal | Step1 | 0.20 | **19.6** | *12.8* | **19.6** | 12.9 |
| U-shaped | Step1 | 0.20 | 6.9 | **12.7** | *6.7* | 12.6 |
| Uniform | Linear trend | 0.37 | 26.4 | **30.1** | *26.0* | **30.1** |
| Normal | Linear trend | 0.37 | 42.4 | *30.4* | **42.8** | *30.4* |
| U-shaped | Linear trend | 0.37 | 20.3 | **29.7** | *19.8* | 29.2 |
| Uniform | Step2 | 0.40 | **32.3** | 30.2 | **32.3** | *30.1* |
| Normal | Step2 | 0.40 | 47.7 | *30.3* | **48.4** | *30.3* |
| U-shaped | Step2 | 0.40 | 26.1 | **30.0** | *25.9* | 29.7 |
| Step2 | Linear trend | -0.03 | *3.6* | **5.9** | 4.9 | 5.8 |
| Linear trend | Step1 | -0.17 | **11.2** | *7.9* | 11.0 | 9.9 |
| Step2 | Step1 | -0.20 | 11.0 | *9.2* | **14.7** | 14.0 |
| Step1 | Uniform | -0.20 | **14.5** | *9.8* | 13.1 | 10.2 |
| Step1 | Normal | -0.20 | *9.6* | 14.4 | 10.8 | **18.6** |
| Step1 | U-shaped | -0.20 | **16.5** | 9.0 | 13.9 | *8.5* |
| Linear trend | Uniform | -0.37 | **33.3** | *21.0* | 29.9 | 22.8 |
| Linear trend | Normal | -0.37 | *31.4* | 34.9 | 38.1 | **44.2** |
| Linear trend | U-shaped | -0.37 | **34.3** | 17.8 | 27.2 | *16.2* |
| Step2 | Uniform | -0.40 | 32.7 | *24.2* | **35.1** | 29.5 |
| Step2 | Normal | -0.40 | *30.2* | 39.4 | 46.5 | **54.5** |
| Step2 | U-shaped | -0.40 | **33.4** | *20.4* | 30.7 | 21.2 |
| Sum over all distribution combinations | | | 524.5 | *469.3* | **540.3** | 509.5 |
| Relative power (per cent) | | | 97.1 | *86.9* | **100.0** | 94.3 |

Web Table 11: Estimated true significance levels (top) and power (bottom) for the two-sample T test (T), the Welch U test (U), the Wilcoxon-Mann-Whitney test (WMW), and the Brunner-Munzel test (BM). For notation and definitions, see page 1.

## Outcome scale: {0, 1, 2, 3}

$m = n = 10$

| Dist. $X$ ($m$) | Dist. $Y$ ($n$) | T | U | WMW | BM |
|---|---|---|---|---|---|
| Uniform | Uniform | 5.3 | 5.1 | 4.4 | 5.6 |
| Normal | Normal | 5.1 | 4.9 | 4.5 | 5.7 |
| U-shaped | U-shaped | 5.2 | 5.0 | 4.5 | 5.2 |
| Linear trend | Linear trend | 5.1 | 4.8 | 4.4 | 5.7 |
| Step | Step | 4.9 | 4.5 | 4.5 | 5.8 |
| Skewed | Skewed | 4.6 | 3.8 | 4.2 | 5.6 |
| Uniform | Normal | 5.3 | 5.0 | 4.7 | 5.7 |
| Uniform | U-shaped | 5.3 | 5.1 | 5.1 | 5.5 |
| Normal | U-shaped | 5.5 | 5.2 | 6.0 | 5.5 |
| Mean deviation from 5% | | **0.24** | 0.28 | 0.55 | *0.59* |

| Dist. $X$ ($m$) | Dist. $Y$ ($n$) | $E(X) - E(Y)$ | T | U | WMW | BM |
|---|---|---|---|---|---|---|
| Linear trend | Step | 0.1 | 5.6 | 5.2 | *4.9* | **6.1** |
| Step | Skewed | 0.3 | 11.1 | *10.4* | 11.5 | **13.9** |
| Linear trend | Skewed | 0.4 | 15.6 | 14.6 | *14.5* | **17.1** |
| Uniform | Linear trend | 0.5 | 17.5 | 17.0 | *15.2* | **17.8** |
| Normal | Linear trend | 0.5 | 20.8 | 20.2 | *19.6* | **22.4** |
| U-shaped | Linear trend | 0.5 | **14.2** | 13.7 | *11.3* | 11.7 |
| Uniform | Step | 0.6 | **24.2** | 23.4 | *20.6* | 23.1 |
| Normal | Step | 0.6 | 29.2 | *28.6* | 28.9 | **31.8** |
| U-shaped | Step | 0.6 | **19.0** | 18.1 | *12.9* | 13.1 |
| Uniform | Skewed | 0.9 | 47.6 | 46.4 | *44.0* | **47.9** |
| Normal | Skewed | 0.9 | 56.6 | *55.9* | 56.8 | **59.8** |
| U-shaped | Skewed | 0.9 | **37.1** | 35.5 | *28.3* | 29.2 |
| Sum over all distribution combinations | | | **298.6** | 289.0 | *268.4* | 293.8 |
| Relative power (per cent) | | | **100.0** | 96.8 | *89.9* | 98.4 |

Web Table 12: Estimated true significance levels (top) and power (bottom) for the two-sample T test (T), the Welch U test (U), the Wilcoxon-Mann-Whitney test (WMW), and the Brunner-Munzel test (BM). For notation and definitions, see page 1.

## Outcome scale: {0, 1, 2, 3}

$m = n = 25$

| Dist. $X$ ($m$) | Dist. $Y$ ($n$) | T | U | WMW | BM |
|---|---|---|---|---|---|
| Uniform | Uniform | 5.0 | 5.0 | 4.7 | 5.2 |
| Normal | Normal | 5.0 | 5.0 | 4.8 | 5.3 |
| U-shaped | U-shaped | 5.1 | 5.1 | 4.9 | 5.2 |
| Linear trend | Linear trend | 5.0 | 5.0 | 4.8 | 5.2 |
| Step | Step | 5.1 | 5.0 | 4.9 | 5.3 |
| Skewed | Skewed | 4.9 | 4.9 | 4.8 | 5.1 |
| Uniform | Normal | 5.0 | 5.0 | 5.0 | 5.2 |
| Uniform | U-shaped | 5.0 | 5.0 | 5.4 | 5.1 |
| Normal | U-shaped | 5.2 | 5.1 | 6.5 | 5.2 |
| Mean deviation from 5% | | 0.06 | **0.06** | *0.35* | 0.19 |

| Dist. $X$ ($m$) | Dist. $Y$ ($n$) | $E(X) - E(Y)$ | T | U | WMW | BM |
|---|---|---|---|---|---|---|
| Linear trend | Step | 0.1 | **6.6** | **6.6** | *6.1* | 6.4 |
| Step | Skewed | 0.3 | 21.3 | *21.2* | 25.1 | **25.9** |
| Linear trend | Skewed | 0.4 | *32.2* | *32.2* | 32.8 | **33.9** |
| Uniform | Linear trend | 0.5 | **36.9** | **36.9** | *35.0* | 36.5 |
| Normal | Linear trend | 0.5 | *43.7* | *43.7* | 45.4 | **46.6** |
| U-shaped | Linear trend | 0.5 | **30.1** | 30.0 | 23.1 | *22.2* |
| Uniform | Step | 0.6 | **51.5** | **51.5** | *48.3* | 49.1 |
| Normal | Step | 0.6 | *60.1* | *60.1* | 64.6 | **65.3** |
| U-shaped | Step | 0.6 | **42.2** | 41.9 | 27.3 | *25.8* |
| Uniform | Skewed | 0.9 | **86.5** | **86.5** | *85.4* | 86.2 |
| Normal | Skewed | 0.9 | 92.2 | *92.1* | 94.0 | **94.2** |
| U-shaped | Skewed | 0.9 | **77.2** | 77.0 | 63.4 | *63.2* |
| Sum over all distribution combinations | | | **580.6** | 579.6 | *550.5* | 555.3 |
| Relative power (per cent) | | | **100.0** | 99.8 | *94.8* | 95.6 |

Web Table 13: Estimated true significance levels (top) and power (bottom) for the two-sample T test (T), the Welch U test (U), the Wilcoxon-Mann-Whitney test (WMW), and the Brunner-Munzel test (BM). For notation and definitions, see page 1.

## Outcome scale: {0, 1, 2, 3}

$m = n = 50$

| Dist. $X$ ($m$) | Dist. $Y$ ($n$) | T | U | WMW | BM |
|---|---|---|---|---|---|
| Uniform | Uniform | 5.0 | 5.0 | 4.9 | 5.1 |
| Normal | Normal | 5.1 | 5.1 | 5.0 | 5.2 |
| U-shaped | U-shaped | 5.0 | 5.0 | 4.9 | 5.0 |
| Linear trend | Linear trend | 4.9 | 4.9 | 4.9 | 5.1 |
| Step | Step | 4.9 | 4.9 | 4.9 | 5.1 |
| Skewed | Skewed | 5.0 | 5.0 | 4.9 | 5.1 |
| Uniform | Normal | 5.1 | 5.1 | 5.3 | 5.2 |
| Uniform | U-shaped | 5.1 | 5.1 | 5.6 | 5.1 |
| Normal | U-shaped | 5.0 | 4.9 | 6.7 | 5.0 |
| Mean deviation from 5% | | **0.06** | 0.06 | *0.33* | 0.11 |

| Dist. $X$ ($m$) | Dist. $Y$ ($n$) | $E(X) - E(Y)$ | T | U | WMW | BM |
|---|---|---|---|---|---|---|
| Linear trend | Step | 0.1 | **8.1** | 8.0 | *7.3* | 7.5 |
| Step | Skewed | 0.3 | 38.0 | *37.9* | 45.5 | **45.8** |
| Linear trend | Skewed | 0.4 | 56.4 | *56.3* | 58.2 | **58.7** |
| Uniform | Linear trend | 0.5 | **64.4** | **64.4** | *62.2* | 62.9 |
| Normal | Linear trend | 0.5 | *72.5* | *72.5* | 75.4 | **75.5** |
| U-shaped | Linear trend | 0.5 | **54.2** | 54.1 | 41.1 | *39.4* |
| Uniform | Step | 0.6 | **81.3** | **81.3** | *78.5* | 78.6 |
| Normal | Step | 0.6 | 88.1 | *88.0* | **91.7** | **91.7** |
| U-shaped | Step | 0.6 | **71.4** | 71.3 | 48.3 | *45.9* |
| Uniform | Skewed | 0.9 | **99.2** | **99.2** | *99.0* | 99.1 |
| Normal | Skewed | 0.9 | *99.8* | *99.8* | **99.9** | **99.9** |
| U-shaped | Skewed | 0.9 | **97.2** | **97.2** | 90.8 | *90.4* |
| Sum over all distribution combinations | | | **830.4** | 830.0 | 798.0 | *795.4* |
| Relative power (per cent) | | | **100.0** | 99.9 | 96.1 | *95.8* |

Web Table 14: Estimated true significance levels (top) and power (bottom) for the two-sample T test (T), the Welch U test (U), the Wilcoxon-Mann-Whitney test (WMW), and the Brunner-Munzel test (BM). For notation and definitions, see page 1.

## Outcome scale: {0, 1, 2, 3}

$m = n = 100$

| Dist. $X$ ($m$) | Dist. $Y$ ($n$) | T | U | WMW | BM |
|---|---|---|---|---|---|
| Uniform | Uniform | 5.0 | 5.0 | 5.0 | 5.1 |
| Normal | Normal | 5.0 | 5.0 | 5.0 | 5.1 |
| U-shaped | U-shaped | 4.8 | 4.8 | 4.8 | 4.9 |
| Linear trend | Linear trend | 5.1 | 5.1 | 5.1 | 5.1 |
| Step | Step | 5.1 | 5.1 | 5.0 | 5.1 |
| Skewed | Skewed | 5.1 | 5.1 | 5.0 | 5.1 |
| Uniform | Normal | 5.0 | 5.0 | 5.2 | 5.0 |
| Uniform | U-shaped | 5.1 | 5.1 | 5.7 | 5.1 |
| Normal | U-shaped | 5.0 | 5.0 | 6.7 | 5.0 |
| Mean deviation from 5% | | 0.07 | **0.07** | *0.33* | 0.10 |

| Dist. $X$ ($m$) | Dist. $Y$ ($n$) | $E(X) - E(Y)$ | T | U | WMW | BM |
|---|---|---|---|---|---|---|
| Linear trend | Step | 0.1 | **11.2** | **11.2** | *9.8* | *9.8* |
| Step | Skewed | 0.3 | *65.0* | *65.0* | 74.7 | **74.8** |
| Linear trend | Skewed | 0.4 | 85.3 | *85.2* | 87.0 | **87.1** |
| Uniform | Linear trend | 0.5 | **91.0** | 90.9 | *89.5* | 89.7 |
| Normal | Linear trend | 0.5 | *95.2* | *95.2* | **96.4** | **96.4** |
| U-shaped | Linear trend | 0.5 | **83.4** | **83.4** | 68.3 | *66.4* |
| Uniform | Step | 0.6 | **98.0** | **98.0** | 97.3 | *97.2* |
| Normal | Step | 0.6 | *99.3* | *99.3* | **99.7** | **99.7** |
| U-shaped | Step | 0.6 | **94.7** | **94.7** | 76.9 | *74.7* |
| Uniform | Skewed | 0.9 | 100.0 | 100.0 | 100.0 | 100.0 |
| Normal | Skewed | 0.9 | 100.0 | 100.0 | 100.0 | 100.0 |
| U-shaped | Skewed | 0.9 | **100.0** | **100.0** | 99.7 | *99.6* |
| Sum over all distribution combinations | | | **1023.0** | 1023.0 | 999.3 | *995.3* |
| Relative power (per cent) | | | **100.0** | 100.0 | 97.7 | *97.3* |

Web Table 15: Estimated true significance levels (top) and power (bottom) for the two-sample T test (T), the Welch U test (U), the Wilcoxon-Mann-Whitney test (WMW), and the Brunner-Munzel test (BM). For notation and definitions, see page 1.

## Outcome scale: {0, 1, 2, 3}

$m = 25, n = 10$

| Dist. $X$ ($m$) | Dist. $Y$ ($n$) | T | U | WMW | BM |
|---|---|---|---|---|---|
| Uniform | Uniform | 5.1 | 5.3 | 4.6 | 5.6 |
| Normal | Normal | 5.2 | 5.3 | 4.7 | 5.8 |
| U-shaped | U-shaped | 5.1 | 5.5 | 4.7 | 5.5 |
| Linear trend | Linear trend | 5.0 | 5.6 | 4.6 | 5.7 |
| Step | Step | 5.0 | 5.8 | 4.6 | 5.6 |
| Skewed | Skewed | 4.7 | 6.5 | 4.6 | 6.0 |
| Uniform | Normal | 3.4 | 5.1 | 3.2 | 5.6 |
| Uniform | U-shaped | 7.3 | 5.5 | 7.4 | 5.6 |
| Normal | U-shaped | 10.1 | 5.7 | 10.4 | 5.7 |
| Normal | Uniform | 7.3 | 5.5 | 6.8 | 5.7 |
| U-shaped | Uniform | 3.3 | 5.3 | 3.4 | 5.5 |
| U-shaped | Normal | 2.1 | 5.1 | 2.8 | 5.3 |
| Mean deviation from 5% | | *1.35* | **0.27** | 1.34 | 0.44 |

| Dist. $X$ ($m$) | Dist. $Y$ ($n$) | $E(X) - E(Y)$ | T | U | WMW | BM |
|---|---|---|---|---|---|---|
| Linear trend | Step | 0.1 | 5.2 | **8.1** | *4.8* | 6.9 |
| Step | Skewed | 0.3 | *12.7* | **19.7** | 15.5 | 18.4 |
| Linear trend | Skewed | 0.4 | *17.8* | **25.7** | 18.5 | 23.7 |
| Uniform | Linear trend | 0.5 | 21.5 | **24.8** | *20.5* | 24.5 |
| Normal | Linear trend | 0.5 | **29.4** | *26.8* | 28.8 | 27.1 |
| U-shaped | Linear trend | 0.5 | 14.2 | **22.3** | *11.9* | 18.7 |
| Uniform | Step | 0.6 | 30.2 | **36.0** | *28.3* | 34.0 |
| Normal | Step | 0.6 | 40.3 | *38.8* | **41.4** | 40.8 |
| U-shaped | Step | 0.6 | 20.0 | **32.0** | *14.2* | 21.7 |
| Uniform | Skewed | 0.9 | 61.2 | **65.9** | *60.4* | 65.1 |
| Normal | Skewed | 0.9 | 72.6 | *69.5* | **73.5** | 70.6 |
| U-shaped | Skewed | 0.9 | 45.7 | **60.3** | *38.0* | 51.0 |
| Step | Linear trend | -0.1 | **6.8** | *5.4* | 6.1 | 6.0 |
| Skewed | Step | -0.3 | 15.6 | *9.2* | **16.4** | 15.3 |
| Skewed | Linear trend | -0.4 | **22.8** | *14.2* | 21.5 | 18.6 |
| Linear trend | Uniform | -0.5 | **25.3** | *19.6* | 22.1 | 21.2 |
| Linear trend | Normal | -0.5 | 26.5 | *26.3* | 27.0 | **31.6** |
| Linear trend | U-shaped | -0.5 | **24.5** | 14.8 | 17.8 | *11.9* |
| Step | Uniform | -0.6 | **36.1** | *26.7* | 31.1 | 28.0 |
| Step | Normal | -0.6 | 39.1 | *36.3* | 41.4 | **44.0** |
| Step | U-shaped | -0.6 | **32.7** | 19.2 | 20.4 | *13.4* |
| Skewed | Uniform | -0.9 | **66.7** | *53.8* | 62.3 | 56.9 |
| Skewed | Normal | -0.9 | 73.2 | *69.0* | 76.3 | **76.4** |
| Skewed | U-shaped | -0.9 | **59.2** | 38.3 | 42.3 | *30.5* |
| Sum over all distribution combinations | | | **799.2** | 762.5 | *740.5* | 756.4 |
| Relative power (per cent) | | | **100.0** | 95.4 | *92.7* | 94.6 |

Web Table 16: Estimated true significance levels (top) and power (bottom) for the two-sample T test (T), the Welch U test (U), the Wilcoxon-Mann-Whitney test (WMW), and the Brunner-Munzel test (BM). For notation and definitions, see page 1.

## Outcome scale: {0, 1, 2, 3}

$m = 50, n = 10$

| Dist. $X$ ($m$) | Dist. $Y$ ($n$) | T | U | WMW | BM |
|---|---|---|---|---|---|
| Uniform | Uniform | 5.1 | 5.6 | 4.9 | 5.8 |
| Normal | Normal | 5.0 | 5.4 | 4.8 | 5.7 |
| U-shaped | U-shaped | 5.0 | 5.6 | 4.7 | 5.6 |
| Linear trend | Linear trend | 4.9 | 6.0 | 4.7 | 5.7 |
| Step | Step | 4.8 | 6.7 | 4.7 | 5.7 |
| Skewed | Skewed | 4.7 | 8.0 | 4.7 | 6.3 |
| Uniform | Normal | 2.6 | 5.4 | 2.7 | 5.8 |
| Uniform | U-shaped | 8.7 | 5.7 | 8.8 | 5.6 |
| Normal | U-shaped | 13.4 | 5.7 | 13.1 | 5.6 |
| Normal | Uniform | 8.7 | 5.6 | 8.0 | 5.7 |
| U-shaped | Uniform | 2.5 | 5.5 | 2.4 | 5.7 |
| U-shaped | Normal | 1.1 | 5.3 | 1.4 | 5.7 |
| Mean deviation from 5% | | *2.05* | **0.39** | 2.00 | 0.51 |

| Dist. $X$ ($m$) | Dist. $Y$ ($n$) | $E(X) - E(Y)$ | T | U | WMW | BM |
|---|---|---|---|---|---|---|
| Linear trend | Step | 0.1 | 4.7 | **9.5** | *4.5* | 7.3 |
| Step | Skewed | 0.3 | *13.8* | **23.3** | 17.9 | 20.4 |
| Linear trend | Skewed | 0.4 | *19.9* | **30.8** | 21.7 | 27.4 |
| Uniform | Linear trend | 0.5 | 24.1 | **27.9** | *23.6* | 27.6 |
| Normal | Linear trend | 0.5 | **34.8** | *28.9* | 33.9 | 29.1 |
| U-shaped | Linear trend | 0.5 | 14.1 | **26.6** | *12.2* | 23.2 |
| Uniform | Step | 0.6 | 33.9 | **40.8** | *33.0* | 39.3 |
| Normal | Step | 0.6 | 46.5 | *41.7* | **47.8** | 43.9 |
| U-shaped | Step | 0.6 | 20.6 | **38.5** | *14.8* | 27.7 |
| Uniform | Skewed | 0.9 | 68.9 | **71.8** | *68.7* | 71.3 |
| Normal | Skewed | 0.9 | 80.1 | *73.2* | **80.4** | 73.9 |
| U-shaped | Skewed | 0.9 | 51.5 | **68.9** | *45.0* | 62.0 |
| Step | Linear trend | -0.1 | **7.2** | *5.6* | 6.8 | 6.1 |
| Skewed | Step | -0.3 | 18.1 | *8.8* | **19.3** | 16.1 |
| Skewed | Linear trend | -0.4 | **27.3** | *14.2* | 25.9 | 19.5 |
| Linear trend | Uniform | -0.5 | **30.1** | *20.6* | 26.3 | 22.6 |
| Linear trend | Normal | -0.5 | 29.3 | *28.8* | 31.3 | **36.3** |
| Linear trend | U-shaped | -0.5 | **30.7** | 14.9 | 21.2 | *11.8* |
| Step | Uniform | -0.6 | **42.4** | *28.1* | 36.5 | 29.7 |
| Step | Normal | -0.6 | 44.1 | *39.4* | 48.4 | **48.6** |
| Step | U-shaped | -0.6 | **40.6** | 19.6 | 24.5 | *13.4* |
| Skewed | Uniform | -0.9 | **75.5** | *56.5* | 70.8 | 59.9 |
| Skewed | Normal | -0.9 | 81.2 | *73.8* | **84.8** | 81.6 |
| Skewed | U-shaped | -0.9 | **69.0** | 39.3 | 49.2 | *30.9* |
| Sum over all distribution combinations | | | **908.5** | 831.4 | 848.5 | *829.6* |
| Relative power (per cent) | | | **100.0** | 91.5 | 93.4 | *91.3* |

Web Table 17: Estimated true significance levels (top) and power (bottom) for the two-sample T test (T), the Welch U test (U), the Wilcoxon-Mann-Whitney test (WMW), and the Brunner-Munzel test (BM). For notation and definitions, see page 1.

## Outcome scale: {0, 1, 2, 3}

$m = 100, n = 50$

| Dist. $X$ $(m)$ | Dist. $Y$ $(n)$ | T | U | WMW | BM |
|---|---|---|---|---|---|
| Uniform | Uniform | 5.1 | 5.1 | 5.0 | 5.2 |
| Normal | Normal | 4.9 | 5.0 | 4.9 | 5.1 |
| U-shaped | U-shaped | 4.9 | 4.9 | 4.8 | 4.9 |
| Linear trend | Linear trend | 5.1 | 5.1 | 5.0 | 5.1 |
| Step | Step | 5.0 | 5.1 | 4.9 | 5.1 |
| Skewed | Skewed | 5.0 | 5.2 | 4.9 | 5.1 |
| Uniform | Normal | 3.7 | 5.0 | 3.9 | 5.0 |
| Uniform | U-shaped | 6.6 | 5.1 | 7.3 | 5.0 |
| Normal | U-shaped | 8.4 | 4.9 | 9.9 | 5.0 |
| Normal | Uniform | 6.6 | 5.0 | 6.7 | 5.1 |
| U-shaped | Uniform | 3.7 | 5.1 | 4.0 | 5.1 |
| U-shaped | Normal | 2.6 | 4.9 | 3.8 | 5.0 |
| Mean deviation from 5% | | 1.00 | **0.04** | *1.03* | 0.06 |

| Dist. $X$ $(m)$ | Dist. $Y$ $(n)$ | $E(X) - E(Y)$ | T | U | WMW | BM |
|---|---|---|---|---|---|---|
| Linear trend | Step | 0.1 | 8.6 | **10.2** | *7.8* | 8.8 |
| Step | Skewed | 0.3 | *47.3* | 49.5 | **56.4** | **56.4** |
| Linear trend | Skewed | 0.4 | *68.0* | 70.2 | 70.6 | **72.2** |
| Uniform | Linear trend | 0.5 | 76.2 | **77.6** | *74.9* | 76.5 |
| Normal | Linear trend | 0.5 | 84.2 | *82.2* | **85.5** | 83.6 |
| U-shaped | Linear trend | 0.5 | 64.2 | **71.0** | *52.1* | 57.4 |
| Uniform | Step | 0.6 | 90.6 | **91.6** | *89.3* | 90.4 |
| Normal | Step | 0.6 | 95.1 | *94.2* | **96.8** | 96.3 |
| U-shaped | Step | 0.6 | 81.6 | **86.7** | *61.2* | 66.0 |
| Uniform | Skewed | 0.9 | 99.9 | 99.9 | 99.9 | 99.9 |
| Normal | Skewed | 0.9 | 100.0 | 100.0 | 100.0 | 100.0 |
| U-shaped | Skewed | 0.9 | 99.5 | **99.7** | *97.7* | 98.4 |
| Step | Linear trend | -0.1 | **9.9** | 8.5 | 8.9 | *8.2* |
| Skewed | Step | -0.3 | 49.0 | *46.0* | **58.0** | 57.4 |
| Skewed | Linear trend | -0.4 | 70.1 | *67.0* | **71.8** | 70.1 |
| Linear trend | Uniform | -0.5 | **77.6** | 75.2 | 74.6 | *73.2* |
| Linear trend | Normal | -0.5 | *83.7* | 85.1 | 87.2 | **88.6** |
| Linear trend | U-shaped | -0.5 | **69.5** | 61.9 | 51.3 | *43.0* |
| Step | Uniform | -0.6 | **91.2** | 89.5 | 88.2 | *86.4* |
| Step | Normal | -0.6 | *95.2* | 95.5 | **97.4** | **97.4** |
| Step | U-shaped | -0.6 | **84.7** | 78.8 | 59.4 | *50.1* |
| Skewed | Uniform | -0.9 | **99.9** | *99.8* | *99.8* | *99.8* |
| Skewed | Normal | -0.9 | 100.0 | 100.0 | 100.0 | 100.0 |
| Skewed | U-shaped | -0.9 | **99.4** | 98.9 | 95.8 | *93.9* |
| Sum over all distribution combinations | | | **1845.2** | 1839.0 | 1784.7 | *1773.6* |
| Relative power (per cent) | | | **100.0** | 99.7 | 96.7 | *96.1* |

Web Table 18: Estimated true significance levels (top) and power (bottom) for the two-sample T test (T), the Welch U test (U), the Wilcoxon-Mann-Whitney test (WMW), and the Brunner-Munzel test (BM). For notation and definitions, see page 1.

## Outcome scale: {0, 1, 2, 3}

$m = 100, n = 25$

| Dist. $X$ ($m$) | Dist. $Y$ ($n$) | T | U | WMW | BM |
|---|---|---|---|---|---|
| Uniform | Uniform | 5.0 | 5.2 | 4.9 | 5.2 |
| Normal | Normal | 5.0 | 5.1 | 5.0 | 5.3 |
| U-shaped | U-shaped | 5.0 | 5.1 | 5.0 | 5.2 |
| Linear trend | Linear trend | 5.1 | 5.3 | 5.0 | 5.3 |
| Step | Step | 4.9 | 5.4 | 4.9 | 5.2 |
| Skewed | Skewed | 4.9 | 5.9 | 4.9 | 5.4 |
| Uniform | Normal | 2.6 | 4.9 | 2.7 | 5.0 |
| Uniform | U-shaped | 8.1 | 5.2 | 8.5 | 5.2 |
| Normal | U-shaped | 11.8 | 5.1 | 12.3 | 5.1 |
| Normal | Uniform | 8.1 | 5.1 | 7.7 | 5.1 |
| U-shaped | Uniform | 2.7 | 5.1 | 2.8 | 5.2 |
| U-shaped | Normal | 1.4 | 5.2 | 1.9 | 5.2 |
| Mean deviation from 5% | | *1.78* | **0.09** | 1.77 | 0.12 |

| Dist. $X$ ($m$) | Dist. $Y$ ($n$) | $E(X) - E(Y)$ | T | U | WMW | BM |
|---|---|---|---|---|---|---|
| Linear trend | Step | 0.1 | 6.4 | **9.4** | *5.9* | 7.7 |
| Step | Skewed | 0.3 | *29.8* | 35.1 | 37.2 | **37.3** |
| Linear trend | Skewed | 0.4 | *45.2* | 51.1 | 48.0 | **51.3** |
| Uniform | Linear trend | 0.5 | 53.1 | **56.2** | *52.4* | 55.4 |
| Normal | Linear trend | 0.5 | 65.3 | *58.9* | **65.4** | 59.6 |
| U-shaped | Linear trend | 0.5 | 38.3 | **52.3** | *32.1* | 44.1 |
| Uniform | Step | 0.6 | 70.7 | **74.0** | *69.5* | 72.7 |
| Normal | Step | 0.6 | 80.8 | *76.5* | **83.5** | 79.8 |
| U-shaped | Step | 0.6 | 55.6 | **70.1** | *39.2* | 52.3 |
| Uniform | Skewed | 0.9 | *97.3* | **97.5** | *97.3* | **97.5** |
| Normal | Skewed | 0.9 | 99.0 | *98.2* | **99.2** | 98.6 |
| U-shaped | Skewed | 0.9 | 93.0 | **96.5** | *87.5* | 92.9 |
| Step | Linear trend | -0.1 | **8.6** | *6.4* | 8.0 | 6.7 |
| Skewed | Step | -0.3 | 33.3 | *25.6* | **39.1** | 36.6 |
| Skewed | Linear trend | -0.4 | 50.9 | *40.9* | **51.2** | 45.9 |
| Linear trend | Uniform | -0.5 | **57.4** | 50.1 | 53.1 | *49.1* |
| Linear trend | Normal | -0.5 | *61.5* | 63.8 | 66.9 | **70.5** |
| Linear trend | U-shaped | -0.5 | **52.7** | 36.9 | 36.2 | *24.5* |
| Step | Uniform | -0.6 | **74.7** | 66.0 | 68.7 | *62.2* |
| Step | Normal | -0.6 | 80.4 | *80.3* | **86.0** | 85.9 |
| Step | U-shaped | -0.6 | **68.5** | 50.8 | 42.4 | *28.6* |
| Skewed | Uniform | -0.9 | **97.8** | 95.9 | 96.9 | *95.3* |
| Skewed | Normal | -0.9 | 99.2 | *99.1* | **99.6** | **99.6** |
| Skewed | U-shaped | -0.9 | **94.8** | 86.7 | 81.8 | *71.0* |
| Sum over all distribution combinations | | | **1514.3** | 1478.3 | 1447.1 | *1424.9* |
| Relative power (per cent) | | | **100.0** | 97.6 | 95.6 | *94.1* |

Web Table 19: Estimated true significance levels (top) and power (bottom) for the two-sample T test (T), the Welch U test (U), the Wilcoxon-Mann-Whitney test (WMW), and the Brunner-Munzel test (BM). For notation and definitions, see page 1.

## Outcome scale: {0, 1, 2, 3}

$m = 100, n = 10$

| Dist. $X$ $(m)$ | Dist. $Y$ $(n)$ | T | U | WMW | BM |
|---|---|---|---|---|---|
| Uniform | Uniform | 4.9 | 5.6 | 4.8 | 5.7 |
| Normal | Normal | 5.1 | 5.5 | 5.0 | 5.8 |
| U-shaped | U-shaped | 4.8 | 5.5 | 4.7 | 5.6 |
| Linear trend | Linear trend | 5.0 | 6.3 | 4.9 | 5.7 |
| Step | Step | 4.9 | 7.2 | 5.0 | 5.8 |
| Skewed | Skewed | 4.5 | 8.8 | 4.6 | 6.2 |
| Uniform | Normal | 2.1 | 5.4 | 2.1 | 5.7 |
| Uniform | U-shaped | 9.7 | 5.7 | 9.7 | 5.5 |
| Normal | U-shaped | 15.7 | 5.5 | 14.7 | 5.4 |
| Normal | Uniform | 9.6 | 5.7 | 8.9 | 5.8 |
| U-shaped | Uniform | 2.0 | 5.6 | 1.9 | 5.8 |
| U-shaped | Normal | 0.6 | 5.4 | 0.6 | 5.7 |
| Mean deviation from 5% | | *2.55* | **0.41** | 2.42 | 0.50 |

| Dist. $X$ $(m)$ | Dist. $Y$ $(n)$ | $E(X) - E(Y)$ | T | U | WMW | BM |
|---|---|---|---|---|---|---|
| Linear trend | Step | 0.1 | 4.6 | **10.5** | *4.4* | 7.5 |
| Step | Skewed | 0.3 | *14.2* | **24.6** | 19.2 | 20.9 |
| Linear trend | Skewed | 0.4 | *20.6* | **32.3** | 23.1 | 28.6 |
| Uniform | Linear trend | 0.5 | 25.8 | **29.5** | *25.6* | 29.3 |
| Normal | Linear trend | 0.5 | **38.1** | *29.7* | 36.9 | *29.7* |
| U-shaped | Linear trend | 0.5 | 14.1 | **28.9** | *12.2* | 26.5 |
| Uniform | Step | 0.6 | 36.5 | **42.9** | *36.1* | 42.2 |
| Normal | Step | 0.6 | 50.9 | *43.2* | **52.2** | 45.8 |
| U-shaped | Step | 0.6 | 21.0 | **41.8** | *15.0* | 32.1 |
| Uniform | Skewed | 0.9 | *73.1* | **74.1** | 73.2 | 73.8 |
| Normal | Skewed | 0.9 | **83.9** | *74.5* | 83.5 | 74.8 |
| U-shaped | Skewed | 0.9 | 55.7 | **72.9** | *49.9* | 68.3 |
| Step | Linear trend | -0.1 | **7.6** | *5.7* | 7.3 | 6.2 |
| Skewed | Step | -0.3 | 19.7 | *8.7* | **21.4** | 16.6 |
| Skewed | Linear trend | -0.4 | **29.7** | *14.2* | 28.6 | 19.6 |
| Linear trend | Uniform | -0.5 | **33.3** | *21.0* | 29.0 | 23.3 |
| Linear trend | Normal | -0.5 | 31.5 | *30.1* | 34.4 | **39.0** |
| Linear trend | U-shaped | -0.5 | **34.5** | 14.9 | 23.2 | *11.7* |
| Step | Uniform | -0.6 | **46.9** | *28.7* | 40.2 | 30.5 |
| Step | Normal | -0.6 | 47.4 | *40.9* | **53.0** | 51.3 |
| Step | U-shaped | -0.6 | **45.9** | 19.7 | 27.1 | *13.5* |
| Skewed | Uniform | -0.9 | **80.0** | *57.5* | 74.8 | 60.8 |
| Skewed | Normal | -0.9 | 85.3 | *75.9* | **88.8** | 83.7 |
| Skewed | U-shaped | -0.9 | **74.6** | 39.9 | 53.1 | *31.9* |
| Sum over all distribution combinations | | | **975.0** | *862.1* | 912.1 | 867.6 |
| Relative power (per cent) | | | **100.0** | *88.4* | 93.6 | 89.0 |

Web Table 20: Estimated true significance levels (top) and power (bottom) for the two-sample T test (T), the Welch U test (U), the Wilcoxon-Mann-Whitney test (WMW), and the Brunner-Munzel test (BM). For notation and definitions, see page 1.

## Outcome scale: {0, 1, 2, 3, 4}

$m = n = 10$

| Dist. $X$ ($m$) | Dist. $Y$ ($n$) | T | U | WMW | BM |
|---|---|---|---|---|---|
| Uniform | Uniform | 5.2 | 5.1 | 4.4 | 5.6 |
| Normal | Normal | 5.0 | 4.8 | 4.2 | 5.7 |
| U-shaped | U-shaped | 5.1 | 5.0 | 4.3 | 5.2 |
| Linear trend | Linear trend | 5.1 | 4.8 | 4.4 | 5.6 |
| Step | Step | 4.8 | 4.4 | 4.3 | 5.7 |
| Skewed | Skewed | 4.7 | 4.0 | 4.3 | 5.6 |
| Uniform | Normal | 5.5 | 5.2 | 5.1 | 5.7 |
| Uniform | U-shaped | 5.3 | 5.2 | 5.0 | 5.5 |
| Normal | U-shaped | 5.8 | 5.4 | 7.2 | 5.5 |
| Mean deviation from 5% | | **0.27** | 0.32 | *0.71* | 0.57 |

| Dist. $X$ ($m$) | Dist. $Y$ ($n$) | $E(X) - E(Y)$ | T | U | WMW | BM |
|---|---|---|---|---|---|---|
| Linear trend | Step | 0.15 | 5.8 | 5.4 | *4.9* | **6.2** |
| Step | Skewed | 0.35 | 11.7 | *10.9* | 12.2 | **14.3** |
| Linear trend | Skewed | 0.50 | 16.4 | 15.4 | *15.3* | **17.7** |
| Uniform | Linear trend | 0.75 | 22.8 | 22.3 | *19.9* | **23.1** |
| Normal | Linear trend | 0.75 | 32.4 | 31.7 | *31.3* | **33.9** |
| U-shaped | Linear trend | 0.75 | **17.8** | 17.0 | *13.2* | 13.8 |
| Uniform | Step | 0.90 | **32.8** | 31.9 | *28.0* | 31.0 |
| Normal | Step | 0.90 | 47.1 | *46.2* | 47.5 | **50.7** |
| U-shaped | Step | 0.90 | **24.6** | 23.3 | *15.8* | 15.9 |
| Uniform | Skewed | 1.25 | **56.5** | 55.4 | *51.9* | 56.0 |
| Normal | Skewed | 1.25 | 73.5 | *72.8* | 74.7 | **75.9** |
| U-shaped | Skewed | 1.25 | **43.8** | 41.8 | *32.0* | 33.5 |
| Sum over all distribution combinations | | | **385.2** | 374.3 | *346.4* | 371.9 |
| Relative power (per cent) | | | **100.0** | 97.2 | *89.9* | 96.6 |

Web Table 21: Estimated true significance levels (top) and power (bottom) for the two-sample T test (T), the Welch U test (U), the Wilcoxon-Mann-Whitney test (WMW), and the Brunner-Munzel test (BM). For notation and definitions, see page 1.

## Outcome scale: {0, 1, 2, 3, 4}

$m = n = 25$

| Dist. $X$ ($m$) | Dist. $Y$ ($n$) | T | U | WMW | BM |
|---|---|---|---|---|---|
| Uniform | Uniform | 5.0 | 5.0 | 4.7 | 5.1 |
| Normal | Normal | 5.0 | 5.0 | 4.7 | 5.3 |
| U-shaped | U-shaped | 5.3 | 5.2 | 5.0 | 5.3 |
| Linear trend | Linear trend | 4.9 | 4.9 | 4.7 | 5.1 |
| Step | Step | 5.0 | 4.9 | 4.8 | 5.3 |
| Skewed | Skewed | 5.0 | 4.9 | 4.9 | 5.2 |
| Uniform | Normal | 5.0 | 5.0 | 5.4 | 5.2 |
| Uniform | U-shaped | 5.1 | 5.1 | 5.5 | 5.1 |
| Normal | U-shaped | 5.2 | 5.1 | 7.5 | 5.2 |
| Mean deviation from 5% | | 0.09 | **0.09** | *0.51* | 0.20 |

| Dist. $X$ ($m$) | Dist. $Y$ ($n$) | $E(X) - E(Y)$ | T | U | WMW | BM |
|---|---|---|---|---|---|---|
| Linear trend | Step | 0.15 | **7.5** | **7.5** | *6.5* | 7.0 |
| Step | Skewed | 0.35 | 21.7 | *21.6* | 26.3 | **27.1** |
| Linear trend | Skewed | 0.50 | 34.5 | *34.4* | 35.1 | **36.4** |
| Uniform | Linear trend | 0.75 | **50.4** | **50.4** | *47.1* | 48.6 |
| Normal | Linear trend | 0.75 | 65.6 | *65.5* | **68.3** | **68.3** |
| U-shaped | Linear trend | 0.75 | **40.2** | 39.9 | 28.5 | *27.3* |
| Uniform | Step | 0.90 | **68.7** | 68.6 | *63.0* | 63.7 |
| Normal | Step | 0.90 | *83.9* | *83.9* | 88.4 | **88.6** |
| U-shaped | Step | 0.90 | **56.2** | 55.7 | 34.6 | *32.6* |
| Uniform | Skewed | 1.25 | **93.1** | 93.0 | *91.7* | 92.2 |
| Normal | Skewed | 1.25 | *98.4* | *98.4* | **99.1** | **99.1** |
| U-shaped | Skewed | 1.25 | **85.2** | 85.0 | 69.7 | *69.3* |
| Sum over all distribution combinations | | | **705.4** | 703.8 | *658.3* | 660.2 |
| Relative power (per cent) | | | **100.0** | 99.8 | *93.3* | 93.6 |

Web Table 22: Estimated true significance levels (top) and power (bottom) for the two-sample T test (T), the Welch U test (U), the Wilcoxon-Mann-Whitney test (WMW), and the Brunner-Munzel test (BM). For notation and definitions, see page 1.

## Outcome scale: {0, 1, 2, 3, 4}

$m = n = 50$

| Dist. $X$ ($m$) | Dist. $Y$ ($n$) | T | U | WMW | BM |
|---|---|---|---|---|---|
| Uniform | Uniform | 5.0 | 5.0 | 4.9 | 5.1 |
| Normal | Normal | 5.0 | 5.0 | 4.9 | 5.2 |
| U-shaped | U-shaped | 5.1 | 5.1 | 5.0 | 5.1 |
| Linear trend | Linear trend | 5.0 | 5.0 | 5.0 | 5.2 |
| Step | Step | 5.0 | 5.0 | 5.0 | 5.1 |
| Skewed | Skewed | 5.0 | 5.0 | 5.0 | 5.2 |
| Uniform | Normal | 5.0 | 5.0 | 5.6 | 5.1 |
| Uniform | U-shaped | 5.1 | 5.0 | 5.6 | 5.1 |
| Normal | U-shaped | 5.2 | 5.1 | 7.8 | 5.1 |
| Mean deviation from 5% | | 0.05 | **0.04** | *0.48* | 0.12 |

| Dist. $X$ ($m$) | Dist. $Y$ ($n$) | $E(X) - E(Y)$ | T | U | WMW | BM |
|---|---|---|---|---|---|---|
| Linear trend | Step | 0.15 | **9.9** | **9.9** | *8.2* | 8.5 |
| Step | Skewed | 0.35 | *37.8* | *37.8* | 47.1 | **47.4** |
| Linear trend | Skewed | 0.50 | 60.6 | *60.5* | 62.2 | **62.8** |
| Uniform | Linear trend | 0.75 | **80.4** | 80.3 | *77.1* | 77.6 |
| Normal | Linear trend | 0.75 | *91.9* | *91.9* | **93.5** | 93.3 |
| U-shaped | Linear trend | 0.75 | **69.3** | 69.2 | 51.0 | *48.8* |
| Uniform | Step | 0.90 | **93.7** | **93.7** | *90.7* | *90.7* |
| Normal | Step | 0.90 | *98.6* | *98.6* | **99.4** | **99.4** |
| U-shaped | Step | 0.90 | **86.2** | 86.1 | 60.4 | *57.7* |
| Uniform | Skewed | 1.25 | **99.8** | **99.8** | *99.7* | *99.7* |
| Normal | Skewed | 1.25 | 100.0 | 100.0 | 100.0 | 100.0 |
| U-shaped | Skewed | 1.25 | **99.0** | **99.0** | 94.1 | *93.8* |
| Sum over all distribution combinations | | | **927.3** | 926.9 | 883.4 | *879.6* |
| Relative power (per cent) | | | **100.0** | 100.0 | 95.3 | *94.8* |

Web Table 23: Estimated true significance levels (top) and power (bottom) for the two-sample T test (T), the Welch U test (U), the Wilcoxon-Mann-Whitney test (WMW), and the Brunner-Munzel test (BM). For notation and definitions, see page 1.

## Outcome scale: {0, 1, 2, 3, 4}

$m = n = 100$

| Dist. $X$ ($m$) | Dist. $Y$ ($n$) | T | U | WMW | BM |
|---|---|---|---|---|---|
| Uniform | Uniform | 4.9 | 4.9 | 4.9 | 5.0 |
| Normal | Normal | 5.0 | 5.0 | 5.0 | 5.1 |
| U-shaped | U-shaped | 5.0 | 5.0 | 5.0 | 5.1 |
| Linear trend | Linear trend | 5.0 | 5.0 | 5.0 | 5.1 |
| Step | Step | 5.0 | 5.0 | 4.9 | 5.0 |
| Skewed | Skewed | 5.0 | 5.0 | 5.0 | 5.1 |
| Uniform | Normal | 5.0 | 4.9 | 5.7 | 5.0 |
| Uniform | U-shaped | 4.9 | 4.9 | 5.6 | 5.0 |
| Normal | U-shaped | 4.9 | 4.9 | 7.7 | 4.9 |
| Mean deviation from 5% | | **0.04** | 0.05 | *0.47* | 0.06 |

| Dist. $X$ ($m$) | Dist. $Y$ ($n$) | $E(X) - E(Y)$ | T | U | WMW | BM |
|---|---|---|---|---|---|---|
| Linear trend | Step | 0.15 | **15.3** | **15.3** | *12.1* | *12.1* |
| Step | Skewed | 0.35 | *64.7* | *64.7* | 76.3 | **76.4** |
| Linear trend | Skewed | 0.50 | *88.5* | *88.5* | 89.9 | **90.0** |
| Uniform | Linear trend | 0.75 | **97.9** | **97.9** | *97.0* | *97.0* |
| Normal | Linear trend | 0.75 | *99.7* | *99.7* | **99.8** | **99.8** |
| U-shaped | Linear trend | 0.75 | **94.0** | **94.0** | 79.7 | *77.8* |
| Uniform | Step | 0.90 | **99.8** | **99.8** | *99.6* | *99.6* |
| Normal | Step | 0.90 | 100.0 | 100.0 | 100.0 | 100.0 |
| U-shaped | Step | 0.90 | **99.1** | **99.1** | 87.7 | *86.0* |
| Uniform | Skewed | 1.25 | 100.0 | 100.0 | 100.0 | 100.0 |
| Normal | Skewed | 1.25 | 100.0 | 100.0 | 100.0 | 100.0 |
| U-shaped | Skewed | 1.25 | **100.0** | **100.0** | *99.9* | *99.9* |
| Sum over all distribution combinations | | | **1059.1** | 1059.1 | 1042.0 | *1038.6* |
| Relative power (per cent) | | | **100.0** | 100.0 | 98.4 | *98.1* |

Web Table 24: Estimated true significance levels (top) and power (bottom) for the two-sample T test (T), the Welch U test (U), the Wilcoxon-Mann-Whitney test (WMW), and the Brunner-Munzel test (BM). For notation and definitions, see page 1.

## Outcome scale: {0, 1, 2, 3, 4}

$m = 25, n = 10$

| Dist. $X$ $(m)$ | Dist. $Y$ $(n)$ | T | U | WMW | BM |
|---|---|---|---|---|---|
| Uniform | Uniform | 5.1 | 5.3 | 4.7 | 5.6 |
| Normal | Normal | 5.2 | 5.2 | 4.7 | 5.7 |
| U-shaped | U-shaped | 5.0 | 5.4 | 4.6 | 5.5 |
| Linear trend | Linear trend | 5.0 | 5.6 | 4.7 | 5.7 |
| Step | Step | 5.0 | 5.6 | 4.7 | 5.7 |
| Skewed | Skewed | 4.7 | 6.3 | 4.7 | 5.9 |
| Uniform | Normal | 2.1 | 4.9 | 2.5 | 5.3 |
| Uniform | U-shaped | 7.5 | 5.5 | 7.6 | 5.5 |
| Normal | U-shaped | 12.7 | 5.6 | 12.7 | 5.4 |
| Normal | Uniform | 9.9 | 5.5 | 8.7 | 5.7 |
| U-shaped | Uniform | 3.3 | 5.3 | 3.3 | 5.6 |
| U-shaped | Normal | 1.5 | 5.0 | 2.6 | 5.3 |
| Mean deviation from 5% | | *1.95* | **0.24** | 1.80 | 0.38 |

| Dist. $X$ $(m)$ | Dist. $Y$ $(n)$ | $E(X) - E(Y)$ | T | U | WMW | BM |
|---|---|---|---|---|---|---|
| Linear trend | Step | 0.15 | 4.9 | **8.3** | *4.7* | 6.9 |
| Step | Skewed | 0.35 | *13.4* | **19.9** | 16.3 | 18.7 |
| Linear trend | Skewed | 0.50 | *19.2* | **27.9** | 20.3 | 25.0 |
| Uniform | Linear trend | 0.75 | 28.7 | **33.6** | *27.2* | 32.7 |
| Normal | Linear trend | 0.75 | **47.9** | *38.5* | 45.4 | 38.6 |
| U-shaped | Linear trend | 0.75 | 18.6 | **29.9** | *14.7* | 23.1 |
| Uniform | Step | 0.90 | 41.1 | **49.4** | *38.3* | 45.9 |
| Normal | Step | 0.90 | 63.9 | *56.4* | **64.5** | 59.7 |
| U-shaped | Step | 0.90 | 27.2 | **43.5** | *18.0* | 27.7 |
| Uniform | Skewed | 1.25 | 71.2 | **76.0** | *70.0* | 74.7 |
| Normal | Skewed | 1.25 | **88.1** | *81.6* | 88.0 | 82.3 |
| U-shaped | Skewed | 1.25 | 54.1 | **69.8** | *43.8* | 57.5 |
| Step | Linear trend | -0.15 | **7.6** | *5.5* | 6.4 | 6.4 |
| Skewed | Step | -0.35 | 15.4 | *9.3* | **16.6** | 16.2 |
| Skewed | Linear trend | -0.50 | **24.6** | *15.4* | 22.8 | 20.5 |
| Linear trend | Uniform | -0.75 | **34.8** | *26.2* | 29.7 | 27.9 |
| Linear trend | Normal | -0.75 | *39.6* | 45.2 | 43.3 | **52.5** |
| Linear trend | U-shaped | -0.75 | **31.7** | 18.5 | 21.2 | *14.1* |
| Step | Uniform | -0.90 | **49.8** | 36.5 | 41.2 | *36.3* |
| Step | Normal | -0.90 | *59.9* | 61.8 | 65.7 | **70.8** |
| Step | U-shaped | -0.90 | **43.5** | 24.9 | 25.0 | *16.2* |
| Skewed | Uniform | -1.25 | **76.4** | *63.2* | 70.6 | 65.4 |
| Skewed | Normal | -1.25 | *87.7* | 88.6 | 91.7 | **93.3** |
| Skewed | U-shaped | -1.25 | **67.6** | 44.9 | 47.0 | *34.9* |
| Sum over all distribution combinations | | | **1016.9** | 975.0 | *932.3* | 947.3 |
| Relative power (per cent) | | | **100.0** | 95.9 | *91.7* | 93.2 |

Web Table 25: Estimated true significance levels (top) and power (bottom) for the two-sample T test (T), the Welch U test (U), the Wilcoxon-Mann-Whitney test (WMW), and the Brunner-Munzel test (BM). For notation and definitions, see page 1.

## Outcome scale: {0, 1, 2, 3, 4}

$m = 50, n = 10$

| Dist. $X$ ($m$) | Dist. $Y$ ($n$) | T | U | WMW | BM |
|---|---|---|---|---|---|
| Uniform | Uniform | 5.0 | 5.5 | 4.8 | 5.7 |
| Normal | Normal | 5.0 | 5.2 | 4.8 | 5.7 |
| U-shaped | U-shaped | 5.0 | 5.6 | 4.8 | 5.7 |
| Linear trend | Linear trend | 5.1 | 6.1 | 4.9 | 5.8 |
| Step | Step | 4.9 | 6.4 | 4.9 | 5.8 |
| Skewed | Skewed | 4.6 | 7.8 | 4.6 | 6.0 |
| Uniform | Normal | 1.2 | 5.1 | 1.4 | 5.5 |
| Uniform | U-shaped | 9.0 | 5.6 | 9.1 | 5.5 |
| Normal | U-shaped | 18.2 | 5.6 | 16.0 | 5.4 |
| Normal | Uniform | 13.0 | 5.4 | 10.8 | 5.6 |
| U-shaped | Uniform | 2.3 | 5.5 | 2.1 | 5.7 |
| U-shaped | Normal | 0.5 | 5.1 | 0.7 | 5.3 |
| Mean deviation from 5% | | *3.03* | **0.30** | 2.70 | 0.42 |

| Dist. $X$ ($m$) | Dist. $Y$ ($n$) | $E(X) - E(Y)$ | T | U | WMW | BM |
|---|---|---|---|---|---|---|
| Linear trend | Step | 0.15 | *4.5* | **10.0** | *4.5* | 7.3 |
| Step | Skewed | 0.35 | *14.2* | **23.4** | 18.6 | 20.4 |
| Linear trend | Skewed | 0.50 | *21.0* | **32.4** | 23.2 | 28.1 |
| Uniform | Linear trend | 0.75 | 32.8 | **38.5** | *32.1* | 37.8 |
| Normal | Linear trend | 0.75 | **56.0** | 40.4 | 51.8 | *40.1* |
| U-shaped | Linear trend | 0.75 | 18.9 | **36.1** | *15.0* | 29.7 |
| Uniform | Step | 0.90 | 46.4 | **56.0** | *44.9* | 53.7 |
| Normal | Step | 0.90 | **71.8** | *59.1* | 71.6 | 62.1 |
| U-shaped | Step | 0.90 | 28.6 | **52.8** | *19.0* | 36.8 |
| Uniform | Skewed | 1.25 | 78.5 | **81.4** | *78.2* | 80.7 |
| Normal | Skewed | 1.25 | **92.9** | *83.7* | 91.9 | *83.7* |
| U-shaped | Skewed | 1.25 | 60.9 | **78.7** | *51.5* | 69.5 |
| Step | Linear trend | -0.15 | **8.3** | *5.5* | 7.1 | 6.2 |
| Skewed | Step | -0.35 | 17.4 | *9.3* | **19.4** | 17.7 |
| Skewed | Linear trend | -0.50 | **29.3** | *15.3* | 27.2 | 21.8 |
| Linear trend | Uniform | -0.75 | **41.3** | *27.6* | 34.8 | 29.3 |
| Linear trend | Normal | -0.75 | *43.9* | 51.6 | 50.9 | **62.4** |
| Linear trend | U-shaped | -0.75 | **39.7** | 18.9 | 25.3 | *14.0* |
| Step | Uniform | -0.90 | **58.2** | 37.9 | 47.4 | *37.8* |
| Step | Normal | -0.90 | *66.9* | 68.2 | 74.8 | **78.0** |
| Step | U-shaped | -0.90 | **53.2** | 25.5 | 29.6 | *16.2* |
| Skewed | Uniform | -1.25 | **84.6** | *65.9* | 78.2 | 68.3 |
| Skewed | Normal | -1.25 | 93.3 | *92.9* | **96.5** | **96.5** |
| Skewed | U-shaped | -1.25 | **77.3** | 45.9 | 53.8 | *35.3* |
| Sum over all distribution combinations | | | **1140.2** | 1057.0 | 1047.3 | *1033.2* |
| Relative power (per cent) | | | **100.0** | 92.7 | 91.9 | *90.6* |

Web Table 26: Estimated true significance levels (top) and power (bottom) for the two-sample T test (T), the Welch U test (U), the Wilcoxon-Mann-Whitney test (WMW), and the Brunner-Munzel test (BM). For notation and definitions, see page 1.

## Outcome scale: {0, 1, 2, 3, 4}

$m = 100, n = 50$

| Dist. $X$ ($m$) | Dist. $Y$ ($n$) | T | U | WMW | BM |
|---|---|---|---|---|---|
| Uniform | Uniform | 5.1 | 5.1 | 5.1 | 5.2 |
| Normal | Normal | 5.0 | 5.0 | 4.9 | 5.1 |
| U-shaped | U-shaped | 5.0 | 5.1 | 5.0 | 5.2 |
| Linear trend | Linear trend | 5.0 | 5.0 | 4.9 | 5.1 |
| Step | Step | 5.0 | 5.1 | 5.0 | 5.2 |
| Skewed | Skewed | 5.0 | 5.2 | 5.0 | 5.2 |
| Uniform | Normal | 2.7 | 5.0 | 3.4 | 5.1 |
| Uniform | U-shaped | 6.7 | 5.0 | 7.4 | 5.0 |
| Normal | U-shaped | 10.2 | 5.0 | 12.0 | 4.9 |
| Normal | Uniform | 8.4 | 5.0 | 8.2 | 5.0 |
| U-shaped | Uniform | 3.6 | 5.0 | 3.9 | 5.1 |
| U-shaped | Normal | 2.0 | 5.1 | 3.8 | 5.1 |
| Mean deviation from 5% | | *1.43* | **0.03** | 1.38 | 0.07 |

| Dist. $X$ ($m$) | Dist. $Y$ ($n$) | $E(X) - E(Y)$ | T | U | WMW | BM |
|---|---|---|---|---|---|---|
| Linear trend | Step | 0.15 | 10.5 | **12.9** | *8.8* | 10.0 |
| Step | Skewed | 0.35 | *47.5* | 49.2 | **58.4** | 58.0 |
| Linear trend | Skewed | 0.50 | *72.0* | 74.1 | 74.6 | **75.8** |
| Uniform | Linear trend | 0.75 | 90.1 | **91.3** | *88.6* | 89.9 |
| Normal | Linear trend | 0.75 | 97.3 | *95.9* | **97.4** | 96.2 |
| U-shaped | Linear trend | 0.75 | 79.9 | **85.7** | *64.4* | 69.8 |
| Uniform | Step | 0.90 | 98.0 | **98.4** | *97.1* | 97.7 |
| Normal | Step | 0.90 | 99.8 | *99.6* | **99.9** | 99.8 |
| U-shaped | Step | 0.90 | 93.7 | **96.4** | *75.0* | 79.5 |
| Uniform | Skewed | 1.25 | 100.0 | 100.0 | 100.0 | 100.0 |
| Normal | Skewed | 1.25 | 100.0 | 100.0 | 100.0 | 100.0 |
| U-shaped | Skewed | 1.25 | **99.9** | **99.9** | *99.0* | 99.3 |
| Step | Linear trend | -0.15 | **12.9** | 10.7 | 10.3 | *9.3* |
| Skewed | Step | -0.35 | 48.5 | *46.1* | 59.6 | **59.7** |
| Skewed | Linear trend | -0.50 | 74.2 | *71.1* | **75.3** | 74.0 |
| Linear trend | Uniform | -0.75 | **90.7** | 88.9 | 87.4 | *86.1* |
| Linear trend | Normal | -0.75 | *97.3* | 98.2 | 98.6 | **99.0** |
| Linear trend | U-shaped | -0.75 | **83.3** | 76.7 | 61.8 | *52.7* |
| Step | Uniform | -0.90 | **98.1** | 97.5 | 96.1 | *95.0* |
| Step | Normal | -0.90 | *99.8* | 99.9 | **100.0** | **100.0** |
| Step | U-shaped | -0.90 | **94.5** | 90.8 | 70.9 | *61.6* |
| Skewed | Uniform | -1.25 | 100.0 | 100.0 | 100.0 | 100.0 |
| Skewed | Normal | -1.25 | 100.0 | 100.0 | 100.0 | 100.0 |
| Skewed | U-shaped | -1.25 | **99.9** | 99.7 | 97.5 | *96.1* |
| Sum over all distribution combinations | | | **1987.9** | 1982.9 | 1920.4 | *1909.3* |
| Relative power (per cent) | | | **100.0** | 99.8 | 96.6 | *96.1* |

Web Table 27: Estimated true significance levels (top) and power (bottom) for the two-sample T test (T), the Welch U test (U), the Wilcoxon-Mann-Whitney test (WMW), and the Brunner-Munzel test (BM). For notation and definitions, see page 1.

## Outcome scale: {0, 1, 2, 3, 4}

$m = 100, n = 25$

| Dist. $X$ ($m$) | Dist. $Y$ ($n$) | T | U | WMW | BM |
|---|---|---|---|---|---|
| Uniform | Uniform | 4.9 | 5.0 | 4.8 | 5.1 |
| Normal | Normal | 4.9 | 5.0 | 4.8 | 5.2 |
| U-shaped | U-shaped | 5.0 | 5.1 | 4.9 | 5.1 |
| Linear trend | Linear trend | 5.0 | 5.2 | 4.9 | 5.2 |
| Step | Step | 5.0 | 5.5 | 4.9 | 5.2 |
| Skewed | Skewed | 4.9 | 6.0 | 4.9 | 5.3 |
| Uniform | Normal | 1.5 | 5.0 | 1.9 | 5.2 |
| Uniform | U-shaped | 8.5 | 5.2 | 9.0 | 5.2 |
| Normal | U-shaped | 16.3 | 5.2 | 15.6 | 5.3 |
| Normal | Uniform | 11.9 | 5.2 | 10.3 | 5.2 |
| U-shaped | Uniform | 2.6 | 5.1 | 2.7 | 5.1 |
| U-shaped | Normal | 0.7 | 5.1 | 1.3 | 5.3 |
| Mean deviation from 5% | | *2.68* | **0.08** | 2.48 | 0.13 |

| Dist. $X$ ($m$) | Dist. $Y$ ($n$) | $E(X) - E(Y)$ | T | U | WMW | BM |
|---|---|---|---|---|---|---|
| Linear trend | Step | 0.15 | 7.1 | **11.3** | *6.4* | 8.5 |
| Step | Skewed | 0.35 | *30.6* | 35.1 | **38.9** | 38.3 |
| Linear trend | Skewed | 0.50 | *48.8* | **54.9** | 52.1 | 54.7 |
| Uniform | Linear trend | 0.75 | 69.3 | **73.2** | *68.1* | 72.0 |
| Normal | Linear trend | 0.75 | **87.1** | 78.2 | 85.3 | *77.7* |
| U-shaped | Linear trend | 0.75 | 52.7 | **68.7** | *41.5* | 56.6 |
| Uniform | Step | 0.90 | 86.7 | **89.8** | *85.2* | 88.4 |
| Normal | Step | 0.90 | 96.5 | *93.0* | **97.1** | 95.1 |
| U-shaped | Step | 0.90 | 73.3 | **86.5** | *52.3* | 67.4 |
| Uniform | Skewed | 1.25 | *99.2* | **99.4** | *99.2* | 99.3 |
| Normal | Skewed | 1.25 | **99.9** | *99.7* | **99.9** | 99.8 |
| U-shaped | Skewed | 1.25 | 97.1 | **98.9** | *92.2* | 96.2 |
| Step | Linear trend | -0.15 | **10.7** | *7.2* | 8.8 | 7.4 |
| Skewed | Step | -0.35 | 32.5 | *25.9* | **40.3** | 38.9 |
| Skewed | Linear trend | -0.50 | **54.4** | *44.3* | 54.2 | 49.5 |
| Linear trend | Uniform | -0.75 | **73.7** | 65.2 | 67.1 | *62.1* |
| Linear trend | Normal | -0.75 | *84.3* | 89.7 | 90.6 | **94.0** |
| Linear trend | U-shaped | -0.75 | **66.6** | 48.5 | 44.0 | *30.2* |
| Step | Uniform | -0.90 | **89.4** | 82.1 | 82.0 | *75.7* |
| Step | Normal | -0.90 | *96.8* | 97.8 | 98.8 | **99.1** |
| Step | U-shaped | -0.90 | **82.4** | 64.6 | 51.4 | *35.8* |
| Skewed | Uniform | -1.25 | **99.3** | 98.5 | 98.7 | *97.8* |
| Skewed | Normal | -1.25 | 100.0 | 100.0 | 100.0 | 100.0 |
| Skewed | U-shaped | -1.25 | **97.6** | 91.9 | 86.0 | *76.4* |
| Sum over all distribution combinations | | | **1736.1** | 1704.5 | 1640.4 | *1620.8* |
| Relative power (per cent) | | | **100.0** | 98.2 | 94.5 | *93.4* |

Web Table 28: Estimated true significance levels (top) and power (bottom) for the two-sample T test (T), the Welch U test (U), the Wilcoxon-Mann-Whitney test (WMW), and the Brunner-Munzel test (BM). For notation and definitions, see page 1.

## Outcome scale: {0, 1, 2, 3, 4}

$m = 100, n = 10$

| Dist. $X$ ($m$) | Dist. $Y$ ($n$) | T | U | WMW | BM |
|---|---|---|---|---|---|
| Uniform | Uniform | 5.0 | 5.5 | 4.9 | 5.7 |
| Normal | Normal | 5.1 | 5.3 | 4.9 | 5.7 |
| U-shaped | U-shaped | 4.9 | 5.6 | 4.8 | 5.7 |
| Linear trend | Linear trend | 4.9 | 6.2 | 4.8 | 5.7 |
| Step | Step | 4.7 | 6.5 | 4.7 | 5.5 |
| Skewed | Skewed | 4.7 | 8.5 | 4.8 | 6.1 |
| Uniform | Normal | 0.7 | 5.4 | 0.8 | 5.6 |
| Uniform | U-shaped | 9.9 | 5.5 | 10.0 | 5.5 |
| Normal | U-shaped | 22.5 | 5.4 | 18.6 | 5.2 |
| Normal | Uniform | 15.5 | 5.5 | 12.4 | 5.6 |
| U-shaped | Uniform | 1.9 | 5.5 | 1.6 | 5.7 |
| U-shaped | Normal | 0.2 | 5.2 | 0.1 | 5.4 |
| Mean deviation from 5% | | *3.77* | **0.33** | 3.23 | 0.42 |

| Dist. $X$ ($m$) | Dist. $Y$ ($n$) | $E(X) - E(Y)$ | T | U | WMW | BM |
|---|---|---|---|---|---|---|
| Linear trend | Step | 0.15 | *4.4* | **10.9** | 4.5 | 7.5 |
| Step | Skewed | 0.35 | *14.9* | **24.9** | 20.2 | 21.1 |
| Linear trend | Skewed | 0.50 | *22.0* | **34.7** | 25.3 | 29.6 |
| Uniform | Linear trend | 0.75 | 34.8 | **40.3** | *34.5* | 39.9 |
| Normal | Linear trend | 0.75 | **60.8** | 41.1 | 55.3 | *40.4* |
| U-shaped | Linear trend | 0.75 | 19.3 | **39.5** | *15.2* | 34.8 |
| Uniform | Step | 0.90 | 50.1 | **58.9** | *49.3* | 57.7 |
| Normal | Step | 0.90 | **76.5** | *60.5* | 75.3 | 63.5 |
| U-shaped | Step | 0.90 | 29.9 | **57.8** | *19.5* | 44.1 |
| Uniform | Skewed | 1.25 | *82.6* | **83.7** | *82.6* | 83.3 |
| Normal | Skewed | 1.25 | **95.1** | 84.9 | 93.7 | *84.7* |
| U-shaped | Skewed | 1.25 | 65.4 | **82.4** | *56.9* | 76.5 |
| Step | Linear trend | -0.15 | **9.2** | *5.6* | 7.9 | 6.4 |
| Skewed | Step | -0.35 | 18.7 | *9.3* | **21.3** | 18.5 |
| Skewed | Linear trend | -0.50 | **32.3** | *15.4* | 30.1 | 22.4 |
| Linear trend | Uniform | -0.75 | **45.7** | *27.8* | 37.9 | 29.9 |
| Linear trend | Normal | -0.75 | *47.0* | 55.0 | 56.0 | **67.7** |
| Linear trend | U-shaped | -0.75 | **44.7** | 18.9 | 27.4 | *14.0* |
| Step | Uniform | -0.90 | **63.0** | 38.6 | 51.2 | *38.4* |
| Step | Normal | -0.90 | *71.6* | *71.6* | 80.1 | **81.3** |
| Step | U-shaped | -0.90 | **59.2** | 25.6 | 32.2 | *16.3* |
| Skewed | Uniform | -1.25 | **88.2** | *67.2* | 81.8 | 69.2 |
| Skewed | Normal | -1.25 | 95.7 | *94.4* | **98.2** | 97.5 |
| Skewed | U-shaped | -1.25 | **81.9** | 45.9 | 57.1 | *35.1* |
| Sum over all distribution combinations | | | **1212.9** | 1094.8 | 1113.4 | *1080.1* |
| Relative power (per cent) | | | **100.0** | 90.3 | 91.8 | *89.1* |

Web Table 29: Estimated true significance levels (top) and power (bottom) for the two-sample T test (T), the Welch U test (U), the Wilcoxon-Mann-Whitney test (WMW), and the Brunner-Munzel test (BM). For notation and definitions, see page 1.

## Outcome scale: {0, 1, 2, 3, 4, 5}

$m = n = 10$

| Dist. $X$ ($m$) | Dist. $Y$ ($n$) | T | U | WMW | BM |
|---|---|---|---|---|---|
| Uniform | Uniform | 5.2 | 5.0 | 4.4 | 5.6 |
| Normal | Normal | 5.1 | 4.8 | 4.5 | 5.9 |
| U-shaped | U-shaped | 5.3 | 5.2 | 4.4 | 5.4 |
| Linear trend | Linear trend | 5.1 | 4.8 | 4.4 | 5.6 |
| Step | Step | 4.8 | 4.5 | 4.4 | 5.6 |
| Skewed | Skewed | 4.7 | 4.0 | 4.5 | 5.7 |
| Uniform | Normal | 5.5 | 5.2 | 5.5 | 5.6 |
| Uniform | U-shaped | 5.4 | 5.2 | 5.2 | 5.5 |
| Normal | U-shaped | 6.0 | 5.5 | 8.2 | 5.2 |
| Mean deviation from 5% | | 0.33 | **0.33** | *0.81* | 0.57 |

| Dist. $X$ ($m$) | Dist. $Y$ ($n$) | $E(X) - E(Y)$ | T | U | WMW | BM |
|---|---|---|---|---|---|---|
| Linear trend | Step | 0.025 | 5.2 | 4.9 | *4.6* | **5.8** |
| Step | Skewed | 0.720 | 21.7 | *20.5* | 21.4 | **24.5** |
| Linear trend | Skewed | 0.745 | 23.1 | 22.0 | *20.8* | **23.8** |
| Uniform | Linear trend | 0.875 | 21.5 | 21.1 | *18.7* | **21.8** |
| Normal | Linear trend | 0.875 | 33.1 | *32.2* | 32.3 | **33.5** |
| U-shaped | Linear trend | 0.875 | **16.5** | 15.7 | *12.0* | 12.3 |
| Uniform | Step | 0.900 | **23.4** | 22.8 | *20.0* | 22.5 |
| Normal | Step | 0.900 | 36.2 | *35.3* | 39.4 | **40.7** |
| U-shaped | Step | 0.900 | **17.7** | 16.8 | *11.3* | 11.4 |
| Uniform | Skewed | 1.620 | **64.0** | 62.8 | *58.5* | 62.2 |
| Normal | Skewed | 1.620 | 84.9 | *84.3* | **86.4** | 85.0 |
| U-shaped | Skewed | 1.620 | **49.1** | 46.5 | *33.0* | 34.2 |
| Sum over all distribution combinations | | | **396.2** | 384.9 | *358.3* | 377.6 |
| Relative power (per cent) | | | **100.0** | 97.1 | *90.4* | 95.3 |

Web Table 30: Estimated true significance levels (top) and power (bottom) for the two-sample T test (T), the Welch U test (U), the Wilcoxon-Mann-Whitney test (WMW), and the Brunner-Munzel test (BM). For notation and definitions, see page 1.

## Outcome scale: {0, 1, 2, 3, 4, 5}

$m = n = 25$

| Dist. $X$ ($m$) | Dist. $Y$ ($n$) | T | U | WMW | BM |
|---|---|---|---|---|---|
| Uniform | Uniform | 5.0 | 4.9 | 4.7 | 5.1 |
| Normal | Normal | 5.0 | 5.0 | 4.8 | 5.3 |
| U-shaped | U-shaped | 5.2 | 5.2 | 5.0 | 5.3 |
| Linear trend | Linear trend | 5.0 | 5.0 | 4.7 | 5.1 |
| Step | Step | 4.9 | 4.9 | 4.8 | 5.2 |
| Skewed | Skewed | 5.0 | 4.9 | 4.9 | 5.3 |
| Uniform | Normal | 5.1 | 5.0 | 5.8 | 5.1 |
| Uniform | U-shaped | 5.2 | 5.2 | 5.7 | 5.3 |
| Normal | U-shaped | 5.3 | 5.1 | 8.4 | 5.2 |
| Mean deviation from 5% | | 0.11 | **0.10** | *0.66* | 0.21 |

| Dist. $X$ ($m$) | Dist. $Y$ ($n$) | $E(X) - E(Y)$ | T | U | WMW | BM |
|---|---|---|---|---|---|---|
| Linear trend | Step | 0.025 | 5.1 | 5.1 | *4.9* | **5.2** |
| Step | Skewed | 0.720 | 48.2 | *48.1* | 50.1 | **51.4** |
| Linear trend | Skewed | 0.745 | **50.3** | 50.1 | *48.6* | 50.0 |
| Uniform | Linear trend | 0.875 | **47.8** | 47.7 | *44.4* | 45.8 |
| Normal | Linear trend | 0.875 | 66.6 | *66.3* | **69.2** | 67.9 |
| U-shaped | Linear trend | 0.875 | **37.1** | 36.8 | 25.2 | *23.7* |
| Uniform | Step | 0.900 | **50.2** | 50.1 | *46.5* | 47.2 |
| Normal | Step | 0.900 | 69.2 | *68.9* | **79.4** | 78.2 |
| U-shaped | Step | 0.900 | **39.1** | 38.8 | 22.4 | *20.7* |
| Uniform | Skewed | 1.620 | **96.5** | **96.5** | *95.1* | 95.3 |
| Normal | Skewed | 1.620 | *99.7* | *99.7* | **99.9** | **99.9** |
| U-shaped | Skewed | 1.620 | **89.8** | 89.5 | 71.3 | *70.5* |
| Sum over all distribution combinations | | | **699.6** | 697.6 | 656.9 | *656.0* |
| Relative power (per cent) | | | **100.0** | 99.7 | 93.9 | *93.8* |

Web Table 31: Estimated true significance levels (top) and power (bottom) for the two-sample T test (T), the Welch U test (U), the Wilcoxon-Mann-Whitney test (WMW), and the Brunner-Munzel test (BM). For notation and definitions, see page 1.

## Outcome scale: {0, 1, 2, 3, 4, 5}

$m = n = 50$

| Dist. $X$ ($m$) | Dist. $Y$ ($n$) | T | U | WMW | BM |
|---|---|---|---|---|---|
| Uniform | Uniform | 5.0 | 5.0 | 5.0 | 5.2 |
| Normal | Normal | 5.0 | 5.0 | 5.0 | 5.2 |
| U-shaped | U-shaped | 5.1 | 5.1 | 5.0 | 5.2 |
| Linear trend | Linear trend | 5.0 | 5.0 | 5.0 | 5.2 |
| Step | Step | 5.2 | 5.1 | 5.0 | 5.2 |
| Skewed | Skewed | 5.0 | 4.9 | 5.0 | 5.1 |
| Uniform | Normal | 5.0 | 4.9 | 6.0 | 4.9 |
| Uniform | U-shaped | 5.0 | 5.0 | 5.8 | 5.1 |
| Normal | U-shaped | 5.2 | 5.1 | 8.5 | 5.0 |
| Mean deviation from 5% | | 0.07 | **0.06** | *0.61* | 0.15 |

| Dist. $X$ ($m$) | Dist. $Y$ ($n$) | $E(X) - E(Y)$ | T | U | WMW | BM |
|---|---|---|---|---|---|---|
| Linear trend | Step | 0.025 | **5.1** | **5.1** | *5.0* | **5.1** |
| Step | Skewed | 0.720 | *78.2* | *78.2* | 80.5 | **80.9** |
| Linear trend | Skewed | 0.745 | **80.4** | **80.4** | *79.1* | 79.6 |
| Uniform | Linear trend | 0.875 | **77.2** | **77.2** | *73.9* | 74.5 |
| Normal | Linear trend | 0.875 | *92.4* | *92.4* | **93.8** | 93.1 |
| U-shaped | Linear trend | 0.875 | **64.6** | 64.5 | 44.7 | *42.1* |
| Uniform | Step | 0.900 | **79.4** | **79.4** | *76.1* | *76.1* |
| Normal | Step | 0.900 | 93.5 | *93.4* | **97.6** | 97.3 |
| U-shaped | Step | 0.900 | **67.4** | 67.2 | 39.4 | *36.6* |
| Uniform | Skewed | 1.620 | **100.0** | **100.0** | *99.9* | *99.9* |
| Normal | Skewed | 1.620 | 100.0 | 100.0 | 100.0 | 100.0 |
| U-shaped | Skewed | 1.620 | **99.6** | **99.6** | 94.7 | *94.2* |
| Sum over all distribution combinations | | | **937.7** | 937.3 | 884.7 | *879.4* |
| Relative power (per cent) | | | **100.0** | 100.0 | 94.3 | *93.8* |

Web Table 32: Estimated true significance levels (top) and power (bottom) for the two-sample T test (T), the Welch U test (U), the Wilcoxon-Mann-Whitney test (WMW), and the Brunner-Munzel test (BM). For notation and definitions, see page 1.

## Outcome scale: $\{0, 1, 2, 3, 4, 5\}$

$m = n = 100$

| Dist. $X$ $(m)$ | Dist. $Y$ $(n)$ | T | U | WMW | BM |
|---|---|---|---|---|---|
| Uniform | Uniform | 5.0 | 5.0 | 5.0 | 5.1 |
| Normal | Normal | 5.1 | 5.1 | 5.1 | 5.2 |
| U-shaped | U-shaped | 5.0 | 5.0 | 4.9 | 5.0 |
| Linear trend | Linear trend | 5.0 | 5.0 | 5.0 | 5.1 |
| Step | Step | 4.9 | 4.9 | 4.9 | 5.0 |
| Skewed | Skewed | 5.1 | 5.1 | 5.1 | 5.2 |
| Uniform | Normal | 5.0 | 5.0 | 6.2 | 5.0 |
| Uniform | U-shaped | 5.1 | 5.0 | 5.8 | 5.0 |
| Normal | U-shaped | 5.1 | 5.0 | 8.6 | 5.0 |
| Mean deviation from 5% | | 0.06 | **0.05** | *0.67* | 0.07 |

| Dist. $X$ $(m)$ | Dist. $Y$ $(n)$ | $E(X) - E(Y)$ | T | U | WMW | BM |
|---|---|---|---|---|---|---|
| Linear trend | Step | 0.025 | *5.1* | *5.1* | *5.1* | **5.2** |
| Step | Skewed | 0.720 | *97.2* | *97.2* | 97.8 | **97.9** |
| Linear trend | Skewed | 0.745 | **97.7** | **97.7** | *97.3* | 97.4 |
| Uniform | Linear trend | 0.875 | **97.0** | **97.0** | *95.9* | 96.0 |
| Normal | Linear trend | 0.875 | *99.8* | *99.8* | **99.9** | *99.8* |
| U-shaped | Linear trend | 0.875 | **91.5** | 91.4 | 72.6 | *70.1* |
| Uniform | Step | 0.900 | **97.6** | **97.6** | 96.6 | *96.5* |
| Normal | Step | 0.900 | *99.8* | *99.8* | **100.0** | **100.0** |
| U-shaped | Step | 0.900 | **93.0** | 92.9 | 66.2 | *63.2* |
| Uniform | Skewed | 1.620 | 100.0 | 100.0 | 100.0 | 100.0 |
| Normal | Skewed | 1.620 | 100.0 | 100.0 | 100.0 | 100.0 |
| U-shaped | Skewed | 1.620 | **100.0** | **100.0** | *99.9* | *99.9* |
| Sum over all distribution combinations | | | **1078.7** | 1078.7 | 1031.2 | *1025.8* |
| Relative power (per cent) | | | **100.0** | 100.0 | 95.6 | *95.1* |

Web Table 33: Estimated true significance levels (top) and power (bottom) for the two-sample T test (T), the Welch U test (U), the Wilcoxon-Mann-Whitney test (WMW), and the Brunner-Munzel test (BM). For notation and definitions, see page 1.

## Outcome scale: {0, 1, 2, 3, 4, 5}

$m = 25, n = 10$

| Dist. $X$ ($m$) | Dist. $Y$ ($n$) | T | U | WMW | BM |
|---|---|---|---|---|---|
| Uniform | Uniform | 5.1 | 5.4 | 4.7 | 5.7 |
| Normal | Normal | 5.1 | 5.1 | 4.7 | 5.7 |
| U-shaped | U-shaped | 5.0 | 5.5 | 4.6 | 5.6 |
| Linear trend | Linear trend | 5.0 | 5.4 | 4.6 | 5.6 |
| Step | Step | 4.9 | 5.7 | 4.6 | 5.7 |
| Skewed | Skewed | 4.6 | 6.1 | 4.5 | 5.7 |
| Uniform | Normal | 1.7 | 5.0 | 2.3 | 5.3 |
| Uniform | U-shaped | 7.9 | 5.5 | 8.1 | 5.6 |
| Normal | U-shaped | 15.0 | 5.5 | 13.8 | 5.2 |
| Normal | Uniform | 12.0 | 5.6 | 10.0 | 5.7 |
| U-shaped | Uniform | 3.0 | 5.0 | 2.9 | 5.3 |
| U-shaped | Normal | 1.1 | 5.0 | 2.6 | 5.2 |
| Mean deviation from 5% | | *2.44* | **0.22** | 2.08 | 0.34 |

| Dist. $X$ ($m$) | Dist. $Y$ ($n$) | $E(X) - E(Y)$ | T | U | WMW | BM |
|---|---|---|---|---|---|---|
| Linear trend | Step | 0.025 | 4.7 | **5.8** | *4.1* | 5.6 |
| Step | Skewed | 0.720 | *26.2* | **37.1** | 29.9 | 33.8 |
| Linear trend | Skewed | 0.745 | *27.5* | **38.5** | 28.1 | 34.2 |
| Uniform | Linear trend | 0.875 | 26.9 | **31.5** | *25.6* | 30.5 |
| Normal | Linear trend | 0.875 | **51.1** | 36.9 | 46.8 | *36.4* |
| U-shaped | Linear trend | 0.875 | 16.4 | **27.4** | *12.2* | 20.0 |
| Uniform | Step | 0.900 | 28.8 | **34.5** | *26.9* | 32.1 |
| Normal | Step | 0.900 | 53.9 | *40.4* | **55.3** | 45.1 |
| U-shaped | Step | 0.900 | 17.6 | **29.9** | *11.1* | 17.8 |
| Uniform | Skewed | 1.620 | 79.0 | **83.5** | *77.4* | 81.8 |
| Normal | Skewed | 1.620 | **95.2** | *89.3* | 94.9 | 89.5 |
| U-shaped | Skewed | 1.620 | 60.5 | **77.1** | *45.5* | 59.6 |
| Step | Linear trend | -0.025 | 5.4 | 5.6 | *5.3* | **5.8** |
| Skewed | Step | -0.720 | **34.1** | *20.8* | 31.9 | 30.3 |
| Skewed | Linear trend | -0.745 | **35.6** | *22.3* | 30.8 | 27.8 |
| Linear trend | Uniform | -0.875 | **32.5** | *24.8* | 27.7 | 26.5 |
| Linear trend | Normal | -0.875 | *37.7* | 49.6 | 43.9 | **56.4** |
| Linear trend | U-shaped | -0.875 | **29.9** | 17.0 | 19.5 | *12.7* |
| Step | Uniform | -0.900 | **34.5** | *26.0* | 29.3 | 27.1 |
| Step | Normal | -0.900 | *41.0* | 52.4 | 54.7 | **65.3** |
| Step | U-shaped | -0.900 | **31.4** | 17.8 | 18.2 | *11.8* |
| Skewed | Uniform | -1.620 | **83.8** | *70.6* | 76.6 | 71.1 |
| Skewed | Normal | -1.620 | *95.3* | 96.8 | 97.8 | **98.6** |
| Skewed | U-shaped | -1.620 | **73.4** | 48.9 | 47.6 | *35.1* |
| Sum over all distribution combinations | | | **1022.4** | 984.6 | *941.2* | 954.9 |
| Relative power (per cent) | | | **100.0** | 96.3 | *92.1* | 93.4 |

Web Table 34: Estimated true significance levels (top) and power (bottom) for the two-sample T test (T), the Welch U test (U), the Wilcoxon-Mann-Whitney test (WMW), and the Brunner-Munzel test (BM). For notation and definitions, see page 1.

## Outcome scale: {0, 1, 2, 3, 4, 5}

$m = 50, n = 10$

| Dist. $X$ ($m$) | Dist. $Y$ ($n$) | T | U | WMW | BM |
|---|---|---|---|---|---|
| Uniform | Uniform | 5.0 | 5.5 | 4.9 | 5.7 |
| Normal | Normal | 4.9 | 5.1 | 4.6 | 5.6 |
| U-shaped | U-shaped | 5.0 | 5.7 | 4.8 | 5.7 |
| Linear trend | Linear trend | 4.9 | 5.9 | 4.8 | 5.7 |
| Step | Step | 4.8 | 6.3 | 4.7 | 5.6 |
| Skewed | Skewed | 4.6 | 7.8 | 4.7 | 5.9 |
| Uniform | Normal | 0.6 | 5.1 | 0.9 | 5.4 |
| Uniform | U-shaped | 9.5 | 5.5 | 9.7 | 5.5 |
| Normal | U-shaped | 22.5 | 5.6 | 18.4 | 5.1 |
| Normal | Uniform | 16.8 | 5.4 | 12.6 | 5.5 |
| U-shaped | Uniform | 2.1 | 5.5 | 2.0 | 5.7 |
| U-shaped | Normal | 0.2 | 5.0 | 0.5 | 5.2 |
| Mean deviation from 5% | | *3.84* | **0.29** | 3.17 | 0.38 |

| Dist. $X$ ($m$) | Dist. $Y$ ($n$) | $E(X) - E(Y)$ | T | U | WMW | BM |
|---|---|---|---|---|---|---|
| Linear trend | Step | 0.025 | 4.6 | **6.8** | *4.1* | 5.9 |
| Step | Skewed | 0.720 | *29.0* | **43.3** | 34.6 | 37.5 |
| Linear trend | Skewed | 0.745 | *30.6* | **44.7** | 32.6 | 38.6 |
| Uniform | Linear trend | 0.875 | 30.9 | **36.0** | *30.2* | 35.3 |
| Normal | Linear trend | 0.875 | **60.4** | 38.1 | 52.9 | *37.1* |
| U-shaped | Linear trend | 0.875 | 16.5 | **33.6** | *12.2* | 26.3 |
| Uniform | Step | 0.900 | 32.5 | **39.2** | *31.5* | 37.5 |
| Normal | Step | 0.900 | **62.9** | *42.0* | 61.7 | 46.5 |
| U-shaped | Step | 0.900 | 17.5 | **36.8** | *10.5* | 23.3 |
| Uniform | Skewed | 1.620 | 85.6 | **88.1** | *85.2* | 87.4 |
| Normal | Skewed | 1.620 | **97.8** | *90.7* | 96.8 | *90.7* |
| U-shaped | Skewed | 1.620 | 67.7 | **85.6** | *53.6* | 73.0 |
| Step | Linear trend | -0.025 | *5.2* | **5.8** | 5.6 | 5.6 |
| Skewed | Step | -0.720 | **40.4** | *20.9* | 37.5 | 32.7 |
| Skewed | Linear trend | -0.745 | **42.4** | *22.4* | 36.4 | 29.4 |
| Linear trend | Uniform | -0.875 | **38.7** | *25.9* | 32.4 | 28.1 |
| Linear trend | Normal | -0.875 | *40.4* | 59.2 | 51.5 | **70.2** |
| Linear trend | U-shaped | -0.875 | **37.5** | 17.5 | 23.5 | *12.6* |
| Step | Uniform | -0.900 | **41.2** | *27.4* | 34.8 | 29.2 |
| Step | Normal | -0.900 | *44.1* | 61.8 | 64.5 | **77.4** |
| Step | U-shaped | -0.900 | **39.4** | 18.3 | 22.1 | *11.8* |
| Skewed | Uniform | -1.620 | **90.0** | *73.0* | 82.9 | 73.5 |
| Skewed | Normal | -1.620 | *98.3* | 98.8 | 99.5 | **99.6** |
| Skewed | U-shaped | -1.620 | **82.3** | 49.6 | 54.0 | *35.3* |
| Sum over all distribution combinations | | | **1135.7** | 1065.6 | 1050.6 | *1044.7* |
| Relative power (per cent) | | | **100.0** | 93.8 | 92.5 | *92.0* |

Web Table 35: Estimated true significance levels (top) and power (bottom) for the two-sample T test (T), the Welch U test (U), the Wilcoxon-Mann-Whitney test (WMW), and the Brunner-Munzel test (BM). For notation and definitions, see page 1.

## Outcome scale: {0, 1, 2, 3, 4, 5}

$m = 100, n = 50$

| Dist. $X$ ($m$) | Dist. $Y$ ($n$) | T | U | WMW | BM |
|---|---|---|---|---|---|
| Uniform | Uniform | 4.9 | 5.0 | 4.9 | 5.0 |
| Normal | Normal | 5.1 | 5.1 | 5.0 | 5.2 |
| U-shaped | U-shaped | 5.1 | 5.1 | 5.0 | 5.1 |
| Linear trend | Linear trend | 5.1 | 5.1 | 4.9 | 5.1 |
| Step | Step | 4.9 | 5.1 | 5.0 | 5.1 |
| Skewed | Skewed | 4.8 | 5.1 | 4.8 | 5.0 |
| Uniform | Normal | 2.1 | 5.1 | 3.2 | 5.1 |
| Uniform | U-shaped | 7.0 | 5.1 | 7.9 | 5.0 |
| Normal | U-shaped | 11.8 | 4.9 | 13.5 | 5.0 |
| Normal | Uniform | 9.9 | 5.1 | 9.5 | 5.1 |
| U-shaped | Uniform | 3.5 | 5.0 | 3.8 | 5.1 |
| U-shaped | Normal | 1.5 | 5.0 | 3.9 | 5.0 |
| Mean deviation from 5% | | *1.81* | **0.04** | 1.68 | 0.05 |

| Dist. $X$ ($m$) | Dist. $Y$ ($n$) | $E(X) - E(Y)$ | T | U | WMW | BM |
|---|---|---|---|---|---|---|
| Linear trend | Step | 0.025 | 5.0 | **5.3** | *4.6* | 5.1 |
| Step | Skewed | 0.720 | *87.8* | 89.0 | 90.1 | **90.5** |
| Linear trend | Skewed | 0.745 | 89.2 | **90.5** | *89.0* | 90.1 |
| Uniform | Linear trend | 0.875 | 87.7 | **89.0** | *86.1* | 87.4 |
| Normal | Linear trend | 0.875 | **97.6** | 95.4 | 97.3 | *95.2* |
| U-shaped | Linear trend | 0.875 | 75.5 | **82.5** | *56.8* | 62.8 |
| Uniform | Step | 0.900 | 89.4 | **90.5** | *87.9* | 89.0 |
| Normal | Step | 0.900 | 98.0 | *96.2* | **99.2** | 98.4 |
| U-shaped | Step | 0.900 | 77.8 | **84.3** | *49.8* | 55.5 |
| Uniform | Skewed | 1.620 | 100.0 | 100.0 | 100.0 | 100.0 |
| Normal | Skewed | 1.620 | 100.0 | 100.0 | 100.0 | 100.0 |
| U-shaped | Skewed | 1.620 | **100.0** | **100.0** | *99.2* | 99.5 |
| Step | Linear trend | -0.025 | 5.2 | *5.0* | **5.4** | 5.1 |
| Skewed | Step | -0.720 | 89.3 | *87.5* | **90.4** | 89.9 |
| Skewed | Linear trend | -0.745 | **90.5** | 88.5 | 88.7 | *87.6* |
| Linear trend | Uniform | -0.875 | **88.4** | 86.3 | 84.7 | *83.4* |
| Linear trend | Normal | -0.875 | *97.5* | 98.7 | 98.9 | **99.3** |
| Linear trend | U-shaped | -0.875 | **79.7** | 72.1 | 55.1 | *45.3* |
| Step | Uniform | -0.900 | **90.0** | 88.1 | 86.5 | *84.7* |
| Step | Normal | -0.900 | *98.2* | 99.1 | 99.8 | **99.9** |
| Step | U-shaped | -0.900 | **81.8** | 74.5 | 49.7 | *39.9* |
| Skewed | Uniform | -1.620 | 100.0 | 100.0 | 100.0 | 100.0 |
| Skewed | Normal | -1.620 | 100.0 | 100.0 | 100.0 | 100.0 |
| Skewed | U-shaped | -1.620 | **100.0** | 99.9 | 97.7 | *96.2* |
| Sum over all distribution combinations | | | **2028.5** | 2022.5 | 1916.9 | *1904.6* |
| Relative power (per cent) | | | **100.0** | 99.7 | 94.5 | *93.9* |

Web Table 36: Estimated true significance levels (top) and power (bottom) for the two-sample T test (T), the Welch U test (U), the Wilcoxon-Mann-Whitney test (WMW), and the Brunner-Munzel test (BM). For notation and definitions, see page 1.

## Outcome scale: {0, 1, 2, 3, 4, 5}

$m = 100, n = 25$

| Dist. $X$ ($m$) | Dist. $Y$ ($n$) | T | U | WMW | BM |
|---|---|---|---|---|---|
| Uniform | Uniform | 5.1 | 5.2 | 5.0 | 5.3 |
| Normal | Normal | 5.0 | 4.9 | 4.9 | 5.1 |
| U-shaped | U-shaped | 5.0 | 5.2 | 4.9 | 5.2 |
| Linear trend | Linear trend | 4.9 | 5.2 | 4.8 | 5.1 |
| Step | Step | 4.9 | 5.4 | 4.8 | 5.1 |
| Skewed | Skewed | 4.9 | 6.0 | 4.8 | 5.2 |
| Uniform | Normal | 0.8 | 4.9 | 1.3 | 5.0 |
| Uniform | U-shaped | 8.9 | 5.2 | 9.5 | 5.2 |
| Normal | U-shaped | 19.6 | 5.1 | 17.3 | 5.1 |
| Normal | Uniform | 15.1 | 5.1 | 12.1 | 5.2 |
| U-shaped | Uniform | 2.4 | 5.1 | 2.4 | 5.2 |
| U-shaped | Normal | 0.3 | 4.9 | 0.9 | 5.0 |
| Mean deviation from 5% | | *3.35* | **0.09** | 2.88 | 0.12 |

| Dist. $X$ ($m$) | Dist. $Y$ ($n$) | $E(X) - E(Y)$ | T | U | WMW | BM |
|---|---|---|---|---|---|---|
| Linear trend | Step | 0.025 | 4.9 | **5.8** | *4.4* | 5.3 |
| Step | Skewed | 0.720 | *65.7* | **71.4** | 70.5 | 71.2 |
| Linear trend | Skewed | 0.745 | *67.9* | **73.5** | 69.0 | 72.0 |
| Uniform | Linear trend | 0.875 | 66.0 | **69.8** | *64.9* | 68.4 |
| Normal | Linear trend | 0.875 | **88.7** | 75.9 | 85.1 | *74.2* |
| U-shaped | Linear trend | 0.875 | 47.2 | **64.7** | *34.7* | 50.5 |
| Uniform | Step | 0.900 | 68.8 | **72.3** | *67.4* | 70.7 |
| Normal | Step | 0.900 | 89.9 | *78.2* | **91.8** | 84.2 |
| U-shaped | Step | 0.900 | 50.4 | **67.4** | *29.6* | 44.1 |
| Uniform | Skewed | 1.620 | 99.8 | 99.8 | 99.8 | 99.8 |
| Normal | Skewed | 1.620 | 100.0 | 100.0 | 100.0 | 100.0 |
| U-shaped | Skewed | 1.620 | 98.7 | **99.7** | *93.6* | 97.2 |
| Step | Linear trend | -0.025 | 5.3 | *5.2* | **5.7** | *5.2* |
| Skewed | Step | -0.720 | 71.7 | *61.5* | **71.8** | 68.7 |
| Skewed | Linear trend | -0.745 | **73.8** | *63.2* | 69.5 | 64.3 |
| Linear trend | Uniform | -0.875 | **70.2** | 61.9 | 63.8 | *59.2* |
| Linear trend | Normal | -0.875 | *84.6* | 93.4 | 92.6 | **96.7** |
| Linear trend | U-shaped | -0.875 | **62.7** | 44.1 | 39.2 | *25.7* |
| Step | Uniform | -0.900 | **72.9** | 64.4 | 66.2 | *60.4* |
| Step | Normal | -0.900 | *86.6* | 94.4 | 97.0 | **98.6** |
| Step | U-shaped | -0.900 | **65.3** | 46.3 | 35.8 | *22.9* |
| Skewed | Uniform | -1.620 | **99.8** | 99.5 | 99.4 | *98.8* |
| Skewed | Normal | -1.620 | 100.0 | 100.0 | 100.0 | 100.0 |
| Skewed | U-shaped | -1.620 | **98.7** | 94.6 | 86.3 | *76.6* |
| Sum over all distribution combinations | | | **1739.5** | 1706.7 | 1637.9 | *1614.9* |
| Relative power (per cent) | | | **100.0** | 98.1 | 94.2 | *92.8* |

Web Table 37: Estimated true significance levels (top) and power (bottom) for the two-sample T test (T), the Welch U test (U), the Wilcoxon-Mann-Whitney test (WMW), and the Brunner-Munzel test (BM). For notation and definitions, see page 1.

## Outcome scale: {0, 1, 2, 3, 4, 5}

$m = 100, n = 10$

| Dist. $X$ $(m)$ | Dist. $Y$ $(n)$ | T | U | WMW | BM |
|---|---|---|---|---|---|
| Uniform | Uniform | 4.9 | 5.5 | 4.9 | 5.6 |
| Normal | Normal | 4.9 | 5.1 | 4.7 | 5.6 |
| U-shaped | U-shaped | 4.9 | 5.6 | 4.8 | 5.6 |
| Linear trend | Linear trend | 5.0 | 6.3 | 4.9 | 5.8 |
| Step | Step | 4.7 | 6.9 | 4.8 | 5.6 |
| Skewed | Skewed | 4.6 | 8.6 | 4.8 | 6.0 |
| Uniform | Normal | 0.3 | 5.2 | 0.4 | 5.4 |
| Uniform | U-shaped | 10.7 | 5.7 | 10.9 | 5.6 |
| Normal | U-shaped | 28.9 | 5.6 | 21.9 | 5.2 |
| Normal | Uniform | 20.8 | 5.6 | 14.6 | 5.6 |
| U-shaped | Uniform | 1.7 | 5.6 | 1.4 | 5.8 |
| U-shaped | Normal | 0.0 | 5.2 | 0.0 | 5.2 |
| Mean deviation from 5% | | *4.89* | **0.33** | 3.84 | 0.39 |

| Dist. $X$ $(m)$ | Dist. $Y$ $(n)$ | $E(X) - E(Y)$ | T | U | WMW | BM |
|---|---|---|---|---|---|---|
| Linear trend | Step | 0.025 | 4.6 | **7.1** | *4.1* | 5.8 |
| Step | Skewed | 0.720 | *31.0* | **46.0** | 37.9 | 39.2 |
| Linear trend | Skewed | 0.745 | *32.4* | **47.2** | 35.4 | 40.9 |
| Uniform | Linear trend | 0.875 | 32.8 | **37.8** | *32.4* | 37.3 |
| Normal | Linear trend | 0.875 | **65.8** | 39.0 | 56.6 | *37.8* |
| U-shaped | Linear trend | 0.875 | 16.5 | **36.6** | *11.9* | 31.1 |
| Uniform | Step | 0.900 | 35.1 | **41.3** | *34.6* | 40.5 |
| Normal | Step | 0.900 | **68.4** | *42.8* | 65.4 | 47.2 |
| U-shaped | Step | 0.900 | 17.6 | **40.5** | *10.0* | 28.2 |
| Uniform | Skewed | 1.620 | 89.3 | **90.1** | *89.2* | 89.8 |
| Normal | Skewed | 1.620 | **98.6** | 91.3 | 97.5 | *90.9* |
| U-shaped | Skewed | 1.620 | 73.0 | **89.0** | *59.4* | 81.5 |
| Step | Linear trend | -0.025 | *5.3* | **5.9** | 5.8 | 5.6 |
| Skewed | Step | -0.720 | **44.4** | *21.0* | 41.1 | 33.9 |
| Skewed | Linear trend | -0.745 | **46.7** | *22.9* | 40.3 | 30.7 |
| Linear trend | Uniform | -0.875 | **42.8** | *26.7* | 35.6 | 29.0 |
| Linear trend | Normal | -0.875 | *42.8* | 64.7 | 57.6 | **78.0** |
| Linear trend | U-shaped | -0.875 | **42.3** | 17.3 | 25.3 | *12.4* |
| Step | Uniform | -0.900 | **45.1** | *27.7* | 37.8 | 29.8 |
| Step | Normal | -0.900 | *46.9* | 67.3 | 71.1 | **83.0** |
| Step | U-shaped | -0.900 | **44.5** | 18.3 | 24.2 | *11.9* |
| Skewed | Uniform | -1.620 | **93.0** | *74.0* | 86.0 | 74.5 |
| Skewed | Normal | -1.620 | *99.3* | *99.3* | **99.9** | 99.8 |
| Skewed | U-shaped | -1.620 | **86.8** | 50.1 | 57.7 | *34.8* |
| Sum over all distribution combinations | | | **1204.8** | 1103.8 | 1117.0 | *1093.5* |
| Relative power (per cent) | | | **100.0** | 91.6 | 92.7 | *90.8* |

Web Table 38: Estimated true significance levels (top) and power (bottom) for the two-sample T test (T), the Welch U test (U), the Wilcoxon-Mann-Whitney test (WMW), and the Brunner-Munzel test (BM). For notation and definitions, see page 1.

## Outcome scale: {0, 1, 2}

$m = n = 10$

| Dist. $X$ ($m$) | Dist. $Y$ ($n$) | Coverage prob. U | PB | Bt | Interval length U | PB | Bt |
|---|---|---|---|---|---|---|---|
| Uniform | Uniform | 95.2 | 94.5 | 95.7 | 1.54 | 1.34 | 1.56 |
| Normal | Normal | 95.6 | 94.1 | 95.8 | 1.18 | 1.03 | 1.20 |
| U-shaped | U-shaped | 94.6 | 94.3 | 95.8 | 1.68 | 1.48 | 1.72 |
| Step1 | Step1 | 95.3 | 94.3 | 95.6 | 1.40 | 1.23 | 1.43 |
| Linear trend | Linear trend | 95.5 | 94.0 | 95.8 | 1.37 | 1.20 | 1.40 |
| Step2 | Step2 | 95.8 | 94.0 | 96.3 | 1.50 | 1.31 | 1.53 |
| Uniform | Normal | 95.4 | 94.6 | 95.9 | 1.38 | 1.20 | 1.41 |
| Uniform | U-shaped | 94.8 | 94.2 | 95.6 | 1.61 | 1.41 | 1.64 |
| Normal | U-shaped | 94.9 | 94.3 | 95.9 | 1.47 | 1.27 | 1.51 |
| Linear trend | Step2 | 95.2 | 91.4 | 96.1 | 1.44 | 1.26 | 1.48 |
| Step1 | Linear trend | 95.6 | 92.7 | 96.3 | 1.39 | 1.21 | 1.42 |
| Step1 | Step2 | 95.4 | 92.4 | 95.9 | 1.45 | 1.27 | 1.49 |
| Uniform | Step1 | 95.2 | 92.6 | 96.1 | 1.47 | 1.29 | 1.50 |
| Normal | Step1 | 94.6 | 92.3 | 95.6 | 1.30 | 1.13 | 1.33 |
| U-shaped | Step1 | 95.3 | 93.2 | 96.4 | 1.55 | 1.36 | 1.59 |
| Uniform | Linear trend | 95.3 | 92.8 | 95.8 | 1.46 | 1.27 | 1.49 |
| Normal | Linear trend | 95.1 | 92.2 | 95.1 | 1.28 | 1.12 | 1.32 |
| U-shaped | Linear trend | 95.1 | 92.4 | 95.8 | 1.54 | 1.34 | 1.58 |
| Uniform | Step2 | 94.8 | 92.3 | 95.5 | 1.52 | 1.33 | 1.57 |
| Normal | Step2 | 94.4 | 91.9 | 95.3 | 1.36 | 1.18 | 1.42 |
| U-shaped | Step2 | 95.5 | 93.0 | 96.3 | 1.60 | 1.39 | 1.64 |
| Mean coverage prob./interval length | | 95.2 | 93.2 | 95.8 | 1.45 | 1.27 | 1.49 |
| Mean deviation from 95% | | 0.34 | 1.78 | 0.83 | | | |

Web Table 39: Per cent coverage probabilities and interval lengths for the Welch U interval (U), the percentile bootstrap interval (PB), and the bootstrap-$t$ interval (Bt). For notation and definitions, see page 1.

## Outcome scale: {0, 1, 2}

$m = n = 50$

| Dist. $X$ ($m$) | Dist. $Y$ ($n$) | Coverage prob. U | PB | Bt | Interval length U | PB | Bt |
|---|---|---|---|---|---|---|---|
| Uniform | Uniform | 94.2 | 94.6 | 94.2 | 0.65 | 0.63 | 0.65 |
| Normal | Normal | 95.0 | 95.3 | 95.0 | 0.50 | 0.49 | 0.50 |
| U-shaped | U-shaped | 94.9 | 95.4 | 94.8 | 0.71 | 0.69 | 0.71 |
| Step1 | Step1 | 94.7 | 95.2 | 94.7 | 0.59 | 0.58 | 0.59 |
| Linear trend | Linear trend | 94.5 | 94.8 | 94.6 | 0.58 | 0.57 | 0.58 |
| Step2 | Step2 | 95.0 | 95.1 | 95.1 | 0.63 | 0.62 | 0.63 |
| Uniform | Normal | 94.9 | 95.3 | 95.0 | 0.58 | 0.57 | 0.58 |
| Uniform | U-shaped | 94.9 | 95.1 | 94.9 | 0.68 | 0.66 | 0.68 |
| Normal | U-shaped | 94.7 | 95.1 | 94.9 | 0.62 | 0.60 | 0.62 |
| Linear trend | Step2 | 94.8 | 94.2 | 94.7 | 0.61 | 0.59 | 0.61 |
| Step1 | Linear trend | 94.8 | 94.3 | 94.8 | 0.59 | 0.57 | 0.59 |
| Step1 | Step2 | 94.6 | 94.4 | 94.9 | 0.61 | 0.60 | 0.62 |
| Uniform | Step1 | 94.6 | 94.2 | 94.6 | 0.62 | 0.61 | 0.62 |
| Normal | Step1 | 95.0 | 94.6 | 94.9 | 0.55 | 0.54 | 0.55 |
| U-shaped | Step1 | 94.7 | 94.3 | 94.7 | 0.65 | 0.64 | 0.66 |
| Uniform | Linear trend | 94.7 | 94.1 | 94.6 | 0.61 | 0.60 | 0.62 |
| Normal | Linear trend | 94.6 | 94.1 | 94.6 | 0.54 | 0.53 | 0.54 |
| U-shaped | Linear trend | 94.9 | 94.4 | 94.9 | 0.65 | 0.63 | 0.65 |
| Uniform | Step2 | 94.9 | 94.6 | 94.9 | 0.64 | 0.63 | 0.64 |
| Normal | Step2 | 95.4 | 94.9 | 95.4 | 0.57 | 0.56 | 0.57 |
| U-shaped | Step2 | 94.6 | 94.3 | 94.6 | 0.67 | 0.66 | 0.67 |
| Mean coverage prob./interval length | | 94.8 | 94.7 | 94.8 | 0.61 | 0.60 | 0.61 |
| Mean deviation from 95% | | 0.25 | 0.47 | 0.26 | | | |

Web Table 40: Per cent coverage probabilities and interval lengths for the Welch U interval (U), the percentile bootstrap interval (PB), and the bootstrap-$t$ interval (Bt). For notation and definitions, see page 1.

## Outcome scale: {0, 1, 2}

$m = 25, n = 10$

| | | Coverage prob. | | | Interval length | | |
|---|---|---|---|---|---|---|---|
| Dist. $X$ $(m)$ | Dist. $Y$ $(n)$ | U | PB | Bt | U | PB | Bt |
| Uniform | Uniform | 94.7 | 93.1 | 94.5 | 1.29 | 1.14 | 1.26 |
| Normal | Normal | 94.9 | 92.4 | 94.7 | 0.99 | 0.87 | 0.96 |
| U-shaped | U-shaped | 94.0 | 92.7 | 93.7 | 1.41 | 1.25 | 1.38 |
| Step1 | Step1 | 94.4 | 92.9 | 93.8 | 1.18 | 1.04 | 1.15 |
| Linear trend | Linear trend | 94.8 | 93.4 | 94.2 | 1.14 | 1.01 | 1.12 |
| Step2 | Step2 | 94.0 | 92.8 | 93.5 | 1.25 | 1.11 | 1.23 |
| Uniform | Normal | 94.9 | 93.3 | 94.4 | 1.07 | 0.97 | 1.04 |
| Uniform | U-shaped | 93.7 | 92.4 | 93.6 | 1.38 | 1.21 | 1.36 |
| Normal | U-shaped | 94.0 | 92.6 | 94.6 | 1.33 | 1.14 | 1.33 |
| Normal | Uniform | 94.1 | 92.2 | 93.8 | 1.22 | 1.06 | 1.21 |
| U-shaped | Uniform | 94.5 | 93.5 | 94.3 | 1.31 | 1.17 | 1.28 |
| U-shaped | Normal | 95.2 | 94.0 | 95.1 | 1.10 | 1.00 | 1.08 |
| Linear trend | Step2 | 93.4 | 91.7 | 93.0 | 1.23 | 1.08 | 1.22 |
| Step1 | Linear trend | 94.9 | 92.5 | 94.5 | 1.15 | 1.02 | 1.13 |
| Step1 | Step2 | 93.8 | 92.0 | 93.4 | 1.24 | 1.09 | 1.23 |
| Uniform | Step1 | 94.5 | 92.7 | 94.1 | 1.20 | 1.07 | 1.17 |
| Normal | Step1 | 94.4 | 91.9 | 94.1 | 1.14 | 0.99 | 1.12 |
| U-shaped | Step1 | 94.2 | 92.4 | 93.8 | 1.23 | 1.11 | 1.20 |
| Uniform | Linear trend | 94.6 | 92.7 | 94.5 | 1.18 | 1.05 | 1.16 |
| Normal | Linear trend | 94.4 | 92.2 | 94.3 | 1.11 | 0.97 | 1.10 |
| U-shaped | Linear trend | 94.6 | 92.9 | 94.3 | 1.21 | 1.09 | 1.19 |
| Uniform | Step2 | 94.3 | 92.7 | 94.1 | 1.26 | 1.12 | 1.25 |
| Normal | Step2 | 93.4 | 92.0 | 93.5 | 1.20 | 1.04 | 1.22 |
| U-shaped | Step2 | 94.1 | 92.5 | 94.0 | 1.29 | 1.15 | 1.27 |
| Step2 | Linear trend | 94.6 | 92.3 | 94.1 | 1.17 | 1.05 | 1.15 |
| Linear trend | Step1 | 94.7 | 92.3 | 94.3 | 1.17 | 1.03 | 1.14 |
| Step2 | Step1 | 94.5 | 92.4 | 94.2 | 1.19 | 1.06 | 1.17 |
| Step1 | Uniform | 94.9 | 93.0 | 94.7 | 1.26 | 1.11 | 1.24 |
| Step1 | Normal | 94.8 | 92.1 | 94.3 | 1.04 | 0.93 | 1.01 |
| Step1 | U-shaped | 94.1 | 91.9 | 93.9 | 1.36 | 1.19 | 1.35 |
| Linear trend | Uniform | 94.2 | 92.3 | 94.0 | 1.26 | 1.10 | 1.24 |
| Linear trend | Normal | 94.4 | 91.8 | 94.0 | 1.03 | 0.92 | 1.00 |
| Linear trend | U-shaped | 93.5 | 91.7 | 93.6 | 1.36 | 1.18 | 1.34 |
| Step2 | Uniform | 94.4 | 92.6 | 94.2 | 1.28 | 1.13 | 1.26 |
| Step2 | Normal | 94.9 | 93.0 | 94.8 | 1.05 | 0.95 | 1.04 |
| Step2 | U-shaped | 93.7 | 92.3 | 93.8 | 1.38 | 1.21 | 1.36 |
| Mean coverage prob./interval length | | 94.3 | 92.5 | 94.1 | 1.21 | 1.07 | 1.19 |
| Mean deviation from 95% | | 0.67 | 2.47 | 0.90 | | | |

Web Table 41: Per cent coverage probabilities and interval lengths for the Welch U interval (U), the percentile bootstrap interval (PB), and the bootstrap-$t$ interval (Bt). For notation and definitions, see page 1.

## Outcome scale: {0, 1, 2}

$m = 100, n = 25$

| | | Coverage prob. | | | Interval length | | |
|---|---|---|---|---|---|---|---|
| Dist. $X$ $(m)$ | Dist. $Y$ $(n)$ | U | PB | Bt | U | PB | Bt |
| Uniform | Uniform | 94.9 | 94.4 | 94.4 | 0.74 | 0.70 | 0.73 |
| Normal | Normal | 95.2 | 94.5 | 94.7 | 0.57 | 0.54 | 0.56 |
| U-shaped | U-shaped | 95.3 | 94.7 | 95.0 | 0.81 | 0.77 | 0.80 |
| Step1 | Step1 | 95.2 | 94.9 | 94.7 | 0.68 | 0.64 | 0.66 |
| Linear trend | Linear trend | 94.7 | 94.3 | 94.3 | 0.66 | 0.63 | 0.65 |
| Step2 | Step2 | 95.3 | 94.9 | 95.0 | 0.72 | 0.69 | 0.71 |
| Uniform | Normal | 95.2 | 94.6 | 94.6 | 0.60 | 0.58 | 0.59 |
| Uniform | U-shaped | 95.2 | 94.7 | 94.7 | 0.80 | 0.75 | 0.79 |
| Normal | U-shaped | 94.9 | 94.5 | 94.6 | 0.77 | 0.73 | 0.77 |
| Normal | Uniform | 94.9 | 94.4 | 94.6 | 0.71 | 0.67 | 0.70 |
| U-shaped | Uniform | 95.2 | 94.9 | 94.7 | 0.75 | 0.72 | 0.74 |
| U-shaped | Normal | 95.1 | 94.8 | 94.8 | 0.62 | 0.60 | 0.61 |
| Linear trend | Step2 | 94.6 | 93.9 | 94.0 | 0.71 | 0.67 | 0.70 |
| Step1 | Linear trend | 95.1 | 94.3 | 94.7 | 0.66 | 0.63 | 0.65 |
| Step1 | Step2 | 94.5 | 94.1 | 94.2 | 0.71 | 0.68 | 0.70 |
| Uniform | Step1 | 95.0 | 94.0 | 94.5 | 0.69 | 0.65 | 0.68 |
| Normal | Step1 | 95.8 | 94.9 | 95.4 | 0.66 | 0.62 | 0.65 |
| U-shaped | Step1 | 94.9 | 94.3 | 94.6 | 0.70 | 0.67 | 0.69 |
| Uniform | Linear trend | 95.1 | 94.5 | 94.8 | 0.67 | 0.64 | 0.66 |
| Normal | Linear trend | 94.9 | 94.1 | 94.6 | 0.64 | 0.61 | 0.64 |
| U-shaped | Linear trend | 94.9 | 94.1 | 94.4 | 0.69 | 0.66 | 0.67 |
| Uniform | Step2 | 94.7 | 94.1 | 94.5 | 0.72 | 0.69 | 0.71 |
| Normal | Step2 | 94.8 | 94.1 | 94.7 | 0.70 | 0.66 | 0.69 |
| U-shaped | Step2 | 94.3 | 93.9 | 93.9 | 0.74 | 0.70 | 0.73 |
| Step2 | Linear trend | 94.9 | 94.0 | 94.4 | 0.67 | 0.64 | 0.66 |
| Linear trend | Step1 | 94.8 | 94.1 | 94.4 | 0.67 | 0.64 | 0.66 |
| Step2 | Step1 | 95.1 | 94.4 | 94.7 | 0.68 | 0.65 | 0.67 |
| Step1 | Uniform | 94.8 | 94.1 | 94.5 | 0.73 | 0.69 | 0.72 |
| Step1 | Normal | 95.3 | 94.6 | 94.9 | 0.59 | 0.56 | 0.58 |
| Step1 | U-shaped | 94.8 | 94.1 | 94.5 | 0.79 | 0.74 | 0.78 |
| Linear trend | Uniform | 94.7 | 93.9 | 94.3 | 0.73 | 0.69 | 0.71 |
| Linear trend | Normal | 95.5 | 94.3 | 95.0 | 0.59 | 0.56 | 0.58 |
| Linear trend | U-shaped | 94.3 | 93.5 | 94.1 | 0.79 | 0.74 | 0.78 |
| Step2 | Uniform | 94.8 | 94.2 | 94.6 | 0.74 | 0.70 | 0.72 |
| Step2 | Normal | 95.4 | 94.3 | 95.1 | 0.60 | 0.57 | 0.59 |
| Step2 | U-shaped | 94.8 | 93.9 | 94.5 | 0.80 | 0.75 | 0.78 |
| Mean coverage prob./interval length | | 95.0 | 94.3 | 94.6 | 0.70 | 0.66 | 0.69 |
| Mean deviation from 95% | | 0.26 | 0.69 | 0.44 | | | |

Web Table 42: Per cent coverage probabilities and interval lengths for the Welch U interval (U), the percentile bootstrap interval (PB), and the bootstrap-$t$ interval (Bt). For notation and definitions, see page 1.

## Outcome scale: {0, 1, 2, 3}

$m = n = 10$

| Dist. $X$ ($m$) | Dist. $Y$ ($n$) | Coverage prob. U | Coverage prob. PB | Coverage prob. Bt | Interval length U | Interval length PB | Interval length Bt |
|---|---|---|---|---|---|---|---|
| Uniform | Uniform | 94.7 | 93.6 | 95.3 | 2.10 | 1.84 | 2.13 |
| Normal | Normal | 95.2 | 93.7 | 95.4 | 1.73 | 1.51 | 1.75 |
| U-shaped | U-shaped | 95.1 | 94.3 | 96.1 | 2.56 | 2.25 | 2.61 |
| Linear trend | Linear trend | 95.2 | 93.5 | 95.6 | 1.87 | 1.64 | 1.91 |
| Step | Step | 95.6 | 93.7 | 96.0 | 1.76 | 1.54 | 1.81 |
| Skewed | Skewed | 95.8 | 93.1 | 96.4 | 1.60 | 1.39 | 1.68 |
| Uniform | Normal | 95.1 | 93.9 | 95.6 | 1.93 | 1.69 | 1.96 |
| Uniform | U-shaped | 95.0 | 94.1 | 95.9 | 2.35 | 2.06 | 2.40 |
| Normal | U-shaped | 94.9 | 94.1 | 96.2 | 2.20 | 1.91 | 2.28 |
| Linear trend | Step | 95.3 | 92.0 | 95.9 | 1.82 | 1.59 | 1.87 |
| Step | Skewed | 95.9 | 91.8 | 96.8 | 1.68 | 1.46 | 1.75 |
| Linear trend | Skewed | 95.3 | 91.9 | 95.8 | 1.75 | 1.53 | 1.80 |
| Uniform | Linear trend | 94.9 | 93.6 | 95.4 | 1.99 | 1.75 | 2.03 |
| Normal | Linear trend | 94.5 | 93.1 | 95.1 | 1.80 | 1.58 | 1.84 |
| U-shaped | Linear trend | 94.5 | 93.7 | 96.0 | 2.26 | 1.97 | 2.33 |
| Uniform | Step | 94.9 | 92.2 | 95.7 | 1.95 | 1.71 | 2.00 |
| Normal | Step | 94.8 | 92.0 | 95.2 | 1.74 | 1.53 | 1.79 |
| U-shaped | Step | 94.6 | 92.5 | 96.4 | 2.22 | 1.93 | 2.31 |
| Uniform | Skewed | 94.7 | 92.2 | 95.6 | 1.88 | 1.64 | 1.94 |
| Normal | Skewed | 94.8 | 92.4 | 95.6 | 1.67 | 1.46 | 1.73 |
| U-shaped | Skewed | 94.8 | 92.5 | 96.4 | 2.17 | 1.87 | 2.28 |
| Mean coverage prob./interval length | | 95.0 | 93.0 | 95.8 | 1.95 | 1.71 | 2.01 |
| Mean deviation from 95% | | 0.31 | 1.96 | 0.82 | | | |

Web Table 43: Per cent coverage probabilities and interval lengths for the Welch U interval (U), the percentile bootstrap interval (PB), and the bootstrap-$t$ interval (Bt). For notation and definitions, see page 1.

## Outcome scale: {0, 1, 2, 3}

$m = n = 50$

| Dist. $X$ ($m$) | Dist. $Y$ ($n$) | Coverage prob. U | PB | Bt | Interval length U | PB | Bt |
|---|---|---|---|---|---|---|---|
| Uniform | Uniform | 94.5 | 94.7 | 94.5 | 0.89 | 0.87 | 0.89 |
| Normal | Normal | 95.1 | 95.2 | 95.0 | 0.73 | 0.71 | 0.73 |
| U-shaped | U-shaped | 94.4 | 94.3 | 94.5 | 1.08 | 1.05 | 1.08 |
| Linear trend | Linear trend | 94.5 | 94.7 | 94.5 | 0.79 | 0.77 | 0.79 |
| Step | Step | 94.8 | 94.9 | 94.8 | 0.75 | 0.73 | 0.75 |
| Skewed | Skewed | 95.0 | 95.0 | 95.0 | 0.68 | 0.66 | 0.68 |
| Uniform | Normal | 95.1 | 95.1 | 95.0 | 0.81 | 0.79 | 0.81 |
| Uniform | U-shaped | 94.7 | 94.8 | 94.8 | 0.99 | 0.96 | 0.99 |
| Normal | U-shaped | 95.0 | 95.1 | 95.1 | 0.92 | 0.90 | 0.92 |
| Linear trend | Step | 95.1 | 94.6 | 95.1 | 0.77 | 0.75 | 0.77 |
| Step | Skewed | 94.6 | 94.0 | 94.6 | 0.71 | 0.70 | 0.71 |
| Linear trend | Skewed | 95.0 | 94.5 | 95.1 | 0.74 | 0.72 | 0.74 |
| Uniform | Linear trend | 94.7 | 94.7 | 94.8 | 0.84 | 0.82 | 0.84 |
| Normal | Linear trend | 94.1 | 94.3 | 94.3 | 0.76 | 0.74 | 0.76 |
| U-shaped | Linear trend | 94.7 | 94.5 | 94.7 | 0.95 | 0.92 | 0.95 |
| Uniform | Step | 94.5 | 94.2 | 94.6 | 0.82 | 0.80 | 0.82 |
| Normal | Step | 94.8 | 94.3 | 94.9 | 0.74 | 0.72 | 0.74 |
| U-shaped | Step | 94.5 | 94.0 | 94.7 | 0.93 | 0.91 | 0.93 |
| Uniform | Skewed | 94.9 | 94.7 | 94.9 | 0.79 | 0.77 | 0.79 |
| Normal | Skewed | 94.7 | 94.1 | 94.8 | 0.71 | 0.69 | 0.71 |
| U-shaped | Skewed | 95.3 | 94.8 | 95.3 | 0.90 | 0.88 | 0.91 |
| Mean coverage prob./interval length | | 94.8 | 94.6 | 94.8 | 0.82 | 0.80 | 0.82 |
| Mean deviation from 95% | | 0.30 | 0.44 | 0.26 | | | |

Web Table 44: Per cent coverage probabilities and interval lengths for the Welch U interval (U), the percentile bootstrap interval (PB), and the bootstrap-$t$ interval (Bt). For notation and definitions, see page 1.

## Outcome scale: {0, 1, 2, 3}

$m = 25, n = 10$

| Dist. $X$ ($m$) | Dist. $Y$ ($n$) | Coverage prob. U | PB | Bt | Interval length U | PB | Bt |
|---|---|---|---|---|---|---|---|
| Uniform | Uniform | 94.6 | 93.2 | 94.4 | 1.76 | 1.56 | 1.72 |
| Normal | Normal | 94.6 | 92.8 | 94.3 | 1.44 | 1.28 | 1.41 |
| U-shaped | U-shaped | 93.9 | 92.9 | 93.9 | 2.14 | 1.89 | 2.10 |
| Linear trend | Linear trend | 94.6 | 93.0 | 94.1 | 1.57 | 1.39 | 1.54 |
| Step | Step | 94.6 | 91.9 | 94.4 | 1.47 | 1.30 | 1.44 |
| Skewed | Skewed | 93.2 | 91.3 | 93.2 | 1.34 | 1.18 | 1.33 |
| Uniform | Normal | 94.3 | 92.9 | 93.9 | 1.52 | 1.37 | 1.49 |
| Uniform | U-shaped | 94.3 | 93.1 | 94.4 | 2.07 | 1.80 | 2.05 |
| Normal | U-shaped | 94.3 | 92.8 | 95.0 | 2.02 | 1.73 | 2.03 |
| Normal | Uniform | 94.7 | 92.7 | 94.3 | 1.69 | 1.48 | 1.67 |
| U-shaped | Uniform | 94.9 | 93.5 | 94.5 | 1.85 | 1.67 | 1.82 |
| U-shaped | Normal | 94.9 | 93.6 | 94.6 | 1.64 | 1.50 | 1.61 |
| Linear trend | Step | 94.6 | 92.1 | 94.3 | 1.49 | 1.33 | 1.46 |
| Step | Skewed | 94.4 | 91.9 | 94.2 | 1.37 | 1.22 | 1.35 |
| Linear trend | Skewed | 94.1 | 91.9 | 93.7 | 1.39 | 1.25 | 1.37 |
| Uniform | Linear trend | 94.8 | 93.5 | 94.5 | 1.61 | 1.44 | 1.58 |
| Normal | Linear trend | 94.4 | 92.5 | 94.0 | 1.54 | 1.35 | 1.52 |
| U-shaped | Linear trend | 95.2 | 93.6 | 94.8 | 1.72 | 1.56 | 1.69 |
| Uniform | Step | 94.1 | 92.2 | 93.9 | 1.54 | 1.38 | 1.51 |
| Normal | Step | 94.4 | 92.0 | 93.9 | 1.47 | 1.30 | 1.45 |
| U-shaped | Step | 94.5 | 92.8 | 94.4 | 1.65 | 1.51 | 1.63 |
| Uniform | Skewed | 93.9 | 91.6 | 93.8 | 1.45 | 1.31 | 1.43 |
| Normal | Skewed | 93.3 | 91.3 | 93.3 | 1.36 | 1.21 | 1.36 |
| U-shaped | Skewed | 94.7 | 93.0 | 94.5 | 1.57 | 1.44 | 1.55 |
| Step | Linear trend | 94.6 | 92.0 | 94.1 | 1.55 | 1.36 | 1.53 |
| Skewed | Step | 94.6 | 91.3 | 94.2 | 1.44 | 1.26 | 1.42 |
| Skewed | Linear trend | 94.8 | 92.0 | 94.4 | 1.52 | 1.33 | 1.50 |
| Linear trend | Uniform | 94.5 | 93.1 | 94.4 | 1.72 | 1.50 | 1.69 |
| Linear trend | Normal | 94.8 | 93.0 | 94.4 | 1.47 | 1.31 | 1.44 |
| Linear trend | U-shaped | 93.9 | 92.2 | 94.5 | 2.04 | 1.75 | 2.04 |
| Step | Uniform | 94.8 | 92.1 | 94.7 | 1.70 | 1.48 | 1.68 |
| Step | Normal | 94.6 | 91.9 | 94.2 | 1.45 | 1.29 | 1.43 |
| Step | U-shaped | 94.3 | 92.2 | 94.9 | 2.02 | 1.73 | 2.03 |
| Skewed | Uniform | 95.2 | 92.7 | 95.2 | 1.68 | 1.46 | 1.67 |
| Skewed | Normal | 95.0 | 92.2 | 95.0 | 1.43 | 1.25 | 1.41 |
| Skewed | U-shaped | 94.6 | 92.3 | 95.6 | 2.00 | 1.71 | 2.04 |
| Mean coverage prob./interval length | | 94.5 | 92.5 | 94.3 | 1.63 | 1.44 | 1.61 |
| Mean deviation from 95% | | 0.55 | 2.53 | 0.71 | | | |

Web Table 45: Per cent coverage probabilities and interval lengths for the Welch U interval (U), the percentile bootstrap interval (PB), and the bootstrap-$t$ interval (Bt). For notation and definitions, see page 1.

## Outcome scale: {0, 1, 2, 3}

$m = 100, n = 25$

| Dist. $X$ ($m$) | Dist. $Y$ ($n$) | Coverage prob. U | PB | Bt | Interval length U | PB | Bt |
|---|---|---|---|---|---|---|---|
| Uniform | Uniform | 95.2 | 94.6 | 94.8 | 1.01 | 0.96 | 0.99 |
| Normal | Normal | 95.1 | 94.5 | 94.6 | 0.83 | 0.79 | 0.82 |
| U-shaped | U-shaped | 94.9 | 94.6 | 94.7 | 1.23 | 1.17 | 1.21 |
| Linear trend | Linear trend | 95.0 | 94.3 | 94.7 | 0.90 | 0.86 | 0.89 |
| Step | Step | 95.1 | 94.6 | 94.7 | 0.85 | 0.81 | 0.84 |
| Skewed | Skewed | 94.3 | 93.4 | 93.9 | 0.77 | 0.73 | 0.76 |
| Uniform | Normal | 95.2 | 94.8 | 94.7 | 0.87 | 0.83 | 0.85 |
| Uniform | U-shaped | 95.1 | 94.5 | 94.9 | 1.20 | 1.13 | 1.18 |
| Normal | U-shaped | 95.1 | 94.6 | 95.0 | 1.17 | 1.10 | 1.16 |
| Normal | Uniform | 95.2 | 94.6 | 94.9 | 0.98 | 0.93 | 0.97 |
| U-shaped | Uniform | 94.7 | 94.2 | 94.4 | 1.05 | 1.01 | 1.03 |
| U-shaped | Normal | 95.2 | 94.6 | 94.8 | 0.92 | 0.88 | 0.90 |
| Linear trend | Step | 94.3 | 93.3 | 93.9 | 0.86 | 0.82 | 0.84 |
| Step | Skewed | 94.3 | 93.2 | 93.8 | 0.79 | 0.75 | 0.77 |
| Linear trend | Skewed | 94.4 | 93.8 | 94.0 | 0.80 | 0.76 | 0.78 |
| Uniform | Linear trend | 94.8 | 94.0 | 94.2 | 0.92 | 0.88 | 0.91 |
| Normal | Linear trend | 94.7 | 94.2 | 94.3 | 0.89 | 0.84 | 0.88 |
| U-shaped | Linear trend | 94.9 | 94.6 | 94.6 | 0.97 | 0.93 | 0.95 |
| Uniform | Step | 94.6 | 94.1 | 94.2 | 0.88 | 0.84 | 0.86 |
| Normal | Step | 94.5 | 93.5 | 94.0 | 0.84 | 0.80 | 0.83 |
| U-shaped | Step | 94.5 | 94.0 | 94.4 | 0.93 | 0.89 | 0.91 |
| Uniform | Skewed | 94.1 | 93.2 | 93.8 | 0.82 | 0.79 | 0.81 |
| Normal | Skewed | 93.9 | 93.2 | 93.5 | 0.78 | 0.74 | 0.77 |
| U-shaped | Skewed | 94.2 | 93.5 | 93.9 | 0.87 | 0.84 | 0.86 |
| Step | Linear trend | 94.7 | 93.9 | 94.4 | 0.89 | 0.85 | 0.88 |
| Skewed | Step | 94.4 | 93.5 | 94.1 | 0.84 | 0.79 | 0.82 |
| Skewed | Linear trend | 94.7 | 94.0 | 94.5 | 0.88 | 0.84 | 0.87 |
| Linear trend | Uniform | 95.0 | 94.1 | 94.6 | 0.99 | 0.94 | 0.98 |
| Linear trend | Normal | 95.3 | 94.6 | 95.0 | 0.85 | 0.81 | 0.83 |
| Linear trend | U-shaped | 94.7 | 93.9 | 94.5 | 1.18 | 1.11 | 1.17 |
| Step | Uniform | 95.0 | 94.1 | 94.6 | 0.99 | 0.93 | 0.97 |
| Step | Normal | 95.3 | 94.3 | 95.0 | 0.84 | 0.79 | 0.82 |
| Step | U-shaped | 94.8 | 93.9 | 94.6 | 1.18 | 1.10 | 1.17 |
| Skewed | Uniform | 94.8 | 93.7 | 94.5 | 0.97 | 0.92 | 0.96 |
| Skewed | Normal | 95.1 | 93.9 | 94.6 | 0.82 | 0.78 | 0.81 |
| Skewed | U-shaped | 95.1 | 94.2 | 95.0 | 1.17 | 1.09 | 1.16 |
| Mean coverage prob./interval length | | 94.8 | 94.0 | 94.4 | 0.94 | 0.89 | 0.92 |
| Mean deviation from 95% | | 0.32 | 0.95 | 0.56 | | | |

Web Table 46: Per cent coverage probabilities and interval lengths for the Welch U interval (U), the percentile bootstrap interval (PB), and the bootstrap-$t$ interval (Bt). For notation and definitions, see page 1.

## Outcome scale: {0, 1, 2, 3, 4}

$m = n = 10$

| | | Coverage prob. | | | Interval length | | |
|---|---|---|---|---|---|---|---|
| Dist. $X$ ($m$) | Dist. $Y$ ($n$) | U | PB | Bt | U | PB | Bt |
| Uniform | Uniform | 95.4 | 93.9 | 95.8 | 2.65 | 2.33 | 2.69 |
| Normal | Normal | 95.1 | 93.7 | 95.3 | 1.81 | 1.58 | 1.84 |
| U-shaped | U-shaped | 95.1 | 93.8 | 96.0 | 3.28 | 2.89 | 3.35 |
| Linear trend | Linear trend | 95.0 | 93.0 | 95.6 | 2.25 | 1.97 | 2.30 |
| Step | Step | 95.3 | 93.2 | 95.8 | 2.04 | 1.78 | 2.10 |
| Skewed | Skewed | 96.2 | 93.3 | 96.8 | 1.89 | 1.65 | 1.99 |
| Uniform | Normal | 94.4 | 93.3 | 95.0 | 2.29 | 1.99 | 2.35 |
| Uniform | U-shaped | 94.6 | 93.2 | 95.7 | 2.99 | 2.62 | 3.07 |
| Normal | U-shaped | 95.0 | 94.0 | 96.8 | 2.71 | 2.33 | 2.84 |
| Linear trend | Step | 95.2 | 91.6 | 95.6 | 2.15 | 1.88 | 2.20 |
| Step | Skewed | 95.2 | 91.4 | 96.0 | 1.97 | 1.72 | 2.05 |
| Linear trend | Skewed | 95.2 | 93.0 | 95.9 | 2.08 | 1.82 | 2.15 |
| Uniform | Linear trend | 95.0 | 92.5 | 95.7 | 2.47 | 2.16 | 2.52 |
| Normal | Linear trend | 94.2 | 91.2 | 94.7 | 2.04 | 1.78 | 2.10 |
| U-shaped | Linear trend | 94.4 | 92.6 | 96.0 | 2.84 | 2.47 | 2.94 |
| Uniform | Step | 95.2 | 92.7 | 95.7 | 2.38 | 2.08 | 2.45 |
| Normal | Step | 94.5 | 92.1 | 95.0 | 1.92 | 1.68 | 1.99 |
| U-shaped | Step | 94.7 | 92.6 | 96.5 | 2.78 | 2.40 | 2.90 |
| Uniform | Skewed | 94.7 | 92.0 | 95.7 | 2.33 | 2.02 | 2.41 |
| Normal | Skewed | 94.4 | 91.8 | 95.1 | 1.85 | 1.62 | 1.95 |
| U-shaped | Skewed | 94.4 | 92.5 | 96.6 | 2.74 | 2.36 | 2.88 |
| Mean coverage prob./interval length | | 94.9 | 92.7 | 95.8 | 2.36 | 2.05 | 2.43 |
| Mean deviation from 95% | | 0.36 | 2.28 | 0.80 | | | |

Web Table 47: Per cent coverage probabilities and interval lengths for the Welch U interval (U), the percentile bootstrap interval (PB), and the bootstrap-$t$ interval (Bt). For notation and definitions, see page 1.

## Outcome scale: {0, 1, 2, 3, 4}

$m = n = 50$

| Dist. $X$ ($m$) | Dist. $Y$ ($n$) | Coverage prob. U | Coverage prob. PB | Coverage prob. Bt | Interval length U | Interval length PB | Interval length Bt |
|---|---|---|---|---|---|---|---|
| Uniform | Uniform | 94.6 | 94.6 | 94.6 | 1.12 | 1.10 | 1.12 |
| Normal | Normal | 94.8 | 94.9 | 94.7 | 0.76 | 0.75 | 0.76 |
| U-shaped | U-shaped | 94.8 | 94.7 | 94.7 | 1.39 | 1.35 | 1.39 |
| Linear trend | Linear trend | 94.9 | 94.9 | 94.9 | 0.95 | 0.93 | 0.95 |
| Step | Step | 94.7 | 94.5 | 94.5 | 0.86 | 0.84 | 0.86 |
| Skewed | Skewed | 95.0 | 94.7 | 94.9 | 0.80 | 0.79 | 0.80 |
| Uniform | Normal | 94.9 | 94.6 | 94.9 | 0.96 | 0.94 | 0.96 |
| Uniform | U-shaped | 95.1 | 94.9 | 94.9 | 1.26 | 1.23 | 1.26 |
| Normal | U-shaped | 95.0 | 95.1 | 95.0 | 1.12 | 1.09 | 1.12 |
| Linear trend | Step | 94.2 | 93.7 | 94.1 | 0.91 | 0.89 | 0.91 |
| Step | Skewed | 94.7 | 94.2 | 94.8 | 0.84 | 0.82 | 0.84 |
| Linear trend | Skewed | 94.6 | 94.2 | 94.6 | 0.88 | 0.86 | 0.88 |
| Uniform | Linear trend | 94.7 | 94.4 | 94.8 | 1.04 | 1.02 | 1.04 |
| Normal | Linear trend | 95.2 | 94.9 | 95.1 | 0.86 | 0.84 | 0.86 |
| U-shaped | Linear trend | 94.8 | 94.3 | 94.7 | 1.19 | 1.16 | 1.19 |
| Uniform | Step | 95.0 | 94.4 | 95.0 | 1.00 | 0.98 | 1.00 |
| Normal | Step | 94.6 | 94.2 | 94.6 | 0.81 | 0.80 | 0.82 |
| U-shaped | Step | 94.3 | 93.8 | 94.4 | 1.16 | 1.13 | 1.16 |
| Uniform | Skewed | 95.1 | 94.7 | 95.2 | 0.98 | 0.96 | 0.98 |
| Normal | Skewed | 94.8 | 94.3 | 95.1 | 0.78 | 0.77 | 0.79 |
| U-shaped | Skewed | 94.9 | 94.6 | 95.0 | 1.14 | 1.11 | 1.14 |
| Mean coverage prob./interval length | | 94.8 | 94.5 | 94.8 | 0.99 | 0.97 | 0.99 |
| Mean deviation from 95% | | 0.24 | 0.51 | 0.25 | | | |

Web Table 48: Per cent coverage probabilities and interval lengths for the Welch U interval (U), the percentile bootstrap interval (PB), and the bootstrap-$t$ interval (Bt). For notation and definitions, see page 1.

## Outcome scale: {0, 1, 2, 3, 4}

$m = 25, n = 10$

| | | Coverage prob. | | | Interval length | | |
|---|---|---|---|---|---|---|---|
| Dist. $X$ ($m$) | Dist. $Y$ ($n$) | U | PB | Bt | U | PB | Bt |
| Uniform | Uniform | 94.2 | 92.5 | 94.0 | 2.21 | 1.96 | 2.17 |
| Normal | Normal | 95.2 | 93.1 | 94.8 | 1.50 | 1.33 | 1.47 |
| U-shaped | U-shaped | 94.6 | 93.3 | 94.4 | 2.76 | 2.44 | 2.71 |
| Linear trend | Linear trend | 94.0 | 92.1 | 93.7 | 1.87 | 1.66 | 1.84 |
| Step | Step | 94.5 | 92.1 | 94.3 | 1.69 | 1.50 | 1.67 |
| Skewed | Skewed | 94.1 | 91.9 | 94.2 | 1.58 | 1.39 | 1.58 |
| Uniform | Normal | 95.2 | 93.9 | 95.1 | 1.70 | 1.55 | 1.67 |
| Uniform | U-shaped | 94.4 | 92.9 | 94.6 | 2.65 | 2.30 | 2.63 |
| Normal | U-shaped | 94.6 | 93.1 | 96.0 | 2.56 | 2.16 | 2.61 |
| Normal | Uniform | 94.6 | 92.3 | 94.5 | 2.09 | 1.79 | 2.07 |
| U-shaped | Uniform | 94.7 | 93.1 | 94.4 | 2.35 | 2.12 | 2.30 |
| U-shaped | Normal | 95.2 | 93.9 | 95.2 | 1.89 | 1.74 | 1.87 |
| Linear trend | Step | 94.7 | 91.9 | 94.2 | 1.74 | 1.56 | 1.71 |
| Step | Skewed | 94.2 | 91.6 | 94.1 | 1.61 | 1.43 | 1.60 |
| Linear trend | Skewed | 93.8 | 91.5 | 93.5 | 1.65 | 1.48 | 1.63 |
| Uniform | Linear trend | 94.6 | 92.9 | 94.3 | 1.96 | 1.76 | 1.93 |
| Normal | Linear trend | 93.9 | 91.2 | 93.6 | 1.80 | 1.57 | 1.79 |
| U-shaped | Linear trend | 94.5 | 93.0 | 94.4 | 2.12 | 1.93 | 2.09 |
| Uniform | Step | 94.7 | 92.9 | 94.5 | 1.83 | 1.66 | 1.80 |
| Normal | Step | 93.9 | 91.6 | 93.5 | 1.65 | 1.45 | 1.64 |
| U-shaped | Step | 95.5 | 93.9 | 95.4 | 2.01 | 1.84 | 1.98 |
| Uniform | Skewed | 95.0 | 93.0 | 94.7 | 1.76 | 1.59 | 1.74 |
| Normal | Skewed | 93.4 | 91.0 | 93.1 | 1.56 | 1.37 | 1.58 |
| U-shaped | Skewed | 95.2 | 93.6 | 95.2 | 1.94 | 1.78 | 1.92 |
| Step | Linear trend | 93.9 | 91.1 | 93.6 | 1.84 | 1.62 | 1.82 |
| Skewed | Step | 94.3 | 91.3 | 94.2 | 1.67 | 1.47 | 1.66 |
| Skewed | Linear trend | 94.4 | 92.0 | 94.0 | 1.82 | 1.59 | 1.80 |
| Linear trend | Uniform | 94.5 | 92.2 | 94.3 | 2.15 | 1.88 | 2.12 |
| Linear trend | Normal | 95.5 | 93.3 | 95.2 | 1.60 | 1.44 | 1.57 |
| Linear trend | U-shaped | 94.5 | 92.7 | 95.1 | 2.60 | 2.23 | 2.61 |
| Step | Uniform | 94.2 | 92.1 | 93.9 | 2.12 | 1.84 | 2.10 |
| Step | Normal | 95.2 | 92.8 | 95.0 | 1.55 | 1.39 | 1.53 |
| Step | U-shaped | 94.7 | 93.0 | 95.7 | 2.58 | 2.20 | 2.62 |
| Skewed | Uniform | 94.3 | 91.6 | 94.1 | 2.10 | 1.81 | 2.09 |
| Skewed | Normal | 94.7 | 92.2 | 94.6 | 1.53 | 1.36 | 1.52 |
| Skewed | U-shaped | 94.0 | 92.1 | 95.6 | 2.56 | 2.17 | 2.62 |
| Mean coverage prob./interval length | | 94.5 | 92.5 | 94.5 | 1.96 | 1.73 | 1.95 |
| Mean deviation from 95% | | 0.60 | 2.55 | 0.72 | | | |

Web Table 49: Per cent coverage probabilities and interval lengths for the Welch U interval (U), the percentile bootstrap interval (PB), and the bootstrap-$t$ interval (Bt). For notation and definitions, see page 1.

## Outcome scale: {0, 1, 2, 3, 4}

$m = 100, n = 25$

| | | Coverage prob. | | | Interval length | | |
|---|---|---|---|---|---|---|---|
| Dist. $X$ ($m$) | Dist. $Y$ ($n$) | U | PB | Bt | U | PB | Bt |
| Uniform | Uniform | 95.1 | 94.3 | 94.5 | 1.28 | 1.21 | 1.26 |
| Normal | Normal | 94.9 | 94.0 | 94.4 | 0.87 | 0.83 | 0.85 |
| U-shaped | U-shaped | 94.8 | 94.1 | 94.1 | 1.58 | 1.50 | 1.55 |
| Linear trend | Linear trend | 94.8 | 94.1 | 94.5 | 1.08 | 1.03 | 1.06 |
| Step | Step | 94.5 | 93.8 | 94.0 | 0.98 | 0.93 | 0.97 |
| Skewed | Skewed | 94.2 | 93.8 | 93.9 | 0.91 | 0.87 | 0.90 |
| Uniform | Normal | 95.1 | 94.7 | 94.9 | 0.96 | 0.92 | 0.94 |
| Uniform | U-shaped | 95.1 | 94.6 | 94.9 | 1.53 | 1.45 | 1.51 |
| Normal | U-shaped | 94.7 | 94.1 | 94.7 | 1.48 | 1.39 | 1.48 |
| Normal | Uniform | 95.0 | 94.3 | 94.8 | 1.22 | 1.14 | 1.20 |
| U-shaped | Uniform | 94.9 | 94.4 | 94.3 | 1.34 | 1.28 | 1.31 |
| U-shaped | Normal | 94.6 | 94.2 | 94.3 | 1.04 | 1.00 | 1.02 |
| Linear trend | Step | 94.7 | 93.8 | 94.3 | 1.00 | 0.95 | 0.98 |
| Step | Skewed | 94.1 | 93.5 | 93.8 | 0.93 | 0.88 | 0.91 |
| Linear trend | Skewed | 94.2 | 93.5 | 93.8 | 0.95 | 0.90 | 0.93 |
| Uniform | Linear trend | 94.8 | 94.3 | 94.6 | 1.12 | 1.07 | 1.10 |
| Normal | Linear trend | 95.2 | 94.6 | 94.9 | 1.05 | 0.99 | 1.03 |
| U-shaped | Linear trend | 94.8 | 94.3 | 94.4 | 1.19 | 1.14 | 1.17 |
| Uniform | Step | 94.5 | 93.8 | 94.1 | 1.04 | 1.00 | 1.02 |
| Normal | Step | 94.0 | 93.3 | 93.7 | 0.96 | 0.91 | 0.95 |
| U-shaped | Step | 94.9 | 94.3 | 94.5 | 1.11 | 1.07 | 1.09 |
| Uniform | Skewed | 94.6 | 94.1 | 94.2 | 0.99 | 0.95 | 0.97 |
| Normal | Skewed | 94.1 | 93.5 | 93.9 | 0.91 | 0.86 | 0.90 |
| U-shaped | Skewed | 94.4 | 93.7 | 94.1 | 1.07 | 1.03 | 1.05 |
| Step | Linear trend | 94.7 | 93.9 | 94.4 | 1.07 | 1.01 | 1.05 |
| Skewed | Step | 94.5 | 93.4 | 94.2 | 0.97 | 0.92 | 0.95 |
| Skewed | Linear trend | 95.2 | 94.5 | 94.9 | 1.06 | 1.00 | 1.04 |
| Linear trend | Uniform | 94.7 | 94.1 | 94.3 | 1.25 | 1.18 | 1.23 |
| Linear trend | Normal | 95.0 | 94.4 | 94.6 | 0.91 | 0.87 | 0.89 |
| Linear trend | U-shaped | 94.7 | 94.1 | 94.4 | 1.51 | 1.41 | 1.49 |
| Step | Uniform | 95.0 | 94.1 | 94.7 | 1.23 | 1.16 | 1.21 |
| Step | Normal | 95.0 | 94.0 | 94.7 | 0.89 | 0.85 | 0.87 |
| Step | U-shaped | 94.8 | 94.0 | 94.7 | 1.49 | 1.40 | 1.48 |
| Skewed | Uniform | 95.0 | 94.1 | 94.8 | 1.22 | 1.15 | 1.21 |
| Skewed | Normal | 95.1 | 94.3 | 94.8 | 0.88 | 0.84 | 0.86 |
| Skewed | U-shaped | 94.7 | 94.0 | 94.8 | 1.49 | 1.39 | 1.48 |
| Mean coverage prob./interval length | | 94.7 | 94.1 | 94.4 | 1.13 | 1.07 | 1.11 |
| Mean deviation from 95% | | 0.32 | 0.95 | 0.59 | | | |

Web Table 50: Per cent coverage probabilities and interval lengths for the Welch U interval (U), the percentile bootstrap interval (PB), and the bootstrap-$t$ interval (Bt). For notation and definitions, see page 1.

## Outcome scale: {0, 1, 2, 3, 4, 5}

$m = n = 10$

| Dist. $X$ ($m$) | Dist. $Y$ ($n$) | Coverage prob. U | PB | Bt | Interval length U | PB | Bt |
|---|---|---|---|---|---|---|---|
| Uniform | Uniform | 95.3 | 94.0 | 95.8 | 3.21 | 2.82 | 3.26 |
| Normal | Normal | 95.5 | 93.9 | 95.6 | 1.86 | 1.62 | 1.89 |
| U-shaped | U-shaped | 95.2 | 93.4 | 96.0 | 4.06 | 3.57 | 4.14 |
| Linear trend | Linear trend | 94.9 | 93.3 | 95.2 | 2.74 | 2.40 | 2.79 |
| Step | Step | 95.4 | 93.0 | 95.9 | 2.71 | 2.37 | 2.78 |
| Skewed | Skewed | 95.7 | 92.4 | 96.6 | 2.12 | 1.85 | 2.26 |
| Uniform | Normal | 94.7 | 93.5 | 95.4 | 2.66 | 2.29 | 2.73 |
| Uniform | U-shaped | 95.0 | 93.6 | 96.0 | 3.67 | 3.21 | 3.76 |
| Normal | U-shaped | 94.6 | 93.4 | 97.0 | 3.24 | 2.76 | 3.45 |
| Linear trend | Step | 95.0 | 91.8 | 95.4 | 2.73 | 2.39 | 2.79 |
| Step | Skewed | 95.3 | 91.2 | 95.8 | 2.45 | 2.13 | 2.55 |
| Linear trend | Skewed | 94.9 | 91.3 | 95.6 | 2.46 | 2.14 | 2.55 |
| Uniform | Linear trend | 95.5 | 92.8 | 96.1 | 2.99 | 2.62 | 3.05 |
| Normal | Linear trend | 94.5 | 91.5 | 94.9 | 2.36 | 2.05 | 2.45 |
| U-shaped | Linear trend | 94.6 | 92.6 | 96.0 | 3.50 | 3.04 | 3.62 |
| Uniform | Step | 95.3 | 92.7 | 95.9 | 2.98 | 2.60 | 3.05 |
| Normal | Step | 94.4 | 91.6 | 94.9 | 2.33 | 2.02 | 2.44 |
| U-shaped | Step | 94.7 | 92.3 | 96.2 | 3.49 | 3.03 | 3.63 |
| Uniform | Skewed | 94.7 | 91.7 | 95.6 | 2.75 | 2.38 | 2.84 |
| Normal | Skewed | 94.2 | 91.1 | 95.0 | 1.99 | 1.73 | 2.13 |
| U-shaped | Skewed | 94.4 | 92.2 | 97.0 | 3.32 | 2.84 | 3.52 |
| Mean coverage prob./interval length | | 94.9 | 92.5 | 95.8 | 2.84 | 2.47 | 2.94 |
| Mean deviation from 95% | | 0.36 | 2.48 | 0.83 | | | |

Web Table 51: Per cent coverage probabilities and interval lengths for the Welch U interval (U), the percentile bootstrap interval (PB), and the bootstrap-$t$ interval (Bt). For notation and definitions, see page 1.

## Outcome scale: {0, 1, 2, 3, 4, 5}

$m = n = 50$

| Dist. $X$ ($m$) | Dist. $Y$ ($n$) | Coverage prob. U | PB | Bt | Interval length U | PB | Bt |
|---|---|---|---|---|---|---|---|
| Uniform | Uniform | 94.6 | 94.6 | 94.5 | 1.36 | 1.32 | 1.35 |
| Normal | Normal | 94.9 | 94.8 | 94.8 | 0.79 | 0.77 | 0.79 |
| U-shaped | U-shaped | 94.7 | 94.7 | 94.7 | 1.71 | 1.67 | 1.71 |
| Linear trend | Linear trend | 95.1 | 95.0 | 94.9 | 1.16 | 1.13 | 1.16 |
| Step | Step | 94.2 | 94.2 | 94.3 | 1.15 | 1.12 | 1.15 |
| Skewed | Skewed | 95.2 | 94.9 | 95.2 | 0.90 | 0.88 | 0.91 |
| Uniform | Normal | 94.6 | 94.6 | 94.5 | 1.11 | 1.08 | 1.11 |
| Uniform | U-shaped | 95.0 | 94.9 | 94.9 | 1.54 | 1.51 | 1.54 |
| Normal | U-shaped | 95.1 | 95.0 | 95.1 | 1.34 | 1.30 | 1.34 |
| Linear trend | Step | 95.0 | 94.4 | 94.9 | 1.16 | 1.13 | 1.16 |
| Step | Skewed | 95.1 | 94.8 | 95.2 | 1.04 | 1.01 | 1.04 |
| Linear trend | Skewed | 94.8 | 94.2 | 94.7 | 1.04 | 1.02 | 1.04 |
| Uniform | Linear trend | 94.9 | 94.6 | 94.8 | 1.26 | 1.23 | 1.26 |
| Normal | Linear trend | 94.7 | 94.2 | 94.8 | 1.00 | 0.97 | 1.00 |
| U-shaped | Linear trend | 94.4 | 93.9 | 94.4 | 1.47 | 1.43 | 1.47 |
| Uniform | Step | 94.5 | 94.3 | 94.7 | 1.26 | 1.23 | 1.26 |
| Normal | Step | 94.3 | 93.7 | 94.4 | 0.99 | 0.96 | 0.99 |
| U-shaped | Step | 94.7 | 94.4 | 94.8 | 1.46 | 1.42 | 1.46 |
| Uniform | Skewed | 94.6 | 94.3 | 94.6 | 1.16 | 1.13 | 1.16 |
| Normal | Skewed | 94.8 | 94.4 | 94.9 | 0.85 | 0.83 | 0.86 |
| U-shaped | Skewed | 94.7 | 94.5 | 94.8 | 1.38 | 1.34 | 1.38 |
| Mean coverage prob./interval length | | 94.8 | 94.5 | 94.8 | 1.20 | 1.17 | 1.20 |
| Mean deviation from 95% | | 0.29 | 0.52 | 0.29 | | | |

Web Table 52: Per cent coverage probabilities and interval lengths for the Welch U interval (U), the percentile bootstrap interval (PB), and the bootstrap-$t$ interval (Bt). For notation and definitions, see page 1.

## Outcome scale: {0, 1, 2, 3, 4, 5}

$m = 25, n = 10$

| | | Coverage prob. | | | Interval length | | |
|---|---|---|---|---|---|---|---|
| Dist. $X$ ($m$) | Dist. $Y$ ($n$) | U | PB | Bt | U | PB | Bt |
| Uniform | Uniform | 94.7 | 92.9 | 94.5 | 2.69 | 2.38 | 2.63 |
| Normal | Normal | 95.2 | 92.9 | 94.8 | 1.55 | 1.37 | 1.52 |
| U-shaped | U-shaped | 95.0 | 93.3 | 94.5 | 3.39 | 3.00 | 3.33 |
| Linear trend | Linear trend | 94.3 | 92.0 | 93.8 | 2.29 | 2.03 | 2.25 |
| Step | Step | 93.7 | 92.0 | 93.5 | 2.27 | 2.01 | 2.23 |
| Skewed | Skewed | 93.8 | 91.5 | 94.1 | 1.77 | 1.57 | 1.78 |
| Uniform | Normal | 95.1 | 93.6 | 94.8 | 1.89 | 1.74 | 1.87 |
| Uniform | U-shaped | 94.3 | 92.8 | 94.5 | 3.26 | 2.83 | 3.23 |
| Normal | U-shaped | 94.4 | 92.6 | 96.4 | 3.12 | 2.61 | 3.24 |
| Normal | Uniform | 94.7 | 92.4 | 94.9 | 2.50 | 2.12 | 2.49 |
| U-shaped | Uniform | 95.1 | 93.7 | 94.7 | 2.87 | 2.59 | 2.81 |
| U-shaped | Normal | 94.9 | 93.8 | 94.9 | 2.17 | 2.01 | 2.16 |
| Linear trend | Step | 94.3 | 92.1 | 94.0 | 2.28 | 2.02 | 2.23 |
| Step | Skewed | 94.9 | 92.2 | 94.8 | 1.91 | 1.72 | 1.89 |
| Linear trend | Skewed | 94.6 | 92.2 | 94.5 | 1.91 | 1.72 | 1.88 |
| Uniform | Linear trend | 94.7 | 92.5 | 94.2 | 2.39 | 2.14 | 2.34 |
| Normal | Linear trend | 94.1 | 91.9 | 93.9 | 2.16 | 1.85 | 2.16 |
| U-shaped | Linear trend | 95.0 | 93.4 | 94.7 | 2.60 | 2.37 | 2.56 |
| Uniform | Step | 94.5 | 92.5 | 94.2 | 2.37 | 2.13 | 2.32 |
| Normal | Step | 93.6 | 90.9 | 93.2 | 2.12 | 1.82 | 2.14 |
| U-shaped | Step | 95.1 | 93.5 | 95.0 | 2.58 | 2.36 | 2.54 |
| Uniform | Skewed | 93.9 | 92.5 | 93.9 | 2.03 | 1.85 | 2.01 |
| Normal | Skewed | 93.3 | 91.3 | 93.1 | 1.72 | 1.50 | 1.76 |
| U-shaped | Skewed | 95.3 | 94.0 | 95.4 | 2.30 | 2.13 | 2.29 |
| Step | Linear trend | 94.4 | 92.2 | 94.1 | 2.29 | 2.03 | 2.25 |
| Skewed | Step | 93.9 | 91.4 | 93.7 | 2.17 | 1.88 | 2.16 |
| Skewed | Linear trend | 94.4 | 91.9 | 94.1 | 2.20 | 1.91 | 2.19 |
| Linear trend | Uniform | 95.3 | 93.0 | 94.9 | 2.61 | 2.28 | 2.57 |
| Linear trend | Normal | 94.6 | 93.0 | 94.4 | 1.76 | 1.60 | 1.74 |
| Linear trend | U-shaped | 94.3 | 92.7 | 95.2 | 3.21 | 2.75 | 3.22 |
| Step | Uniform | 95.0 | 93.0 | 94.9 | 2.60 | 2.27 | 2.56 |
| Step | Normal | 95.4 | 92.8 | 95.3 | 1.75 | 1.59 | 1.74 |
| Step | U-shaped | 94.5 | 92.6 | 95.1 | 3.20 | 2.74 | 3.23 |
| Skewed | Uniform | 94.3 | 91.7 | 94.3 | 2.52 | 2.16 | 2.52 |
| Skewed | Normal | 94.5 | 92.2 | 94.8 | 1.61 | 1.45 | 1.61 |
| Skewed | U-shaped | 93.9 | 92.4 | 95.7 | 3.14 | 2.65 | 3.24 |
| Mean coverage prob./interval length | | 94.5 | 92.5 | 94.5 | 2.37 | 2.09 | 2.35 |
| Mean deviation from 95% | | 0.55 | 2.46 | 0.65 | | | |

Web Table 53: Per cent coverage probabilities and interval lengths for the Welch U interval (U), the percentile bootstrap interval (PB), and the bootstrap-$t$ interval (Bt). For notation and definitions, see page 1.

## Outcome scale: {0, 1, 2, 3, 4, 5}

$m = 100, n = 25$

| Dist. $X$ ($m$) | Dist. $Y$ ($n$) | Coverage prob. U | PB | Bt | Interval length U | PB | Bt |
|---|---|---|---|---|---|---|---|
| Uniform | Uniform | 95.2 | 94.6 | 94.7 | 1.54 | 1.47 | 1.52 |
| Normal | Normal | 95.0 | 94.2 | 94.5 | 0.89 | 0.85 | 0.87 |
| U-shaped | U-shaped | 94.7 | 94.2 | 94.6 | 1.95 | 1.85 | 1.92 |
| Linear trend | Linear trend | 94.5 | 93.8 | 94.1 | 1.32 | 1.25 | 1.30 |
| Step | Step | 94.7 | 94.0 | 94.2 | 1.30 | 1.24 | 1.28 |
| Skewed | Skewed | 94.2 | 93.4 | 93.8 | 1.03 | 0.97 | 1.01 |
| Uniform | Normal | 94.8 | 94.2 | 94.4 | 1.04 | 1.01 | 1.03 |
| Uniform | U-shaped | 95.1 | 94.3 | 94.9 | 1.89 | 1.78 | 1.86 |
| Normal | U-shaped | 94.6 | 94.0 | 94.8 | 1.81 | 1.69 | 1.81 |
| Normal | Uniform | 94.9 | 94.2 | 94.7 | 1.45 | 1.36 | 1.44 |
| U-shaped | Uniform | 95.1 | 94.6 | 94.5 | 1.62 | 1.55 | 1.59 |
| U-shaped | Normal | 95.4 | 95.1 | 95.2 | 1.16 | 1.13 | 1.15 |
| Linear trend | Step | 94.8 | 93.7 | 94.3 | 1.31 | 1.24 | 1.28 |
| Step | Skewed | 94.8 | 94.2 | 94.6 | 1.08 | 1.04 | 1.06 |
| Linear trend | Skewed | 94.0 | 93.2 | 93.2 | 1.09 | 1.04 | 1.07 |
| Uniform | Linear trend | 94.4 | 93.4 | 94.0 | 1.37 | 1.30 | 1.34 |
| Normal | Linear trend | 94.8 | 93.8 | 94.4 | 1.26 | 1.18 | 1.25 |
| U-shaped | Linear trend | 94.8 | 94.2 | 94.3 | 1.45 | 1.40 | 1.43 |
| Uniform | Step | 94.7 | 94.0 | 94.3 | 1.35 | 1.29 | 1.32 |
| Normal | Step | 94.3 | 93.1 | 94.0 | 1.24 | 1.16 | 1.23 |
| U-shaped | Step | 94.7 | 93.8 | 94.1 | 1.44 | 1.39 | 1.42 |
| Uniform | Skewed | 93.9 | 93.5 | 93.5 | 1.14 | 1.09 | 1.12 |
| Normal | Skewed | 93.6 | 93.2 | 93.3 | 1.00 | 0.95 | 1.00 |
| U-shaped | Skewed | 94.5 | 93.8 | 94.1 | 1.25 | 1.21 | 1.23 |
| Step | Linear trend | 94.4 | 93.7 | 94.0 | 1.32 | 1.25 | 1.29 |
| Skewed | Step | 94.4 | 93.5 | 93.9 | 1.26 | 1.19 | 1.24 |
| Skewed | Linear trend | 94.8 | 94.0 | 94.4 | 1.28 | 1.20 | 1.26 |
| Linear trend | Uniform | 94.9 | 94.0 | 94.4 | 1.51 | 1.43 | 1.48 |
| Linear trend | Normal | 95.1 | 94.2 | 94.8 | 0.99 | 0.95 | 0.97 |
| Linear trend | U-shaped | 94.9 | 94.0 | 94.8 | 1.86 | 1.74 | 1.84 |
| Step | Uniform | 94.8 | 94.1 | 94.6 | 1.50 | 1.42 | 1.48 |
| Step | Normal | 95.2 | 94.5 | 95.0 | 0.98 | 0.95 | 0.97 |
| Step | U-shaped | 95.2 | 94.6 | 95.3 | 1.86 | 1.74 | 1.84 |
| Skewed | Uniform | 95.1 | 93.9 | 94.9 | 1.47 | 1.38 | 1.45 |
| Skewed | Normal | 95.1 | 94.3 | 94.7 | 0.92 | 0.88 | 0.91 |
| Skewed | U-shaped | 94.6 | 93.9 | 94.7 | 1.83 | 1.71 | 1.82 |
| Mean coverage prob./interval length | | 94.7 | 94.0 | 94.4 | 1.35 | 1.29 | 1.34 |
| Mean deviation from 95% | | 0.37 | 1.04 | 0.64 | | | |

Web Table 54: Per cent coverage probabilities and interval lengths for the Welch U interval (U), the percentile bootstrap interval (PB), and the bootstrap-$t$ interval (Bt). For notation and definitions, see page 1.
